# Network Pharmacology and Molecular Docking Analysis on Molecular Mechanism of Qingzi Zhitong Decoction in the Treatment of Ulcerative Colitis

**DOI:** 10.3389/fphar.2022.727608

**Published:** 2022-02-08

**Authors:** Xintian Shou, Yumeng Wang, Xuesong Zhang, Yanju Zhang, Yan Yang, Chenglin Duan, Yihan Yang, Qiulei Jia, Guozhen Yuan, Jingjing Shi, Shuqing Shi, Hanming Cui, Yuanhui Hu

**Affiliations:** ^1^ China Academy of Chinese Medical Sciences Guang’anmen Hospital, Beijing, China; ^2^ Beijing University of Chinese Medicine, Beijing, China; ^3^ National Center for Children’s Health, Beijing Children’s Hospital, Capital Medical University, Beijing, China

**Keywords:** network pharmacology, molecular docking, molecular mechanism, ulcerative colitis, Qingzi Zhitong Decoction

## Abstract

Ulcerative colitis (UC) is a disease with complex pathological mechanisms. We explored the potential molecular mechanisms behind the therapeutic functions of Qingzi Zhitong decoction (QZZTD) in the treatment of UC by network pharmacology and molecular docking. QZZTD is a formula of Chinese traditional medicine consisting of 10 herbs. The potential active ingredients of QZZTD and their target genes were obtained from the Traditional Chinese Medicine Systems Pharmacology Database and Analysis Platform database, and UC-related target genes were obtained from GeneCards and OMIM databases. A total of 138 co-identified target genes were obtained by plotting the intersection target Venn diagram, and then the STRING database and Cytoscape software were used to establish protein–protein interaction networks and herb–ingredient–target networks. Four key active compounds and nine key proteins were identified. Then, Gene Ontology and Kyoto Encyclopedia of Genes and Genomes enrichment analyses showed that the biological functions of potential target genes were associated with DNA transcription, signaling receptor and ligand activity, cytokine activity, cellular autophagy, and antioxidant pathways, with related pathways involving the phosphatidylinositol 3-kinase (PI3K)–Akt signaling pathway, advanced glycosylation end product (AGE)–RAGE signaling pathway, tumor necrosis factor (TNF) signaling pathway, and IL-17 signaling pathway. Moreover, the binding activities of key target genes and essential active compounds of Chinese herbal medicines in QZZTD were further validated by molecular docking. This demonstrated that quercetin, luteolin, hyndarin, and beta-sitosterol had good binding to eight key proteins, and Akt1 was the target protein with the best binding activity, suggesting that Akt1 could be the essential mediator responsible for signaling transduction after QZZTD administration. The rat experiment verified that QZZTD inhibited PI3K-Akt pathway activation and reduced inflammation in UC. In conclusion, our study suggested four potential key active components, including quercetin, were identified in QZZTD, which could interact with Akt1 and modulate the activation of the PI3K-Akt pathway. The other three pathways may also be involved in the signaling transduction induced by QZZTD in the treatment of UC.

## 1 Introduction

Ulcerative colitis (UC) is a chronic idiopathic inflammation of the colonic mucosa that gradually affects the rectum and part or the entire colon (Ordas et al., 2012). Clinical presentations are usually accompanied by fecal urgency or blood in stool, since extensive colitis or pancolitis may cause diarrhea, frequent passage of blood and mucus, bowel movement urgency or tenesmus, abdominal pain, fever, malaise, and weight loss, but constipation may also occur paradoxically. The incidence of the disease is increasing worldwide. According to the ACG Clinical Guideline, there are approximately 1 million individuals affected by this condition each in the United States and Europe and many more globally, which also results in a heavy pecuniary burden for patients’ family and society (Rubin et al., 2019). The pathogenesis of UC is complex, involving genetics, mucosal immunity, epithelial barrier defects, and autoimmunity. Studies using genetic risk scores (GRS) to distinguish genetic contributions to inflammatory bowel disease phenotypes have shown that UC GRS (R^2^ = 7.1%, FDR = 0.02) is associated with colonic Crohn’s disease, which is largely driven by genetic variation in the major histocompatibility complex (Voskuil et al., 2020). An altered tight junction structure contributes to the impaired epithelial barrier function in UC (Schmitz et al., 1999). Both innate and adaptive immunity play a crucial role in the pathogenesis of UC, but we currently have a limited understanding of it.

Qingzi Zhitong Decoction (QZZTD) is a prescription for the treatment of UC in traditional Chinese medicine (TCM), which contains five herbs: *Indigo naturalis* (Qingdai in Chinese, QD), *Lithospermum erythrorhizon* (Zicao in Chinese, ZC), *Bletilla striata* (Thunb. Ex A.Murray)Rchb.F. (Baiji in Chinese, BJ), *Corydalis* Rhizoma (Yuanhu in Chinese, YH), and *Polygoni cuspidati* Rhizoma Et Radix (Huzhang in Chinese, HZ). Clinical studies have shown that in addition to improving colonic inflammation and reducing abdominal pain, QZZTD can improve mucosal erosion, bleeding, or ulcers that occur because of an ulcerated mucosa caused by intestinal aseptic vasculitis (Li et al., 2010a; Li et al., 2010b; Hou et al., 2012). Clinical trials have shown significant efficacy of *Indigo naturalis* in UC (Naganuma et al., 2020; Sun et al., 2021). A mouse experiment showed that the root of *Lithospermum erythrorhizon* has demonstrated its ability to attenuate UC (Andujar et al., 2013). Network pharmacology is a new method that can determine how TCM works through pharmacokinetic evaluation, allowing us to study its molecular mechanisms. Molecular docking is a theoretical simulation method to reveal the interaction between receptors and drug molecules and predict their affinity. In this study, we hope to investigate the possible molecular mechanism of QZZTD in the treatment of UC through network pharmacology and molecular docking, providing a new method for clinical treatment.

## 2 Materials and Methods

### 2.1 Network Pharmacology Analysis

#### 2.1.1 Collection of Main Components and Disease Targets of Qingzi Zhitong Decoction

All chemical ingredients and their related targets of each herb of QZZTD were collected from the Traditional Chinese Medicine Systems Pharmacology Database and Analysis Platform (TCMSP).^1^ Ingredients were screened conditional on oral availability (OB) ≥ 30% and drug-like (DL) ≥ 0.18. “Ulcerative colitis” was searched as the search term in the GeneCards database^2^ and the Online Mendelian Inheritance in Man (OMIM) database^3^ for disease-related target genes.

#### 2.1.2 Obtain Intersection Target Genes and Draw a Venn Diagram

Validated human species–associated target genes were selected in the Uniprot database,^4^ and gene names were obtained with R language Perl scripts. The drug-related genes were intersected with disease-related genes with a Perl script, and an intersection Venn diagram was drawn. Finally, potential targets of QZZTD for UC treatment were obtained.

#### 2.1.3 Construction of a Herb–Ingredient–Target Network for Qingzi Zhitong Decoction

Drug components were numbered and paired with their corresponding target genes. In the same way, the components and drugs were paired together to build a network.xlsx file. Each component, target gene, and drug were typed. As an example, “drug1” for Baiji, “drug2” for Huang, “gene” for the target genes, and so on were defined to create a type.xlsx file. The network of the HIT visualization of QZZTD was generated by importing the two files above into Cytoscape 3.6.1. The top four active ingredients were selected as key ingredients based on the degree. Degree indicated the number of edges between a single node and other nodes in a network.

#### 2.1.4 Build Protein–Protein Interaction Network

The intersection target genes were imported into the STRING database,^5^ and a protein interaction relationship file was obtained and imported into Cytoscape to map the PPI network of drugs for the treatment of UC by network analysis. According to the degree, the barplot of the top 30 genes was plotted with R language Perl scripts.

#### 2.1.5 Gene Ontology and Kyoto Encyclopedia of Genes and Genomes Enrichment Analysis of Intersection Target Network

We installed the “colorspace,” “stringi,” “DOSE,” “clusterprofiler,” “pathview,” and “org.Hs.eg.db” packages in R software, set q value = 0.05, and ran the program to draw a bubble chart. GO and KEGG enrichment analyses of the target genes of QZZTD in the treatment of UC were performed to elucidate the possible molecular mechanism.

### 2.2 Molecular Docking of Key Ingredients to Key Targets

The top four key active compound mol2 format files were downloaded from the ZINC database,^6^ and small molecules were extracted using the Python. After removing the redundant water molecules from the protein crystal structure and adding hydrogen atoms, we batch processed them into pdbqt files with AutoDock Raccoon to complete ligand preparation. Receptor files were prepared by downloading pdb format files of nine key targets from the Protein Data Bank (PDB) database^7^ and converting them to pdbqt files using AutoDockTools. The docking pocket is a possible binding cite for ligand in the receptor. We set up a docking box large enough to cover the entire receiver so that it contains all possible docking pockets. The size of the docking box varies depending on different receptors. Bulk molecular docking was done with AutoDock Vina to predict the binding activity of the protein to the ingredient. The minimum affinity of the receptor and ligand was ranked first in the output of the Vina. The first output of Vina of each ligand was extracted and sorted by script batch. The best combination of protein and component is the receptor and ligand with the minimum affinity. It is generally believed that the smaller the affinity of the receptor to the ligand and the stronger its stability, the better will be the docking (Wen et al., 2021).

### 2.3 Experiment Verification

#### 2.3.1 Drugs and Reagents

Composition of a dose of QZZTD: *Indigo naturalis* (3 g), *Lithospermum erythrorhizon* (10 g), *Corydalis* Rhizoma (10 g), *Bletilla striata* (Thunb. Ex A.Murray)Rchb.F. (10 g), and *Polygoni cuspidati* Rhizoma Et Radix (10 g). TCM decoction pieces of QZZTD come from the pharmacy of Guang’anmen Hospital, China Academy of Chinese Medical Sciences; 2,4,6-trinitrobenzene sulfonic acid (TNBS, Sigma, cat. SLCG2384), absolute ethyl alcohol (Sinopharm Group Chemical Reagent Co. Ltd., cat. 20140506), P-PI3Kp85 antibody (Abcam, cat. ab182651), P-AKt antibody (proteintech, cat. 66444-1-Ig), and β-actin antibody (TA-09, cat. ZS) were used.

#### 2.3.2 Animals

Healthy male Sprague Dawley (SD) rats, weighing 180 ± 20 g, were provided by Beijing Huafukang Biotechnology Co., Ltd, and the certificate number is SCXK (Beijing) 2019-0008. The rats were housed in the animal room of Guang'anmen Hospital, China Academy of Chinese Medical Sciences. The animal experiments were approved by the Institutional Animal Care and Use Committee of Guang'anmen Hospital, China Academy of Chinese Medical Sciences (No. IACUC-GAMH-2020-011, approved at 2020-12-4).

#### 2.3.3 Preparation of Qingzi Zhitong Decoction

The extract was prepared by boiling the herb samples in 10 times amount of water for 30 min. The procedure was repeated two times.

#### 2.3.4 Groups and the Construction of the Ulcerative Colitis Model

SD rats were accepted to the laboratory for 3 days before the experiments. According to the random number table, the rats were divided into three groups of six rats each: control, model, and QZZTD. Except for the control group, the UC model was prepared with TNBS. TNBS and ethanol are mixed 1:1 in the enema solution. The model rats were given enema with 50 mg/kg every other day, 2 times in total, and the control group was given enema with the same dose of saline.

#### 2.3.5 Drug Treatment

The QZZTD group was given 2 ml 8.6 g/kg QZZTD for 10 days. The other two groups were given the same dose of saline.

#### 2.3.6 Colon Damage Assessment

Colonic adhesions, ulcers, inflammation, and congestion are observed and scored. The scoring parameters are listed as follows: 1) adhesions [0, no adhesions to surrounding tissue(s); 1, minor adhesions (colon can be separated from other tissues with little effort); 2, major adhesions]; 2) ulceration [(0, normal appearance; 1, focal hyperemia, no ulcers; 2, ulceration without hyperemia or bowel wall thickening; 3, ulceration with inflammation at one site; 4, ≥two sites of ulceration and inflammation; 5, major sites of damage extending >1 cm along the length of the colon; and 6–10, damage extended to >2 cm along the length of the colon, increase the score by one for each additional cm of damage)] (Butzner et al., 1996).

#### 2.3.7 Histopathological Analysis

We collected colon tissues, fixed them in 4% paraformaldehyde overnight, dehydrated them in gradients of ethanol, and then embedded them in paraffin. Hematoxylin and eosin (HE) were used to stain the sections of tissue at 4 μm thickness.

#### 2.3.8 Enzyme-Linked Immunosorbent Assay

In order to test the anti-inflammatory effects of QZZTD on UC, colon homogenate supernatants were collected, and the expression of TNF-α was measured by ELISA kits (Neobioscience, Shenzhen, China), according to the manufacturer’s instructions.

#### 2.3.9 Western Blotting Analysis

The total protein of colon tissue was extracted using RIPA lysis buffer with the protease inhibitor or phosphatase inhibitors (Roche, China), and protein concentration was measured using the BCA protein assay kit (Cwbiotech), according to the manufacturer’s instructions. Equivalent amounts of protein (50 μg) were separated by SDS-PAGE and then transferred onto 0.45 μm PVDF membranes (Millipore, Billeria, MA, United States). After blocking with 5% non-fat milk in the TBST buffer for 1 h at room temperature, the membranes were incubated with primary antibodies at 4°C overnight. Then membranes were washed with TBST three times and incubated with secondary antibodies for 1 h at room temperature, and proteins were detected using the ECL reagent (Millipore, United States). The antibodies used were as follows: P-PI3K (1:1,000), p-AKt (1:1,000), and β-actin (1:1,000).

#### 2.3.10 Statistical Analysis

The experimental data were analyzed using SPSS 22.0 statistical software (IBM Corp., Armonk, NY, United States). The figures were obtained by GraphPad Prism 7.0 software (GraphPad Software Inc., San Diego, CA, United States). All results were expressed as mean ± standard error of the mean (SEM). Multigroup comparisons were performed using one-way analysis of variance (ANOVA) and the post hoc statistical least significant difference (LSD) test. Tamhane’s T2 test is used when the variance is uneven. A *p* value < .05 was considered statistically significant.

## 3 Results

### 3.1 Network Pharmacology Analysis

#### 3.1.1 Predictive Results of Targets for Qingzi Zhitong Decoction in Ulcerative Colitis

**FIGURE 1:**
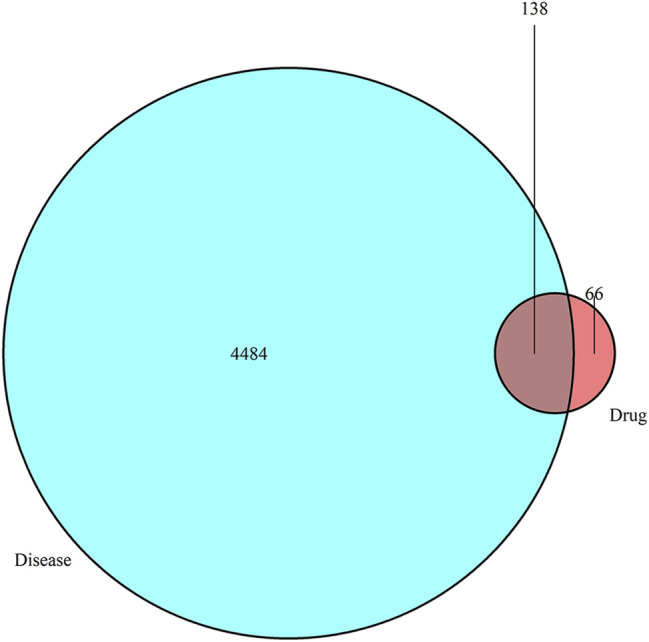
Venn diagram showing that drugs have 138 common targets with diseases that can be used as potential therapeutic targets.

A total of nine ingredients of QD, 12 ingredients of ZC, nine ingredients of BJ, 49 ingredients of YH, and 10 ingredients of HZ were collected from the TCMSP database according to OB ≥ 30% and DL ≥ 0.18. Among them, HZ and YH, HZ and QD, and YH and ZC each have one common ingredient, while three or more drugs have no common ingredient. In total, 204 drug ingredient–related targets (not repeated) were obtained from the Uniprot database, and 4,622 disease-related targets were obtained from the GeneCards and OMIM databases. The intersection of the drug target and the disease target yielded 138 potential targets, as shown in the Venn diagram (Figure 1).

#### 3.1.2 Result of Network Construction

**FIGURE 2:**
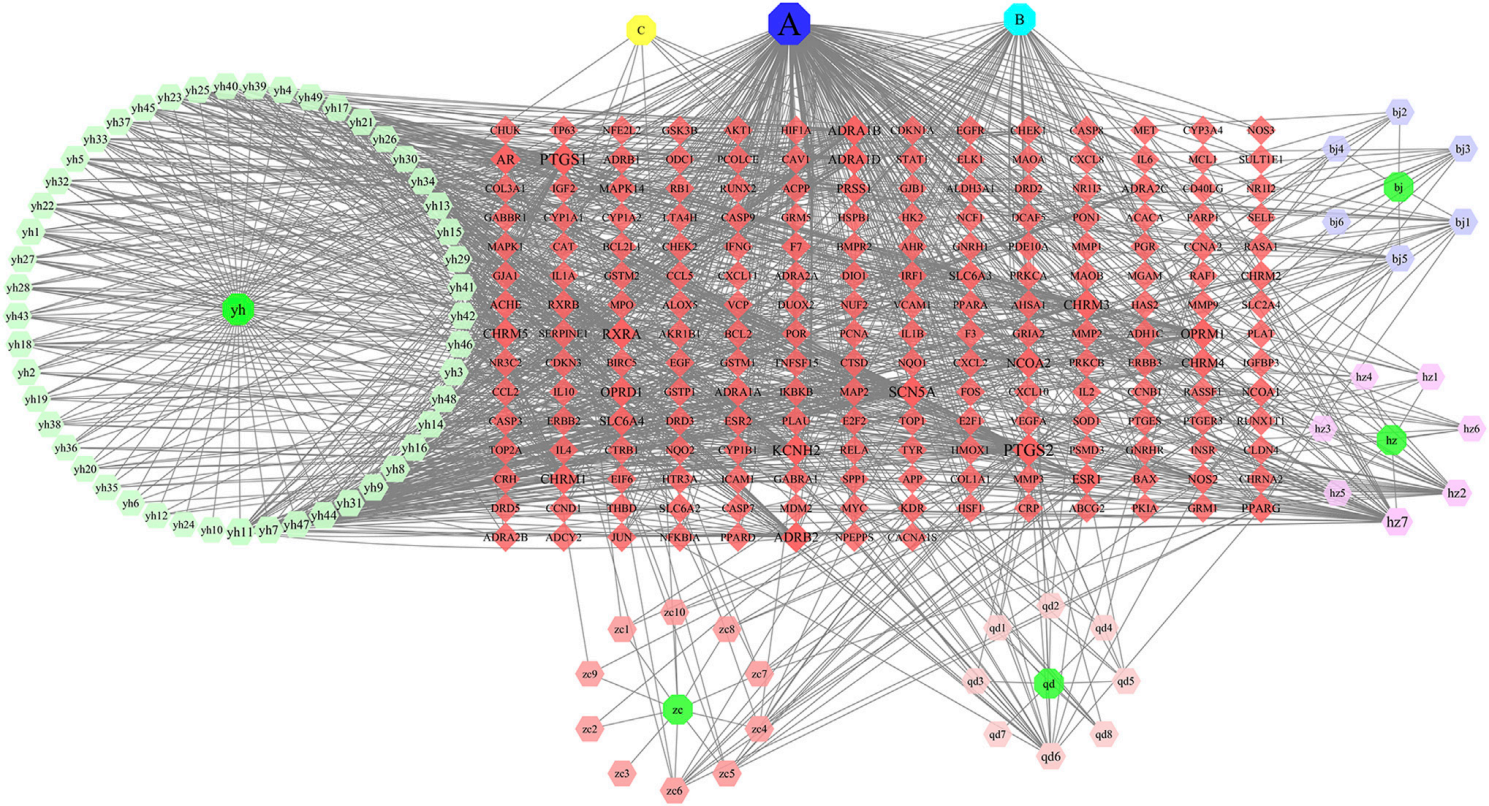
In the network, QD represents *Indigo naturalis*, ZC represents *Lithospermum erythrorhizon*, BJ represents *Bletilla striata* (Thunb. Ex A.Murray)Rchb.F., YH represents *Corydalis* Rhizoma, and HZ represents *Polygoni cuspidati* Rhizoma Et Radix. **(A)** Common component of HZ and YH. **(B)** Common component of HZ and QD. **(C)** Common component of YH and ZC. The node size and node label size are determined by the node degree value. The higher the degree value, the larger will be the node and label.

**FIGURE 3:**
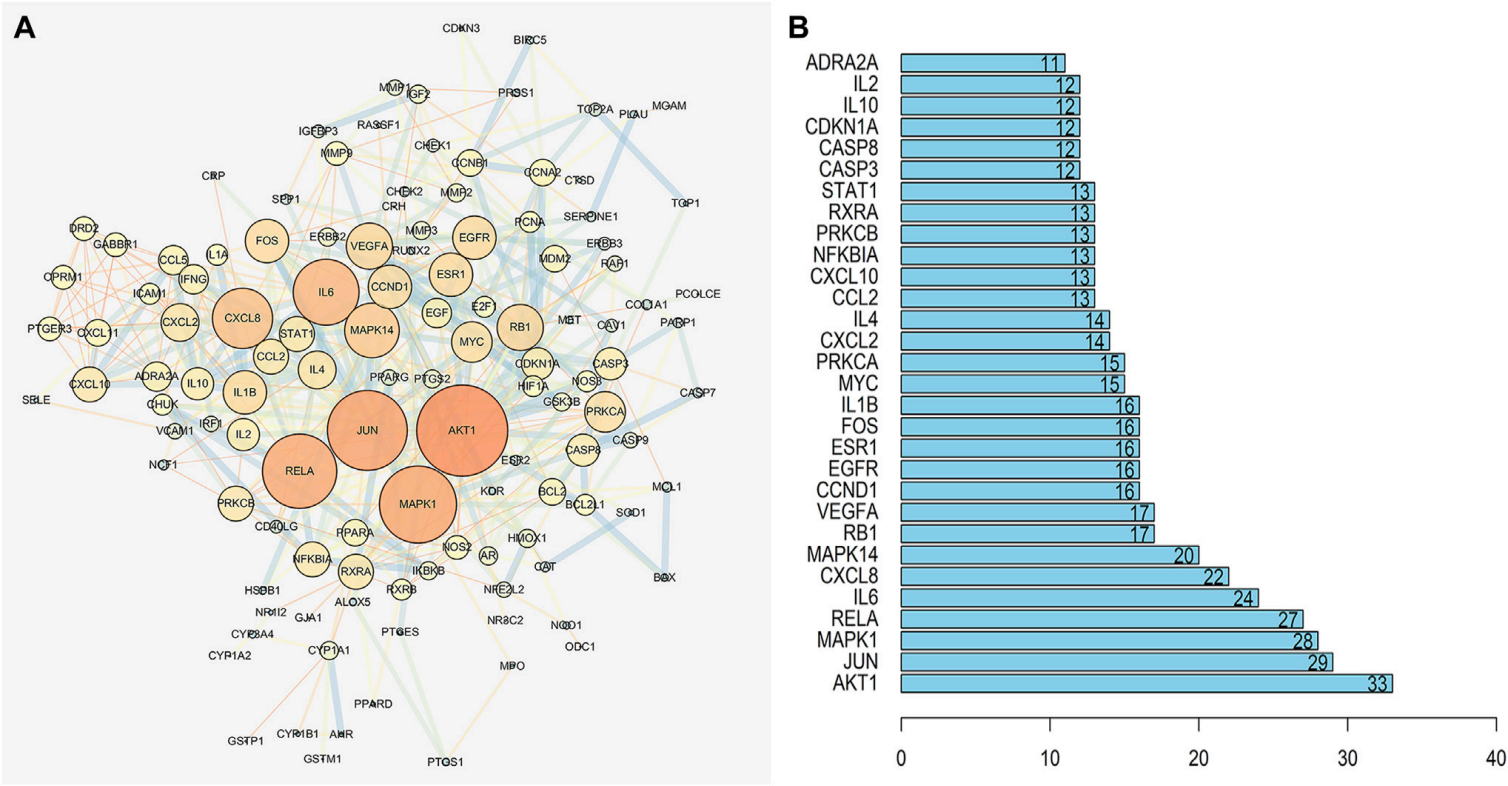
**(A)** PPI network of QZZTD against UC. The darker the nodes in the figure, the more intense will be the interaction between the target proteins. **(B)** It shows the top 30 possible targets of PPI, and we screened the top nine key proteins from the barplot.

The network diagram of herb, ingredients, and intersection targets constructed by Cytoscape 3.6.1 consists of 292 nodes and 1,363 edges (Figure 2). A total of four key active ingredients were selected by the HIT network, which were quercetin, luteolin, hyndarin, and beta-sitosterol (Table 1). We imported intersection target genes into the STRING database, setting “minimum interaction score = 0.9” and “hide disconnected nodes.” In the PPI network constructed with Cytoscape 3.6.1, there are 138 nodes and 488 edges. The larger and darker the nodes in the PPI network, there will be more interactions between the node protein and surrounding proteins (Figure 3A). We used the script to make the barplot of the top 30 measures (Figure 3B). Both the barplot and PPI network showed that the main proteins involved were Akt serine/threonine kinase 1 (Akt1), jun proto-oncogene (JUN), mitogen-activated protein kinase 1 (MAPK1), rela proto-oncogene (RELA), interleukin 6 (IL-6), c-x-c motif chemokine ligand 8 (CXCL8), mitogen-activated protein kinase 14 (MAPK14), rb transcriptional corepressor 1 (RB1), and vascular endothelial growth factor-A (VEGFA).

**TABLE 1:** Key active ingredients.

Number	Mol ID	Molecule name	Molecule weight	Structure^8^	OB (%)	DL
1	MOL000098	Quercetin	302.25	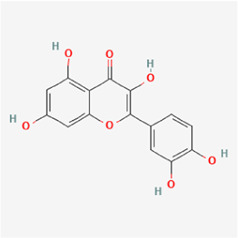	46.43	0.28
2	MOL000006	Luteolin	286.25	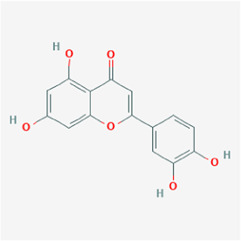	36.16	0.25
3	MOL004071	Hyndarin	355.47	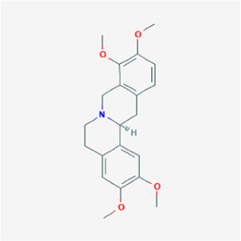	73.94	0.64
4	MOL000358	Beta-sitosterol	414.79	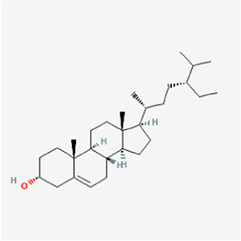	36.91	0.75

#### 3.1.3 Results of Gene Ontology and Kyoto Encyclopedia of Genes and Genomes Enrichment Analysis

**FIGURE 4:**
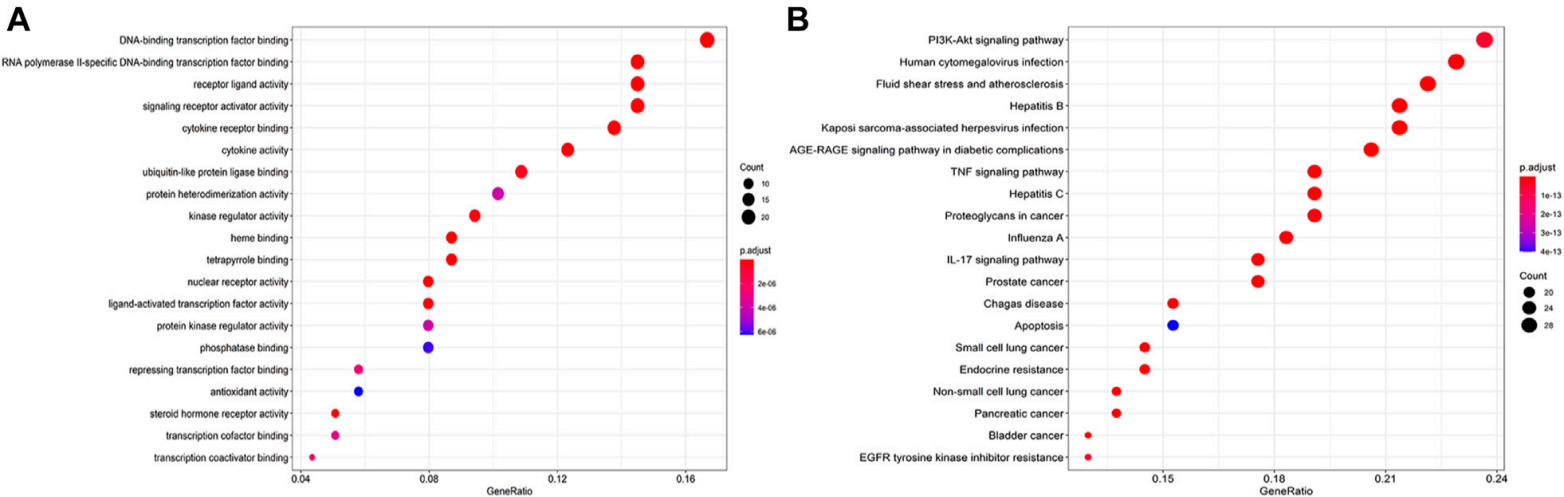
**(A)** Top 20 biological processes for the GO analysis. The results show that it involves a variety of biological processes, such as DNA-binding transcription factor binding, RNA polymerization, and receptor–ligand activity. **(B)** Top 20 pathways for the KEGG analysis. PI3K-Akt, AGE-RAGE, TNF, and IL-17 signaling pathways were intervened in the treatment of UC.

The GO enrichment analysis predicted 144 biological processes, with the top three mainly involving DNA-binding transcription factor binding, RNA polymerization, and receptor–ligand activity (Figure 4A). It means that the process of QZZTD in the treatment of UC is very complex, and the main biological functions may be gene transcription and protein translation as well as signal transduction. The KEGG enrichment produced 153 pathways. Most of the enriched top 20 pathways were not reported to be associated with UC, such as human cytomegalovirus infection, fluid shear stress, and atherosclerosis. Finally, four main pathways were involved in the treatment of UC by QZZTD: the phosphatidylinositol 3-kinase (PI3K)-Akt signaling pathway, the advanced glycosylation end product (AGE)-RAGE signaling pathway, tumor necrosis factor (TNF) signaling pathway, and IL-17 signaling pathway (Figure 4B).

### 3.2 Molecular Docking Analysis

To further validate the molecular mechanism of QZZTD intervention in UC, molecular docking was performed using key active compounds as ligands and key target proteins as receptors. Docking results showed that the key active ingredients of QZZTD had good binding activity against key targets, except RELA, and the Vina affinity was better than 5-aminosalicylic acid. 5-aminosalicylic acid is an active comparator for the treatment of UC (Cevallos et al., 2021; He et al., 2021) (Table 2)^8^.

**TABLE 2:** Vina affinity of ingredient–target docking.

Compound	Affinity (Kcal mol^−1^)								
	Akt1	CXCL8	IL-6	JUN	MAPK1	MAPK14	RB1	RELA	VEGFA
Quercetin	−10.3	−7.1	−7.3	−7.1	−9.8	−8.8	−9	−4.7	−7.5
Luteolin	−11.3	−7.6	−7.7	−6.9	−9.4	−8.9	−8.4	−4.3	−8.7
Hyndarin	−9.8	−6.1	−6.3	−6.1	−8	−7.6	−7.7	−3.6	−7.6
Beta-sitosterol	−11.8	−6.8	−7.5	−7.1	−8.2	−9	−9.1	−4.6	−8.4
5-Aminosalicylic acid	−6	−4.5	−4.9	−4.4	−5.5	−5.7	−5.5	−3.2	−5.7

### 3.3 Experiment Verification

#### 3.3.1 Scoring of Colonic Damage

**FIGURE 5:**
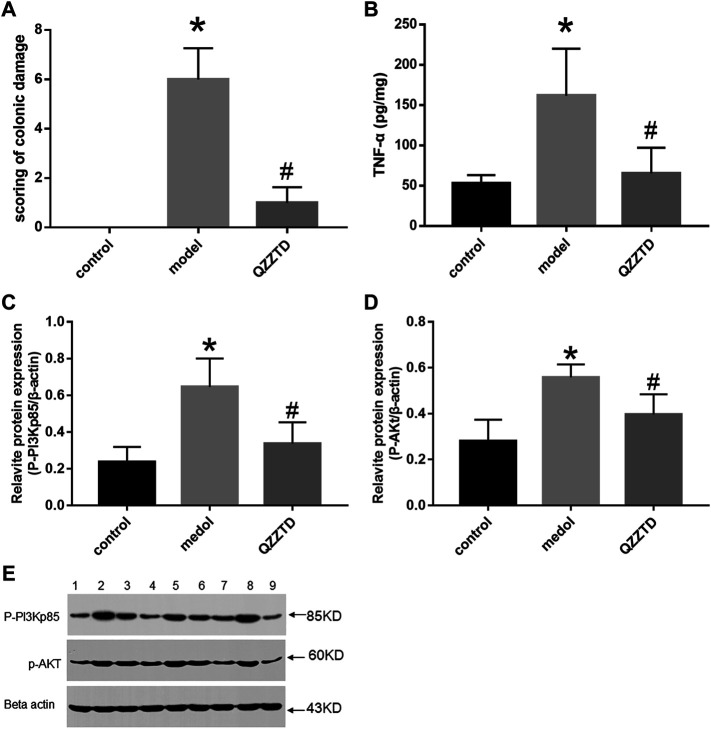
Intervention effect of QZZTD on scoring of colonic damage **(A)**. Pro-inflammatory cytokine TNF-α **(B)**. Relative expression levels of p-pi3kp85 **(C)** and p-Akt **(D)**. Proteins and their bands **(E)**. We utilized the Gel Image system ver.4.00 (Tanon, China) for band grayscale analysis. Lanes 1, 4, and 7 represent the control group, lanes 2, 5, and 8 represent the model group, and lanes 3, 6, and 9 represent the QZZTD group.

As compared to the control group, the score of colonic damage in the model group increased significantly; however, as compared to the model group, the score of the QZZTD group decreased significantly. The results showed that TNBS aggravated the colon damage in rats, while QZZTD alleviated it (Figure 5A).

#### 3.3.2 Effects of Qingzi Zhitong Decoction on Histopathological Changes in Colon Tissues

**FIGURE 6:**
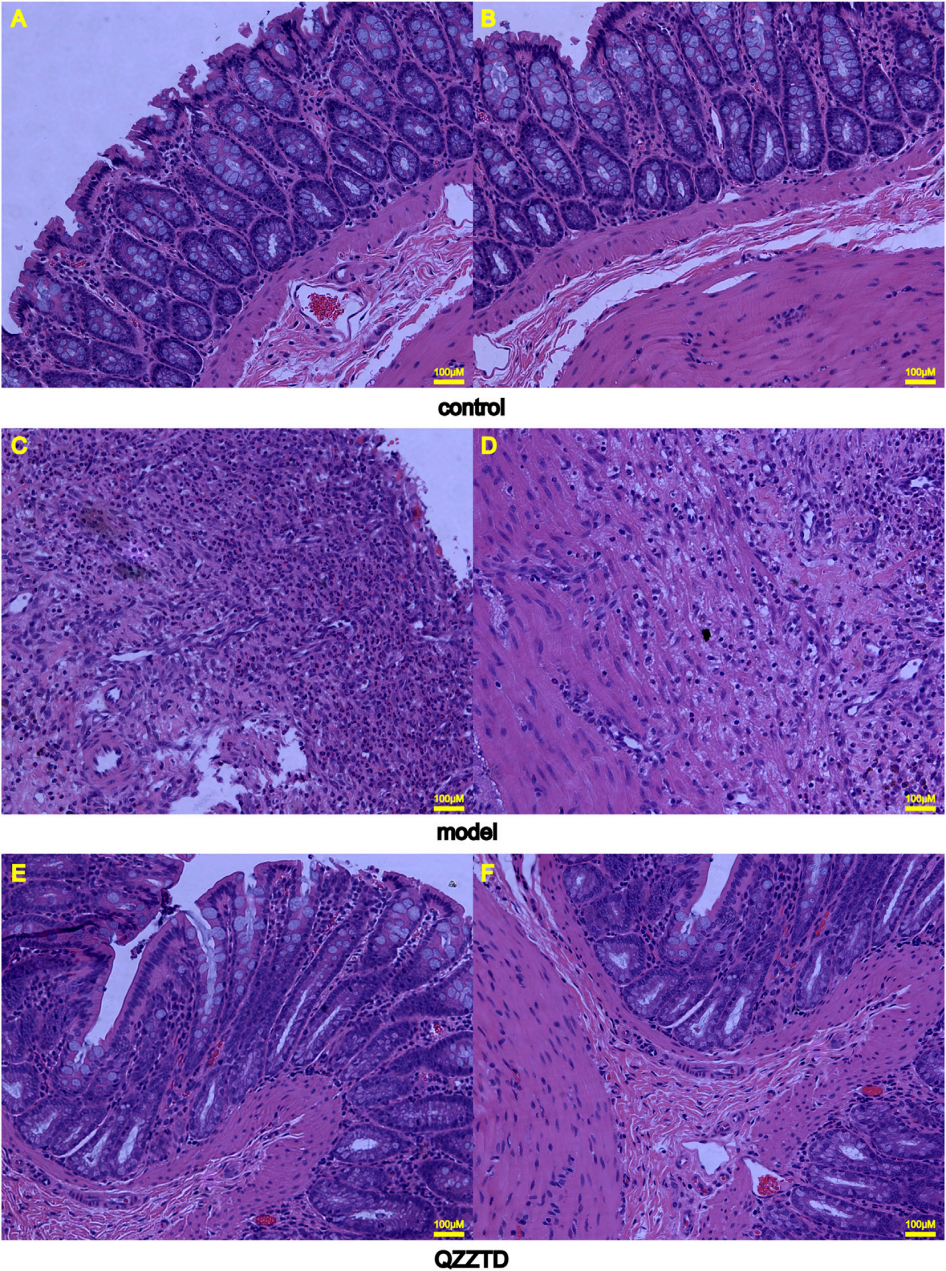
Representative images of colonic tissues with HE staining (×200 magnification). **(A)** Colonic mucosal layer in the control group. **(B)** Submucosa and muscle layers in the control group. **(C)** Colonic mucosal layer in the model group. **(D)** Submucosa and muscle layers in the model group. **(E)** Colonic mucosal layer in the QZZTD group. **(F)** Submucosa and muscle layers in the QZZTD group.

HE staining was applied to observe the pathological changes of colon tissues. Those in the control group had goblet cells and absorptive cells in the mucosa, but there were no obvious abnormalities in the submucosa and muscle layers (Figures 6A,B). In the model group, the mucosa was necrotic, with many inflammatory cells infiltrating the necrotic part, and macrophages and lymphocytes were seen in the submucosa, while there were no obvious abnormalities in the muscle layer (Figures 6C,D). The mucosal layer of the QZZTD group showed small amounts of inflammatory cells and red blood cells, and the number of goblet cells was lower than in the control group. Vascular hyperplasia, macrophages, and inflammatory cells were seen in the submucosa; the number of macrophages and inflammatory cells was reduced compared with the model group, and no significant abnormalities were seen in the muscle layer (Figures 6E,F).

#### 3.3.3 Qingzi Zhitong Decoction Treatment Inhibited the Expression of TNF-α in Colon Tissues

TNF-α is an important pro-inflammatory factor in UC. We investigated whether QZZTD possesses the anti-inflammatory effect in UC. An ELISA essay was performed in colonic samples. TNBS significantly increased the expression of TNF-α in the model group, and QZZTD significantly reduced the expression of TNF-α compared to the model group (Figure 5B). The results show that QZZTD treatment inhibited the inflammatory response induced by TNBS in UC.

#### 3.3.4 QZZTD Regulates PI3K/Akt Signaling Pathway to Ameliorate Ulcerative Colitis

We investigated whether QZZTD could exert therapeutic effects on UC by inhibiting inflammation *via* regulating the expression of *p*-PI3Kp85, *p*-AKt. The protein expression of p-PI3Kp85 and p-AKt in the model group was significantly higher than that in the control group. QZZTD significantly reduced the protein expression of p-PI3Kp85, p-AKt compared to the model group (Figures 5C–E). These data suggest that TNBS activates the PI3K/Akt signaling pathway, and QZZTD treatment can inhibit the expression of protein of the pathway and ameliorate UC.

## 4 Discussion

### 4.1 Qingzi Zhitong Decoction Regulates the PI3K-Akt Pathway to Alleviate Ulcerative Colitis

We used network pharmacology and molecular docking to predict the molecular mechanism of QZZTD for the treatment of UC. Similarly, some studies have used network pharmacology combined with molecular docking to predict the efficacy and mechanism of drug action in UC, suggesting that potential mechanisms of drug action on the disease can be explored in this way. In a study composed of Huai Hua San formula, the investigators used this approach and validated it *in vitro*, discovering the binding properties of three key compounds (quercetin, luteolin, and nobiletin) to three key proteins (JUN, tumor protein P53, and estrogen receptor 1) and revealing the mechanism of quercetin affecting the protein expression of c-jun and the PI3K/Akt pathway to improve UC (Liu J. et al., 2021). Another study also used this approach and combined it with animal studies to screen for key components (puerarin, baicalein, berberine, and glabridin) and key pathways (hypoxia inducible factor 1, PI3K-AKT, and TNF). It revealed that Gegen Qinlian Decoction ameliorates inflammation *via* downregulating the epidermal growth factor receptor (EGFR)/PI3K/AKT signaling pathway and pro-inflammatory cytokines (Liu X. et al., 2021). Studies using network pharmacology combined with *in vivo* and *ex vivo* experiments and RNA-sequencing methods have similarly confirmed that genes downregulated by drugs were enriched in inflammation-related pathways (Yu et al., 2021). Additionally, there are research studies that use the same approach to study drugs for different diseases, such as resveratrol, which can alleviate COVID-19–related inflammation (Xiao et al., 2021). In the study of patchouli alcohol for gastric cancer, network pharmacology and *in vitro* experiments revealed that the drug acts mainly *via* PI3K/AKT and MAPK signaling pathways (Song et al., 2021). However, some studies were conducted only on single drugs, such as those on Curcuma, Puerariae Radix, Isopsoralen, and Paeoniflorin (Chen et al., 2021; Han et al., 2021; Liu S. et al., 2021; Wang et al., 2021). Synthetic drugs including mesalazine and azathioprine, and TNF-α blocker such as infliximab have therapeutic effects on UC, but they also have side effects of inducing pancreatitis, cardiomyopathy, systemic reactions, and sweet syndrome (El-Azhary et al., 2008; Mourad et al., 2015; Correia et al., 2021). In contrast, no significant adverse effects were found in the clinical use of the herbal compounds (Hou et al., 2012). These studies support the feasibility of our study protocol. Compared to single drug studies, compounding studies may reveal synergistic mechanisms of drug interactions and better safety of TCM preparations compared to chemically synthesized drugs.

The PI3K-AKt pathway plays a critical role in the regulation of the inflammatory response during UC progression. The abnormal activation of the PI3K/AKt signaling pathway in UC can have a significant effect on the expression and secretion of pro-inflammatory cytokines. These cytokines are responsible for the development of UC (Dai et al., 2013; Liu X. et al., 2021). We hypothesized that QZZTD may reduce the expression of inflammatory factors by regulating the PI3K-AKt pathway. Our experiments verified that QZZTD intervention reduced colon damage scores and pathological changes. Furthermore, QZZTD inhibited PI3K-AKt pathway activation, reduced inflammation, and alleviated UC. The aforementioned study also yielded the same results, revealing that TCM treatment of UC may mainly produce efficacy by regulating the PI3K-Akt signaling pathway (Liu J. et al., 2021; Liu X. et al., 2021; Song et al., 2021; Yu et al., 2021).

Our KEGG analysis included the AGE–RAGE signaling pathway in addition to the pathway of PI3K-Akt, TNF, and IL-17 mentioned in the aforementioned study. Previous studies have found that the activation of the AGE/RAGE signaling pathway can activate the PI3K-Akt signaling pathway and the mitogen-activated protein kinase (MAPK) signaling pathway to activate inflammation, thus the regulation of this pathway may be beneficial for the treatment of UC and merits further exploration (Grille et al., 2003; Perkins, 2007).

### 4.2 Interaction Between Inflammatory Cells and Inflammatory Factors in Ulcerative Colitis

**FIGURE 7:**
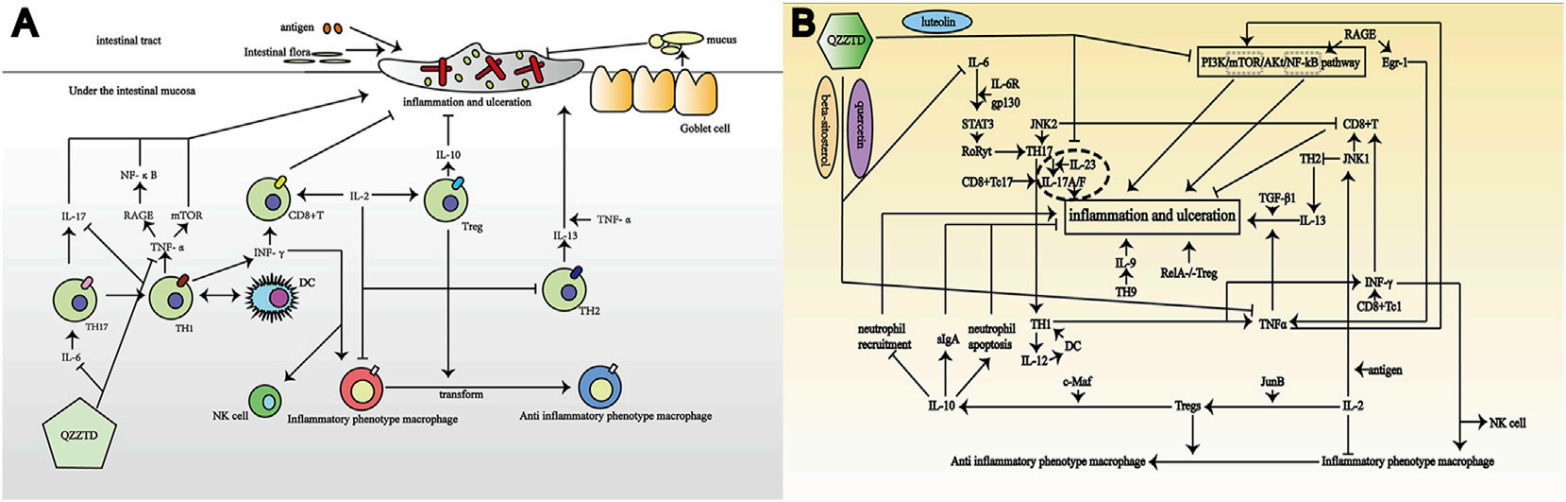
Pathological mechanism of UC and potential mechanism of QZZTD treating UC. In the figures, “→” represents the promoting effect and “┤” represents the inhibiting effect. **(A)** Combination of antigens, intestinal bacteria, intestinal mucosal goblet cells, T cells, and inflammatory factors contribute to UC. **(B)** T cells and inflammatory factors promote or inhibit each other to affect the formation of UC, which QZZTD can potentially regulate through PI3K-Akt, AGE-RAGE, TNF, and IL-17 signaling pathways.

In general, UC is considered a spontaneous immune disorder. Moreover, it is affected by intestinal microorganisms, which cause inflammation, and by epithelial barrier dysfunction. In the human gut, food introduces antigens, which in turn trigger inflammatory and immune responses. The intestine reduces injury by enhancing mucus secretion by intestinal goblet cells, increasing secretory immunoglobulin-associated alpha (IgA) by B cells, reducing the maturation of dendritic cells (DCs), increasing the number of Foxp3+ regulatory T cells (Tregs), and enhancing the colonic function. The inflammatory response is mainly caused by the interaction between T cells and inflammatory factors. T cells are divided into multiple subtypes, of which CD4+T cells can differentiate into T helper 17 (TH17), TH9, TH22, and Treg cells producing the hallmark cytokine transforming growth factor-β (TGF-β). Similarly, CD8^+^ T cells can differentiate into interferon (IFN)-γ–producing Tc1, Tc2, and Tc17 and regulatory CD8^+^ T cells (Shen and Shi, 2019). Another recognized classification divides T cells into TH1 and TH2. TH1 promotes inflammation, and its specific production of IL-12 activates DCs, which in turn stimulate Th1 differentiation to produce TNF-α and IFN-γ for activating macrophages, natural killer (NK) cells, and CD8^+^ T cells. TH2 secretes IL-13, which is involved in the destruction of the intestinal mucosal barrier with TGF-β1 (Heller et al., 2005). In addition, Th9 cells can interfere with the barrier function of intestinal epithelium by affecting cell proliferation and tight junction molecules (Weigmann and Neurath, 2017). There are two aspects of immune regulation in UC: pro-inflammatory regulation and anti-inflammatory regulation. And we briefly summarized the interaction between T cells and inflammatory factors in UC and the potential intervention approaches of QZZTD (Figure 7).

#### 4.2.1 Pro-Inflammatory Regulation: Pro-Inflammatory T Cells and Related Cytokines

TNF-α and TH17 are the most important factors in the pathogenesis of UC. Studies have shown that TNF-α synergizes with IL-13 to perturb the epithelial barrier function by regulating the expression of genes involved in tight junction formation, which is a trigger for intestinal epithelial cell death (Heller et al., 2005; Meylan et al., 2011; Nielsen, 2014; Jung et al., 2019). However, etanercept (an antagonist of TNF-α) markedly reduced the levels of colonic inflammation (Popivanova et al., 2008). The Th17-derived Th1 cells responsible for several autoimmune diseases, including UC, are thought to provide the major pathogenic effector population for the diseases (Rampal et al., 2021; Bhaumik and Basu, 2017). The IL-6 can cause activation of signal transducer and activator of transcription (STAT)3, which induces IL-17 (including IL-17A and IL-17F) *via* activation of retinoic acid–related orphan receptor gamma t (RORγt) (Bhaumik and Basu, 2017). And IL-17A is involved in local chronic inflammation inducing pro-inflammatory cytokine expression, leading to mucosal destruction and altering mucosal healing (Catana et al., 2015).

There are multiple pathways for T-cell activation in intestinal inflammation. The absence of c-Jun N-terminal kinase (JNK)2 results in IL-2 production and CD8^+^ T-cell proliferation, whereas JNK1-deficient CD8^+^ T cells are unable to undergo antigen-stimulated expansion *in vitro*, even in the presence of exogenous IL-2 (Conze et al., 2002). JNK2 is required for IFN-γ production by peripheral CD4^+^T cells and subsequent Th1 differentiation *in vitro*, whereas JNK1 appears to be a negative regulator of Th2 differentiation *in vitro* (Dong et al., 1998; Yang et al., 1998). Mitogen-activated protein kinase 2 (MAP3K2) can modulate intestinal CD4^+^T-cell–mediated cellular immunity *via* the IL-18–MAP3K2–JNK axis, which causes intestinal inflammation (Wu et al., 2021). Qiao’s study has reported that there is a positive feedback loop between pro-inflammatory cytokines (IL-1β, IL-6, and TNF-α) and the PI3K/mechanistic target of rapamycin (mTOR)/Akt/nuclear factor NF-kappa B (NF-κB) signaling (Qiao et al., 2020). On the other hand, activation of mTOR signaling can cause colitis, and overproduction of IL-6 can inhibit Treg proliferation (Fujimoto et al., 2011; Sun et al., 2012). Alternatively, it has been shown that RAGE stimulation upregulates two key transcription factors implicated in inflammatory responses, NF-κB and early growth response-1 (Egr-1) that contributes to tumor necrosis factor-α expression (Schmidt et al., 1995; McMullen et al., 2005; Chang et al., 2008). The aforementioned inflammatory responses mediated by inflammatory factors and T cells are one of the mechanisms in UC.

#### 4.2.2 Immune Suppression: Tregs and Related Cytokines

Treg cells are considered an attractive target for the treatment of immunological and inflammatory pathologies, which can actively suppress the immune function (Vinolo et al., 2011). There are several molecules known to regulate Treg-dependent intestinal homeostasis, but IL-10 is the most important and common among them (Kuhn et al., 1993; Sellon et al., 1998; Rubtsov et al., 2008; Josefowicz et al., 2012). A study has shown that specific ablation of Treg is sufficient to induce spontaneous colitis (Rubtsov et al., 2008). Tregs can secret anti-inflammatory cytokines and regulate the recruitment/activation of other immune cells to dampen inflammation (Li et al., 2018). For instance, Tregs suppress neutrophil recruitment directly *via* IL-10 and induce neutrophil apoptosis indirectly *via* TGF-β. In studying the effect of c-Maf on Treg cells, it was found that the expression of IL-10 in Treg cells is c-Maf–dependent, which subsequently promotes the secretion of intestinal sIgA, protects the intestinal mucosa, and ameliorates UC (Neumann et al., 2019). Timosaponin AIII and its metabolite sarsapogenin have been found to inhibit intestinal inflammation by potently inhibiting NF-κB and MAPK activation, as well as interleukin-1 receptor–associated kinases (IRAK1), transforming growth factor beta–activated kinase 1 (TAK1) and NF-κB inhibitor alpha phosphorylation in lipopolysaccharide(LPS)-stimulated macrophages, and by reducing NF-κB activation and the levels of interleukin IL-1β, TNF-α, and IL-6, while simultaneously increasing the production of IL-10 (Lim et al., 2015). The mice that specifically knock out RELA in Tregs will eventually suffer from severe autoimmune syndrome, including UC. In addition, Tregs can also promote the polarization of macrophages from an inflammatory phenotype to an anti-inflammatory phenotype (Lui et al., 2020). Restoring the balance between Th17 and Tregs can reduce colitis (Liu et al., 2020).

IL-2 is expressed during T-cell activation and induces the proliferation and differentiation of T cells, which plays a key role in the development of T cells. IL-2 promoted Treg cell development *via* AP-1 transcription factor JunB, and injection of IL-2–anti-IL-2 antibody complex in JunB^fl/fl^ Cd4-Cre mice expanded Treg cells and alleviated DSS-induced colitis (Katagiri et al., 2019). T cells stimulated by IL-2 showed increased expression of phosphorylated signal transducer and activator of transcription 5 (pSTAT5) and promoted Treg cells proliferation, and IL-2 treatment caused massive eosinophil accumulation and activation in the inflamed colon, which alleviated UC (Abo et al., 2019; Wang et al., 2019).

The studies mentioned before indicate that the pathogenesis of UC is caused by both pro-inflammatory and anti-inflammatory effects, which is a pathological process with multiple pathways and multiple targets. The pathways and targets predicted by network pharmacology are consistent with the pathogenesis of UC described before.

### 4.3 Mechanism of the Components of Qingzi Zhitong Decoction in the Treatment of Ulcerative Colitis

UC is associated with damp-heat, blood stasis, and intestinal vascular ischemia in the TCM theory. The harmonization of drug components in QZZTD can jointly play a role in removing damp-heat and blood stasis, thereby reducing intestinal inflammation. The key ingredients of QZZTD have relevant reports for the treatment of UC: among the key ingredients obtained from the HIT network, quercetin, a natural flavonoid, regulates a variety of inflammatory factors. By treating LPS-induced ulcerative colitis mice with quercetin, intestinal inflammation is inhibited (Dicarlo et al., 2019). The intestinal inflammatory factor TNF-α and the anti-inflammatory factor lipocalin-2 (LCN-2) were upregulated after LPS stimulation, whereas quercetin inhibited their expression. Quercetin also inhibits the production of IL-6 and IL-1β in lipopolysaccharide-activated human mononuclear cells and promotes the secretion of IL-10 (Okoko and Oruambo, 2009; Seo et al., 2015). As previously discussed, TH17 is elevated in mice with UC, and quercetin can be used to restore the balance between Th17 and Treg. During inflammation, myeloperoxidase (MPO) is secreted as a result of oxidative burst, which can be reduced through quercetin treatment (Riemschneider et al., 2021). Quercetin is one of the extracts of bryophyllum pinnatum (lamarck) leaf that promotes the downregulation of toll-like receptor and Kappa B p65 nuclear factor gene expression, as well as reducing pro-inflammatory factors and oxidative mediators, and improved inflammation (Andrade et al., 2020).

Luteolin protected the intestinal epithelial barrier function by increasing the resistance values and tight junction (TJ) protein expression (Li et al., 2021). By activating extracellular signal-regulated kinase (ERK) signaling, luteolin may moderate colon tissue damage and reduce inflammation, apoptosis, and autophagy (Vukelic et al., 2020). Luteolin treatment can decrease the activation of NF-κB and production of IL-17 and IL-23, leading to reduced colonic damage in UC rats (Donder et al., 2018). Hyndarin is a compound extracted from corydalis with analgesic and antispasmodic properties, which has the highest concentration in the small intestine 1 hour after administration and effectively relieves spastic pain in UC (Xiao, 2016; Qiu et al., 2019).

β-sitosterol is one of the components of many medicinal plants, such as *Moringa oleifera*, which inhibits the production of several inflammatory mediators: TNF-α, IL-1β, IL-6, and IL-8, and reactive oxygen species (ROS), separately (Liao et al., 2018). β-sitosterol in red ginseng attenuates inflammation by modulating the MAPK/NF-κB pathway (Lee et al., 2018). *Canna x generalis* rhizome ethanol extract (CGE) contains quercetin and β-sitosterol. CGE reduces colon inflammation by inhibiting malonaldehyde (MDA), pro-inflammatory mediators (nitric oxide and MPO), and downregulating NF-ҡB expression. Additionally, CGE restored the expression of tight junction proteins (occludin and claudin-1) and mucosal barrier function (Mahmoud et al., 2021).

In addition to these key ingredients, the drugs in QZZTD exert their effects by inhibiting the activation of MAPK, NF-κB, and Jak3/STAT3 pathways and the production of ROS and NADPH oxidase 4 (NOX4), reducing nucleotide-binding oligomerization domain leucine-rich repeat and pyrin domain containing 3 (NLRP3), and downregulating pro-inflammatory factors such as IL-1α, IL-1β, IL-6, IL-8, IL-17, TNF-α, and IL-18, while increasing IL-10 (Yue et al., 2016; Sun et al., 2021). Overall, the predicted ingredients of QZZTD have the potential to reduce intestinal inflammation and modulate mucosal dysfunction resulted from intestinal dysbacteriosis, thereby could be applied to the treatment of UC.

## 5 Conclusion

In summary, we used network pharmacology and molecular docking methods to predict that QZZTD can ameliorate UC. Based on the results in docking studies, the main ingredients, such as quercetin, luteolin, hyndarin, and beta-sitosterol, are expected to bind with and regulate the function of AKT1, MAPK1, IL-6, and VEGFA, which could be important for the treatment of UC. And our experiment verified that QZZTD inhibited PI3K-AKt pathway activation, reduced inflammation, and alleviated UC. This study may provide new insights for dissecting complex mechanisms underlying the functions of TCM formulations or herbal medicines and may identify new pathways important for pathogenesis and treatments of UC.

## Data Availability

The datasets presented in this study can be found in online repositories. The names of the repository/repositories and accession number(s) can be found in the article/Supplementary Material.

## Footnotes

1 Traditional Chinese Medicine Systems Pharmacology Database and Analysis Platform. https://tcmspw.com/tcmsp.php. [Accessed November 8, 2020].

2 GeneCards database. https://www.genecards.org/. [Accessed November 16, 2020].

3 Online Mendelian Inheritance in Man database. https://omim.org/. [Accessed November 16, 2020].

4 Uniprot database. https://www.uniprot.org/. [Accessed November 16, 2020].

5 STRING database. https://www.string-db.org/. [Accessed January 6, 2021].

6 ZINC database. http://zinc.docking.org/. [Accessed February 2, 2021].

7 Protein data bank database. http://www.rcsb.org/. [Accessed February 2, 2021].

8 PubChem. https://pubchem.ncbi.nlm.nih.gov/. [Accessed August 3, 2021].
