# Supplementary figures and images for: Network Pharmacology and Molecular Docking Analysis on Molecular Mechanism of Qingzi Zhitong Decoction in the Treatment of Ulcerative Colitis
Source: Front Pharmacol. 2022 Feb 8;13:727608. doi: 10.3389/fphar.2022.727608 (PMC8883437; doi:10.3389/fphar.2022.727608)

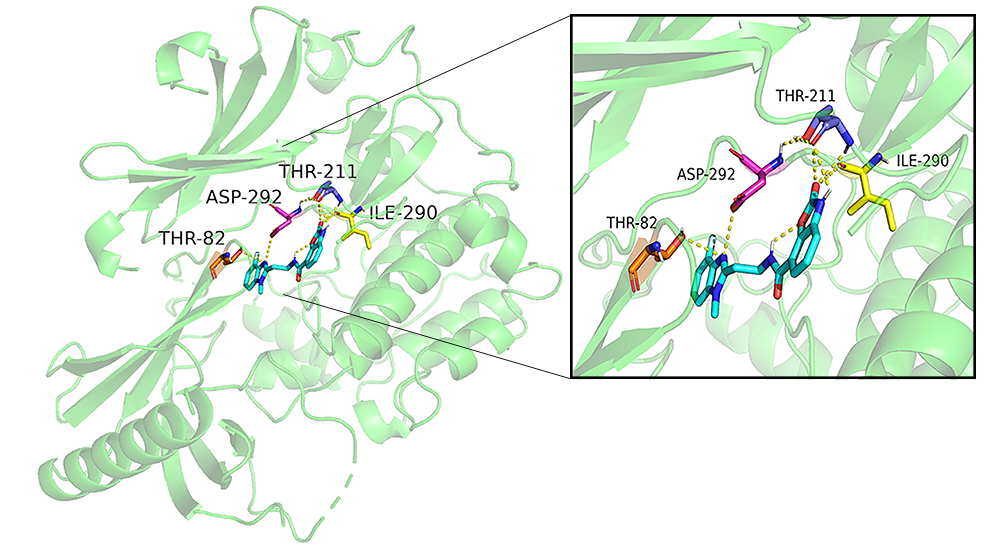

Supplement: Supplementary file 1 [file DataSheet1.ZIP › Figure 5/figure 5(A1).tif]

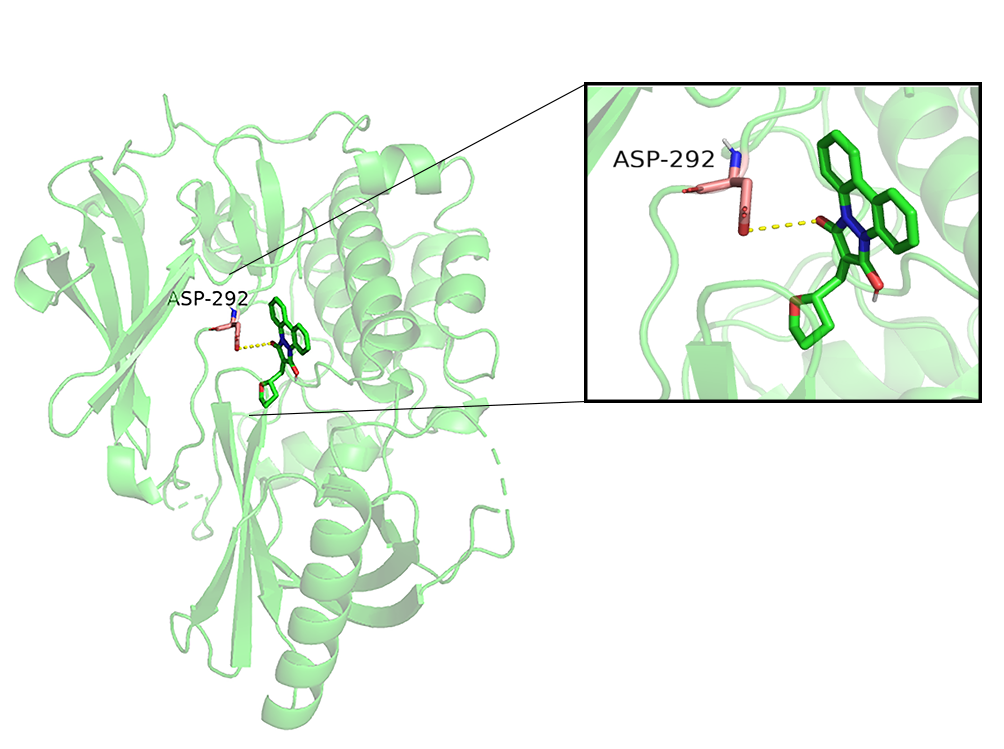

Supplement: Supplementary file 1 [file DataSheet1.ZIP › Figure 5/figure 5(A2).tif]

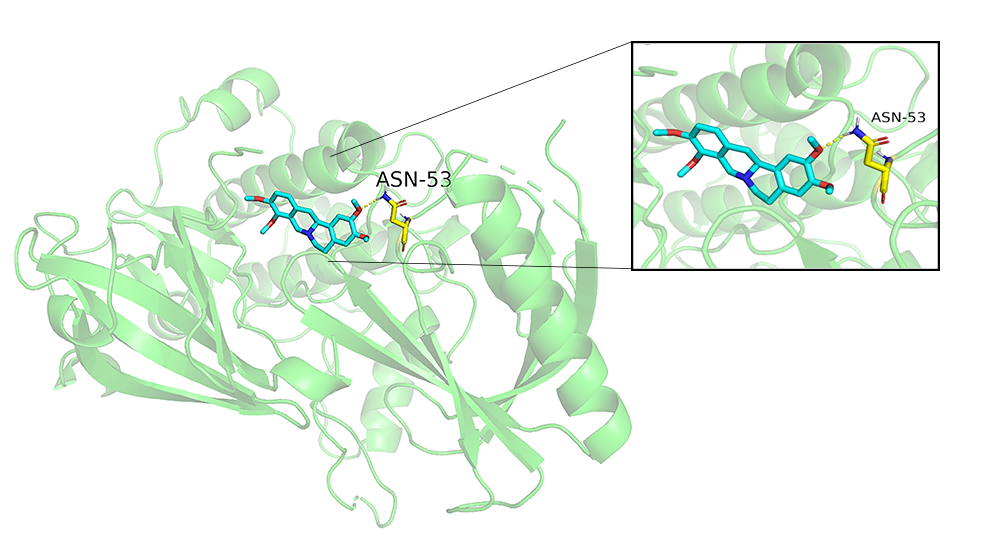

Supplement: Supplementary file 1 [file DataSheet1.ZIP › Figure 5/figure 5(A3).tif]

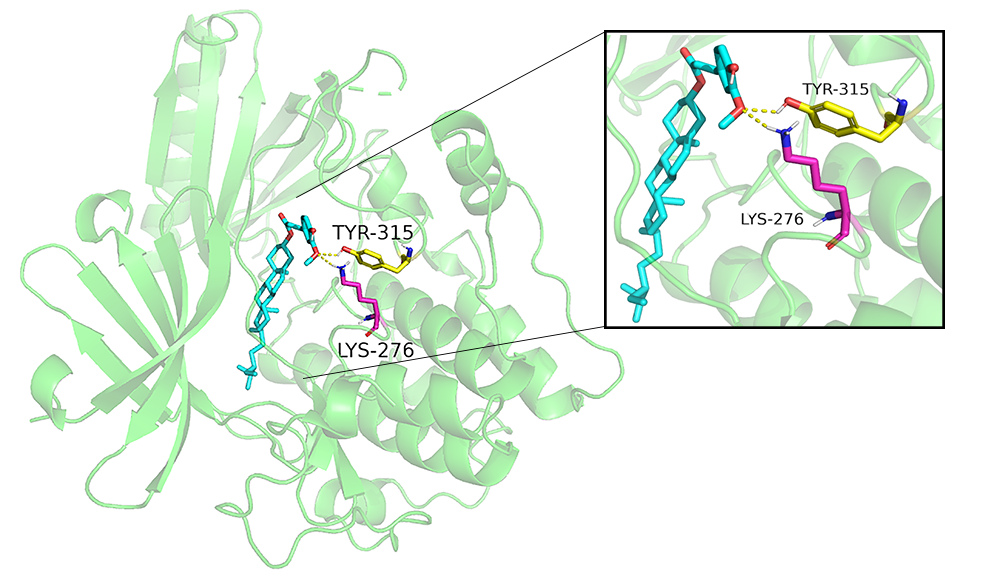

Supplement: Supplementary file 1 [file DataSheet1.ZIP › Figure 5/figure 5(A4).tif]

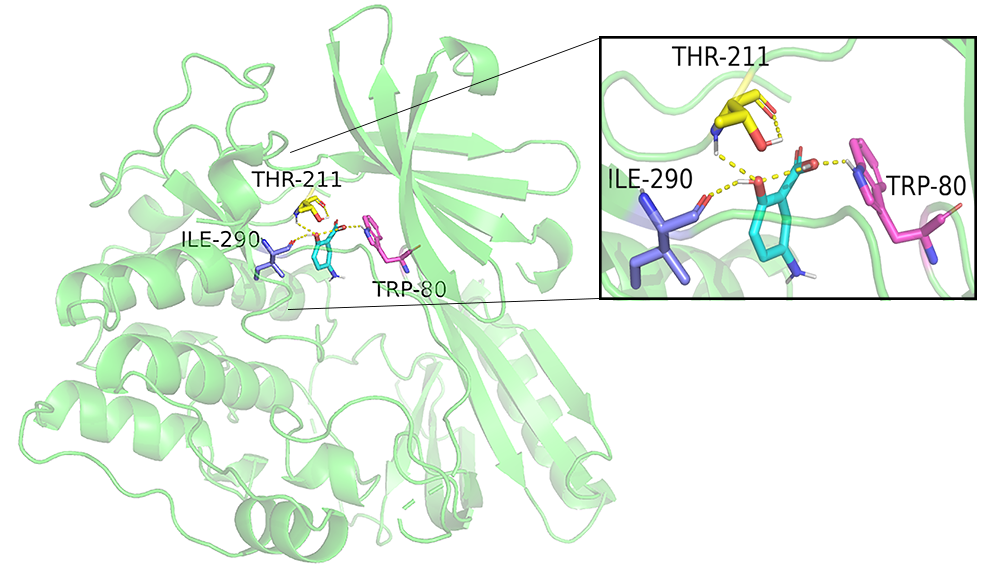

Supplement: Supplementary file 1 [file DataSheet1.ZIP › Figure 5/figure 5(A5).tif]

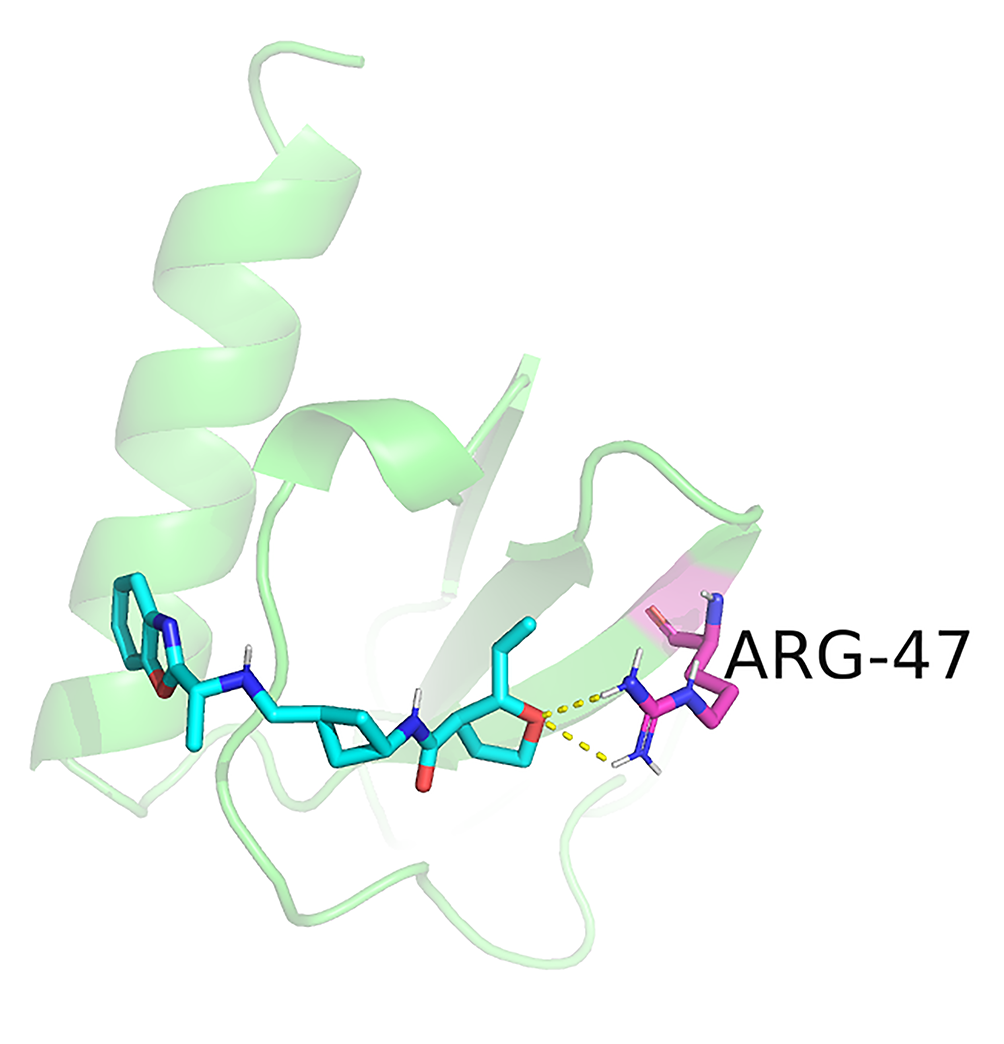

Supplement: Supplementary file 1 [file DataSheet1.ZIP › Figure 5/figure 5(B1).png]

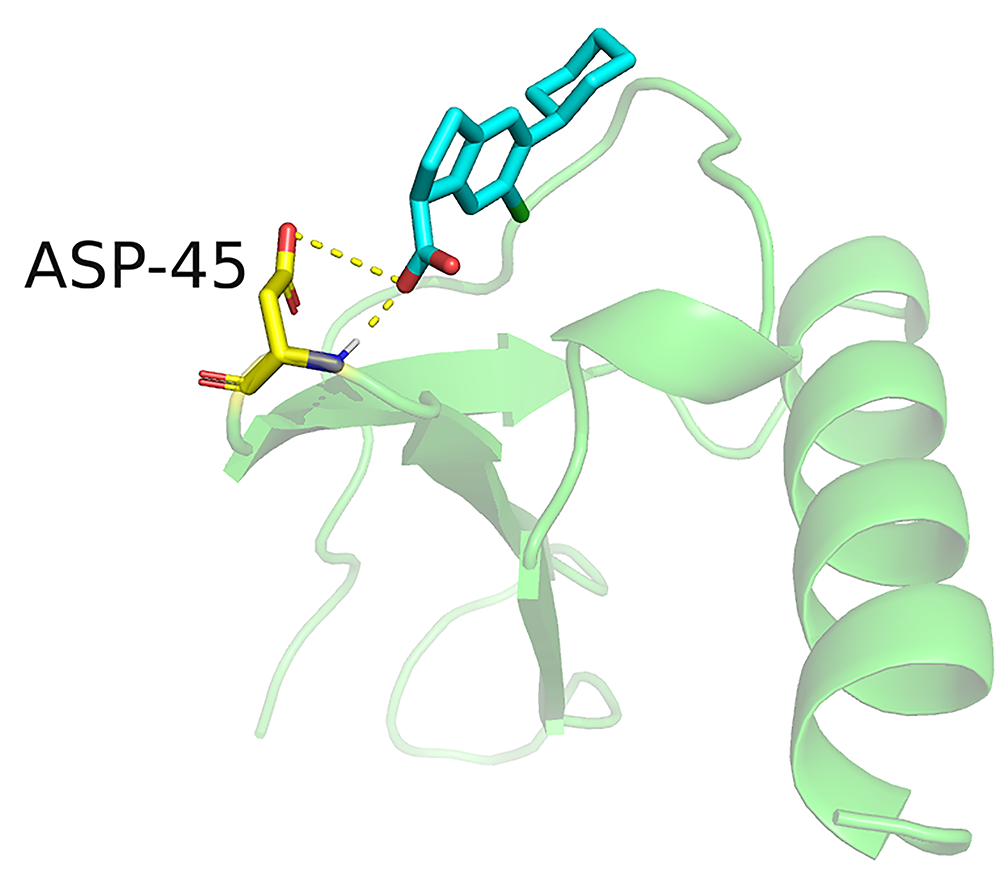

Supplement: Supplementary file 1 [file DataSheet1.ZIP › Figure 5/figure 5(B2).png]

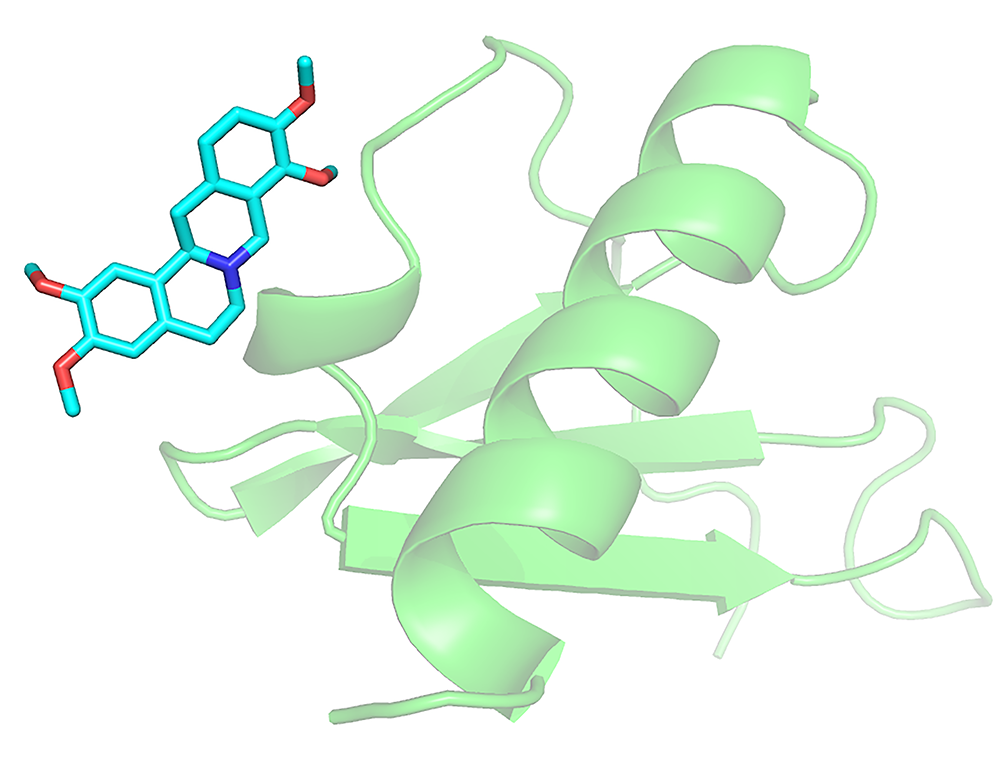

Supplement: Supplementary file 1 [file DataSheet1.ZIP › Figure 5/figure 5(B3).png]

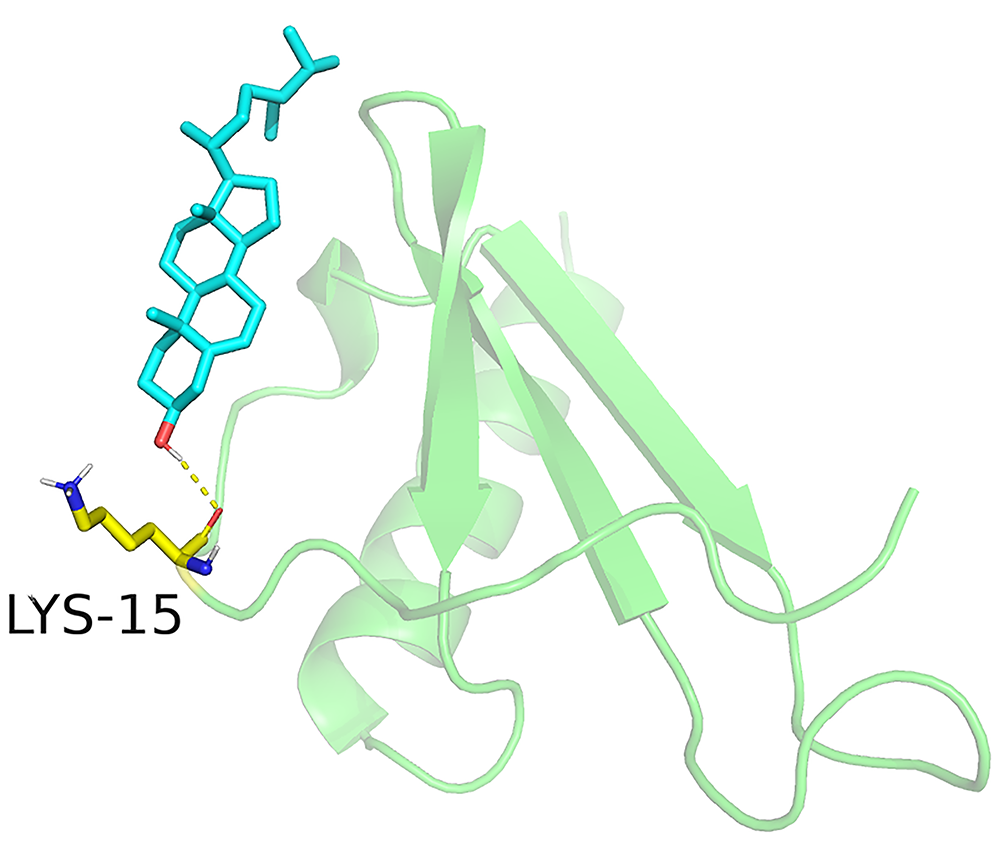

Supplement: Supplementary file 1 [file DataSheet1.ZIP › Figure 5/figure 5(B4).png]

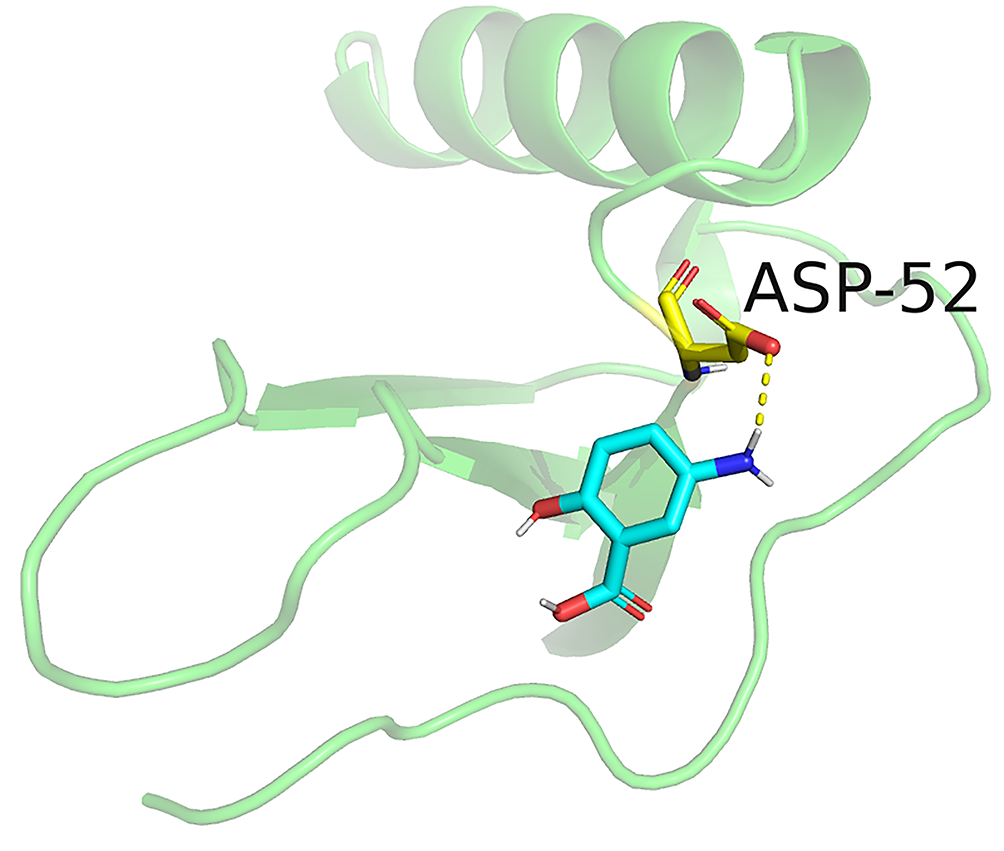

Supplement: Supplementary file 1 [file DataSheet1.ZIP › Figure 5/figure 5(B5).png]

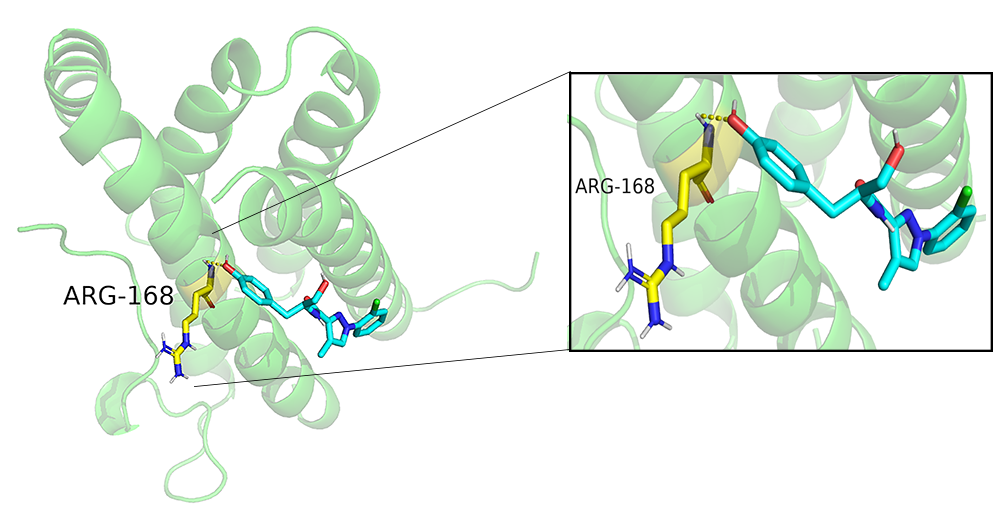

Supplement: Supplementary file 1 [file DataSheet1.ZIP › Figure 5/figure 5(C1).tif]

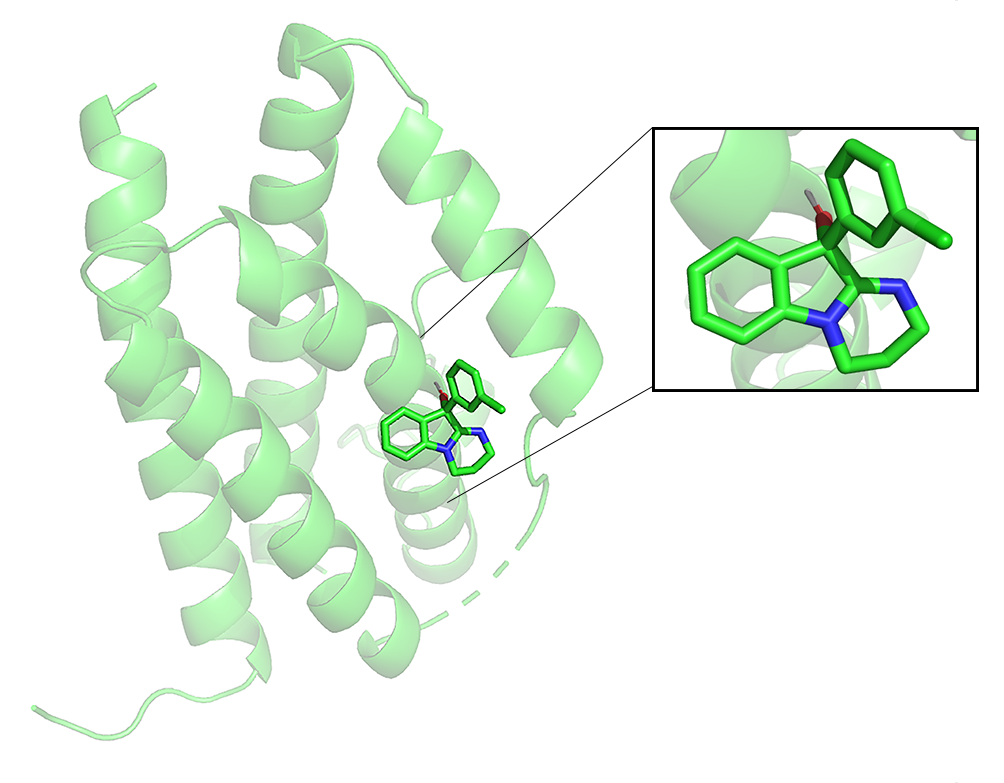

Supplement: Supplementary file 1 [file DataSheet1.ZIP › Figure 5/figure 5(C2).tif]

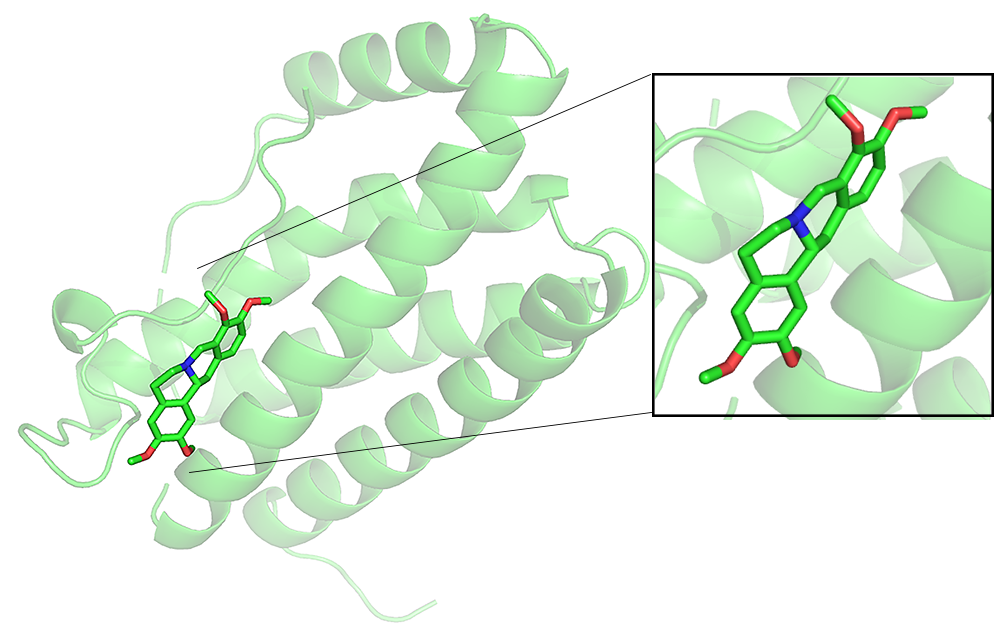

Supplement: Supplementary file 1 [file DataSheet1.ZIP › Figure 5/figure 5(C3).tif]

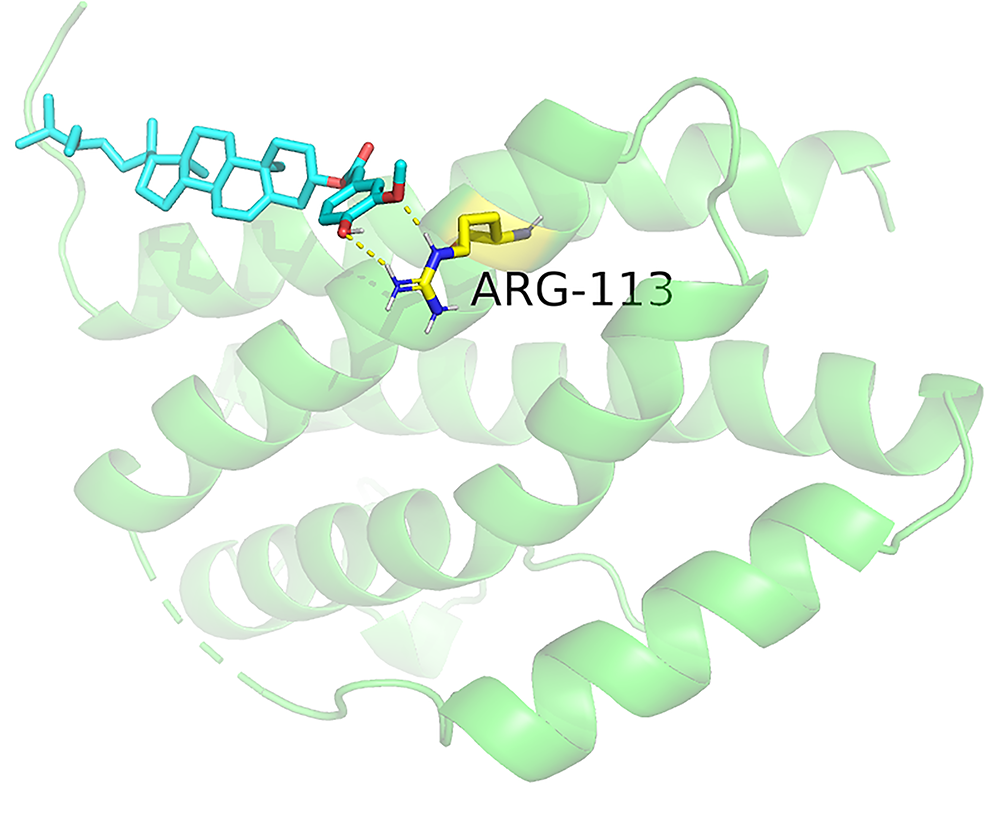

Supplement: Supplementary file 1 [file DataSheet1.ZIP › Figure 5/figure 5(C4).tif]

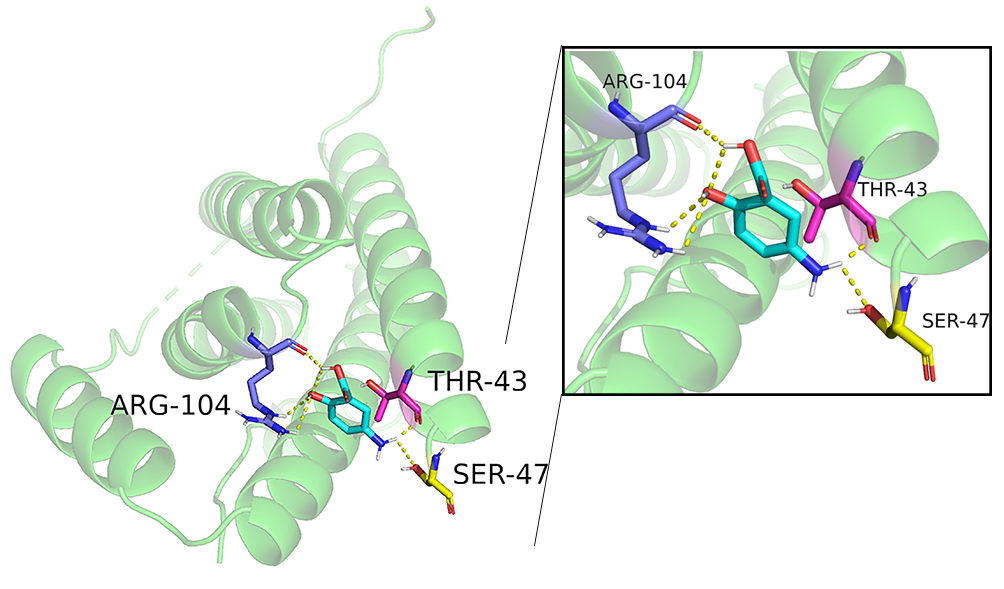

Supplement: Supplementary file 1 [file DataSheet1.ZIP › Figure 5/figure 5(C5).tif]

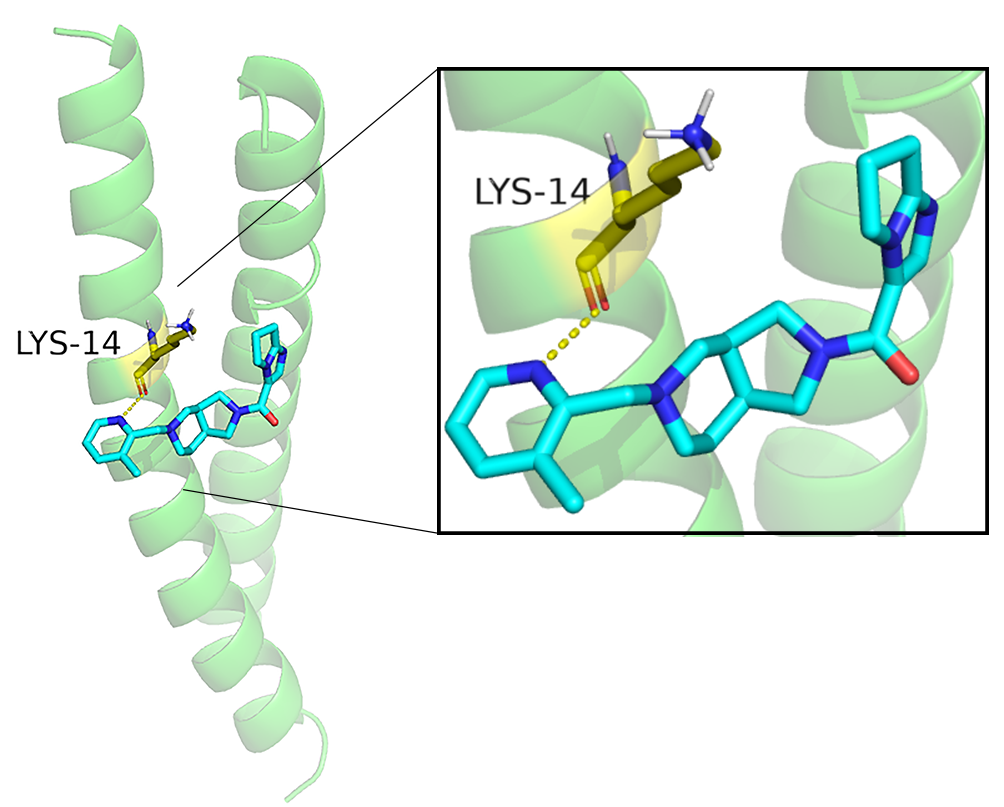

Supplement: Supplementary file 1 [file DataSheet1.ZIP › Figure 5/figure 5(D1).tif]

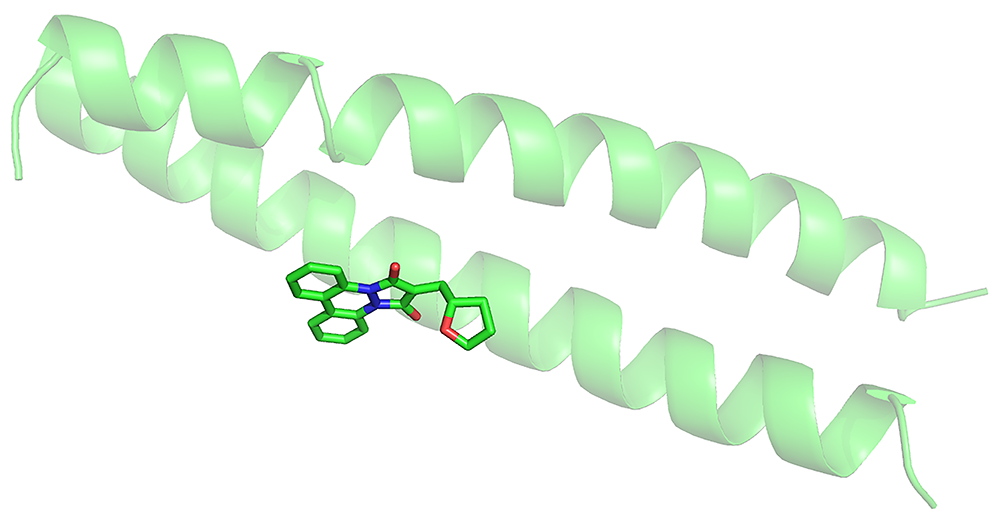

Supplement: Supplementary file 1 [file DataSheet1.ZIP › Figure 5/figure 5(D2).tif]

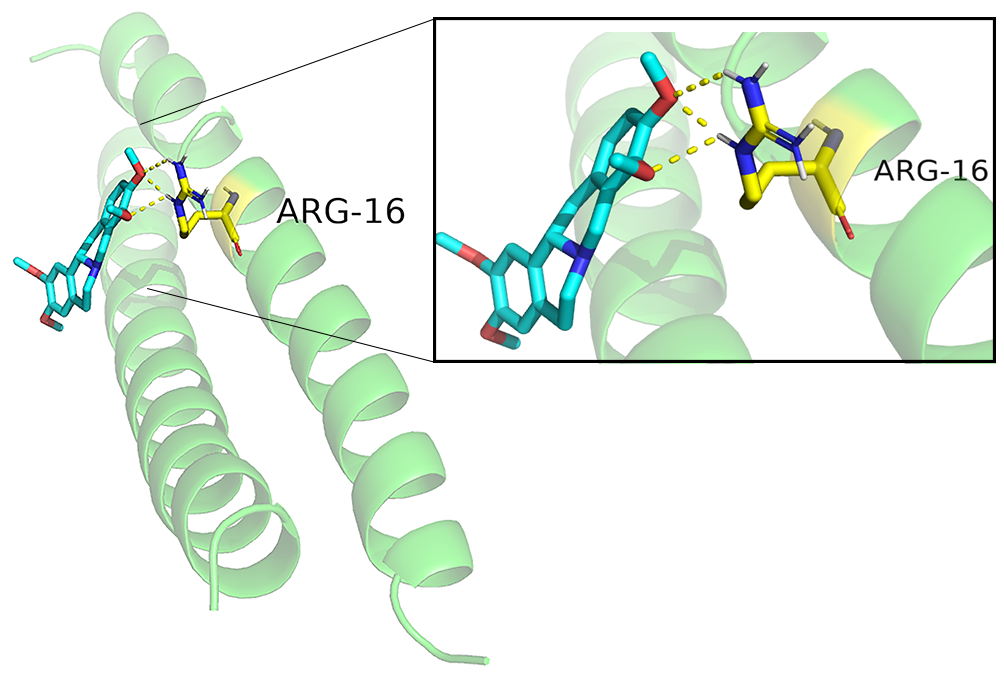

Supplement: Supplementary file 1 [file DataSheet1.ZIP › Figure 5/figure 5(D3).tif]

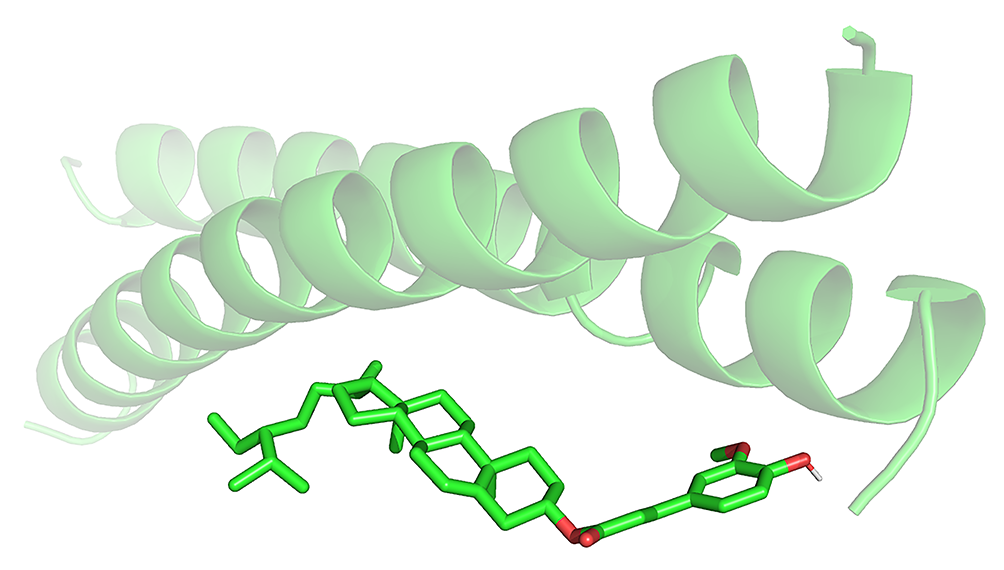

Supplement: Supplementary file 1 [file DataSheet1.ZIP › Figure 5/figure 5(D4).tif]

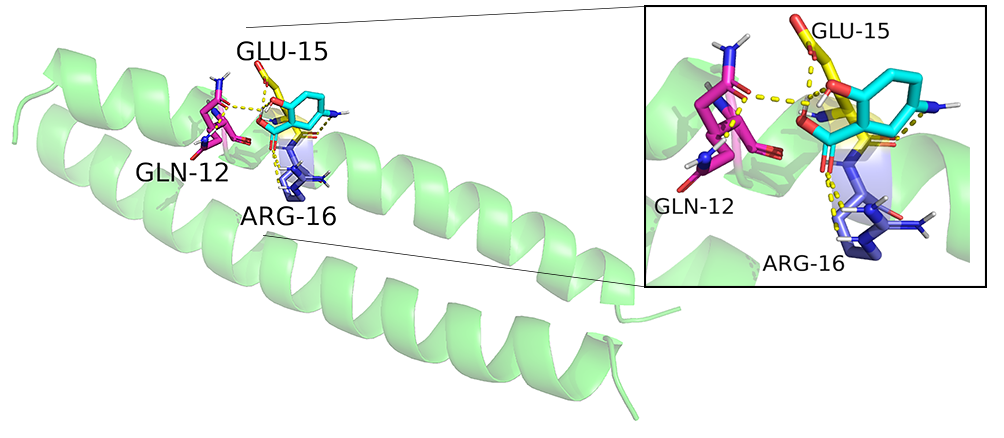

Supplement: Supplementary file 1 [file DataSheet1.ZIP › Figure 5/figure 5(D5).tif]

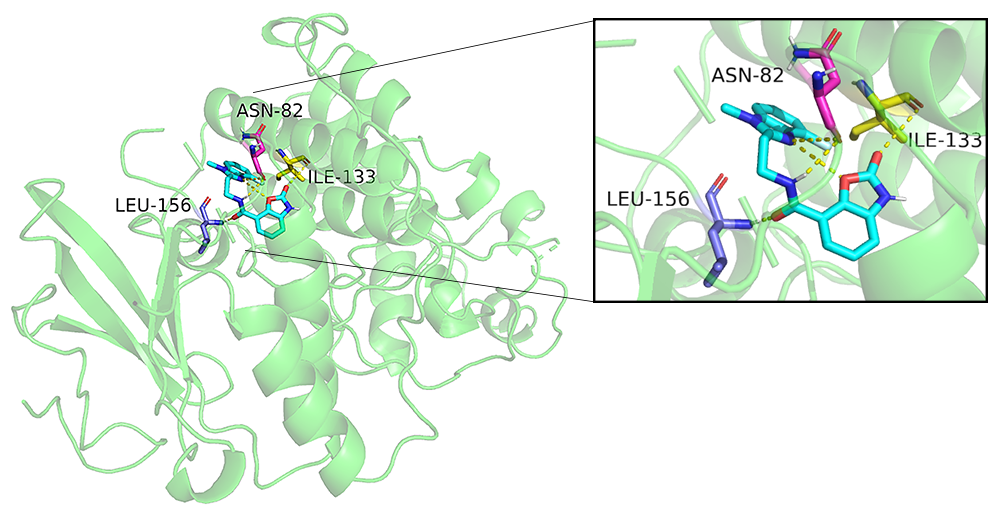

Supplement: Supplementary file 1 [file DataSheet1.ZIP › Figure 5/figure 5(E1).tif]

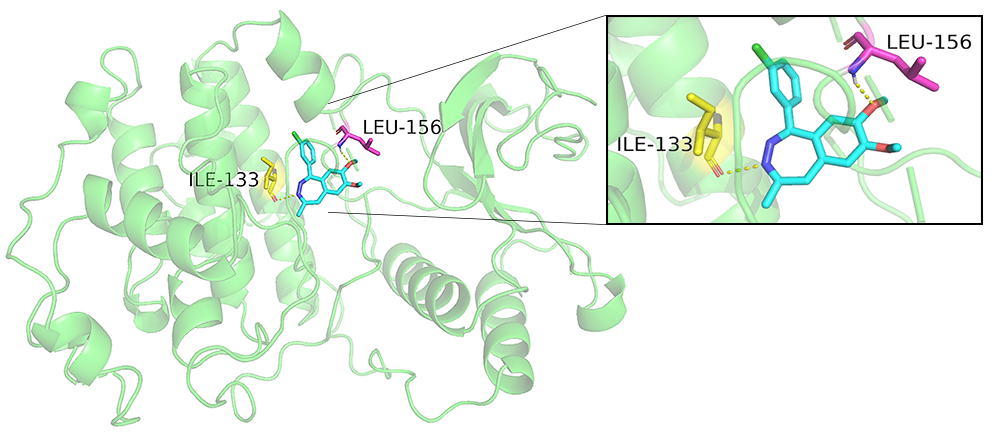

Supplement: Supplementary file 1 [file DataSheet1.ZIP › Figure 5/figure 5(E2).tif]

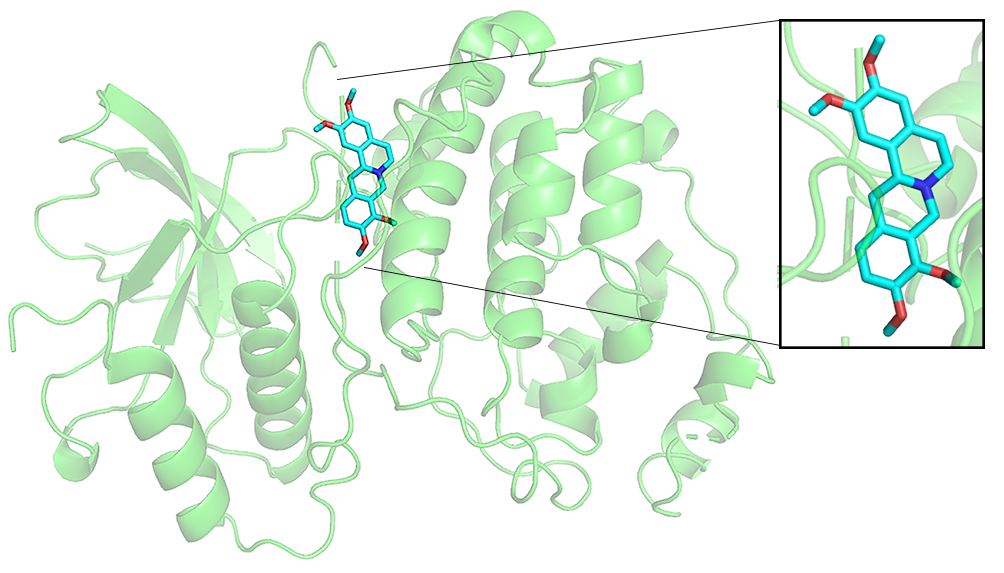

Supplement: Supplementary file 1 [file DataSheet1.ZIP › Figure 5/figure 5(E3).tif]

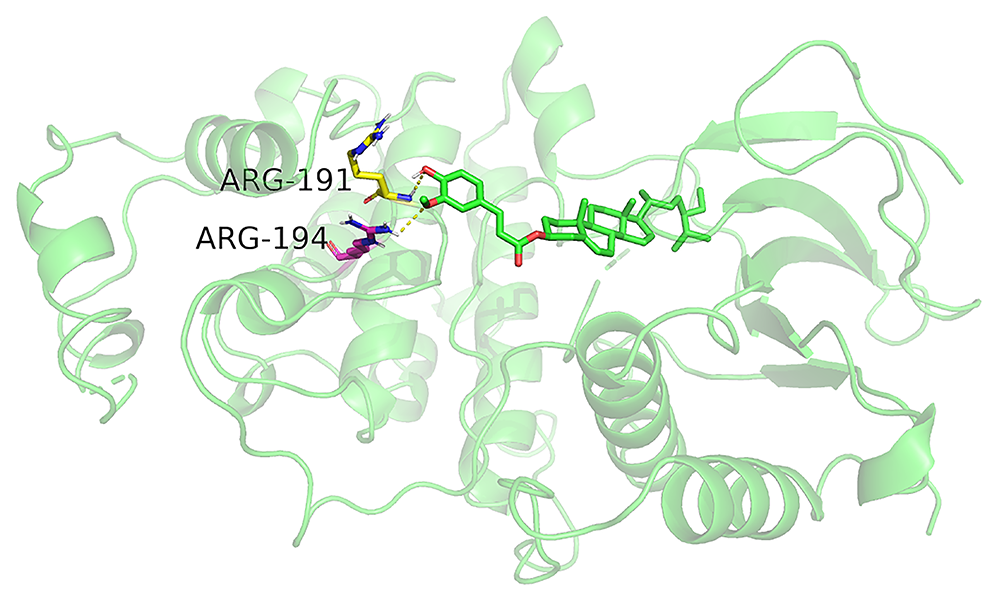

Supplement: Supplementary file 1 [file DataSheet1.ZIP › Figure 5/figure 5(E4).tif]

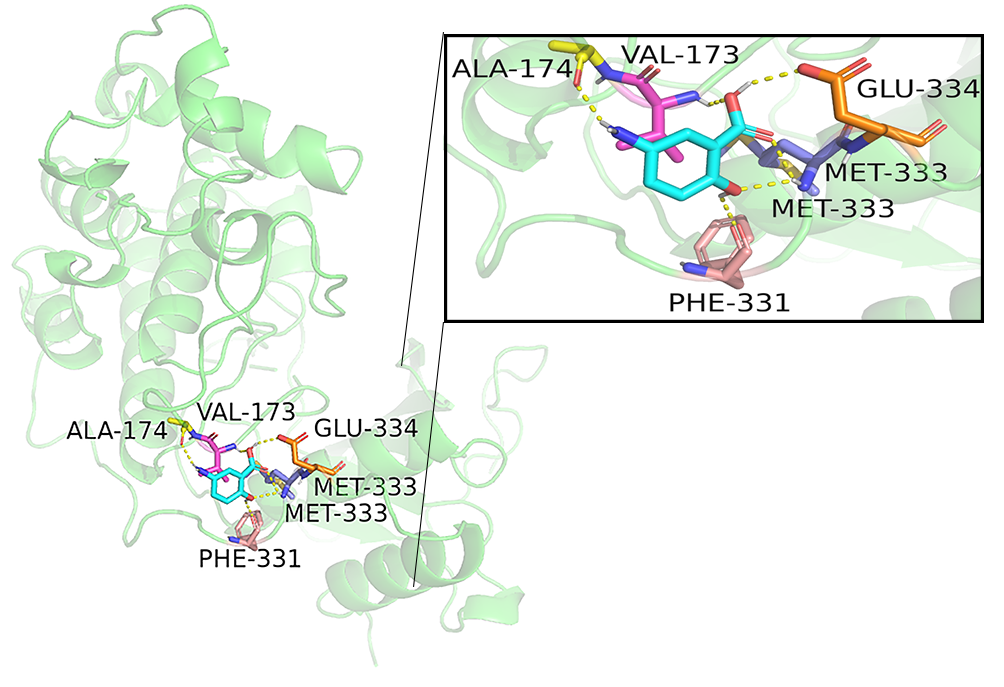

Supplement: Supplementary file 1 [file DataSheet1.ZIP › Figure 5/figure 5(E5).tif]

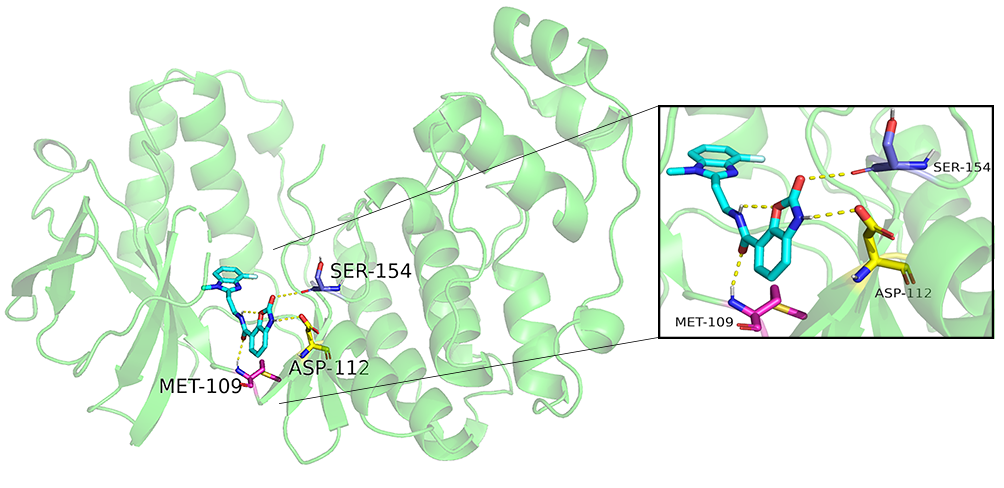

Supplement: Supplementary file 1 [file DataSheet1.ZIP › Figure 5/figure 5(F1).tif]

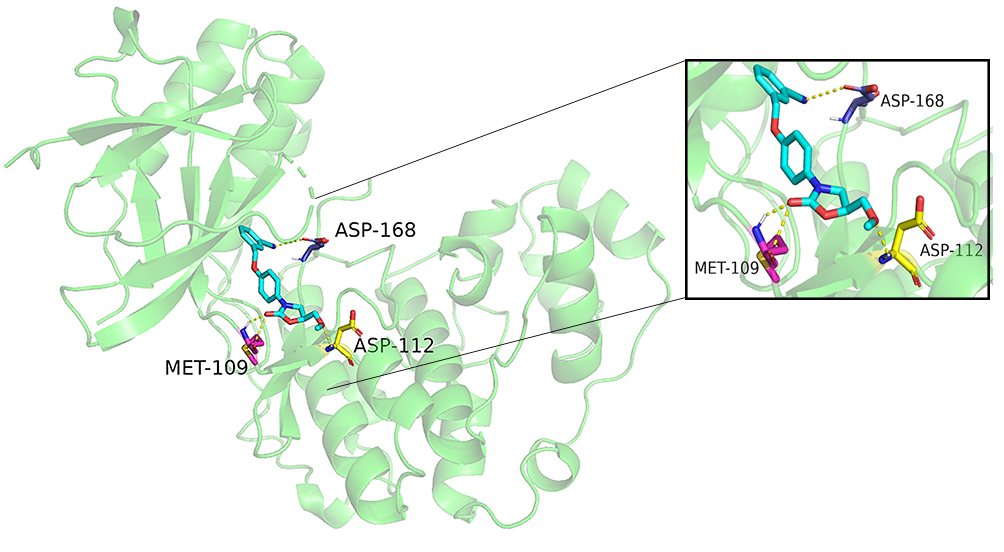

Supplement: Supplementary file 1 [file DataSheet1.ZIP › Figure 5/figure 5(F2).tif]

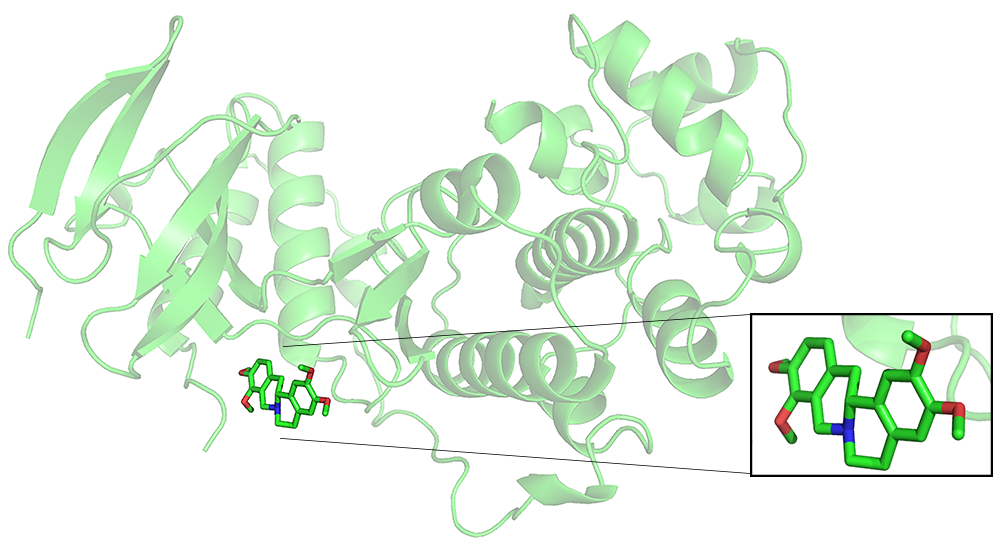

Supplement: Supplementary file 1 [file DataSheet1.ZIP › Figure 5/figure 5(F3).tif]

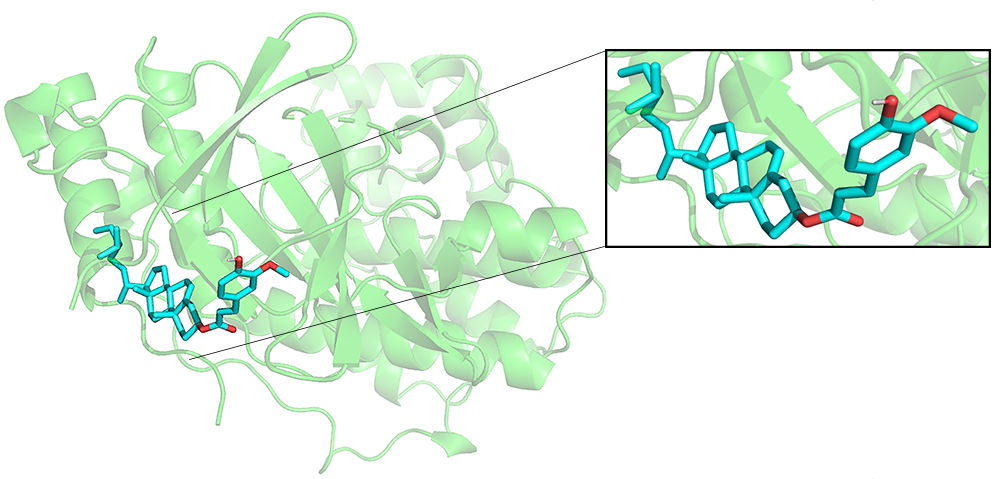

Supplement: Supplementary file 1 [file DataSheet1.ZIP › Figure 5/figure 5(F4).tif]

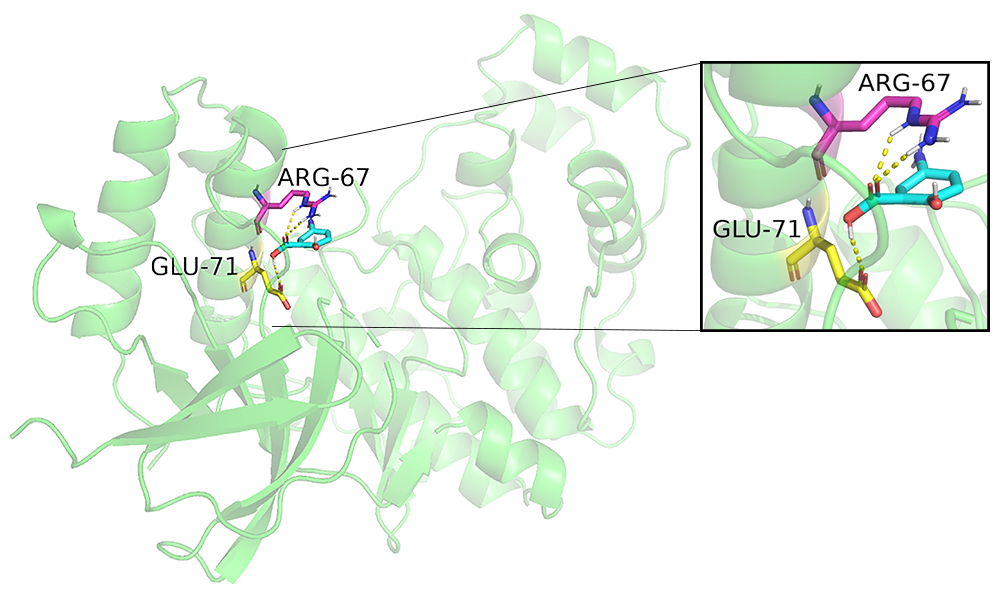

Supplement: Supplementary file 1 [file DataSheet1.ZIP › Figure 5/figure 5(F5).tif]

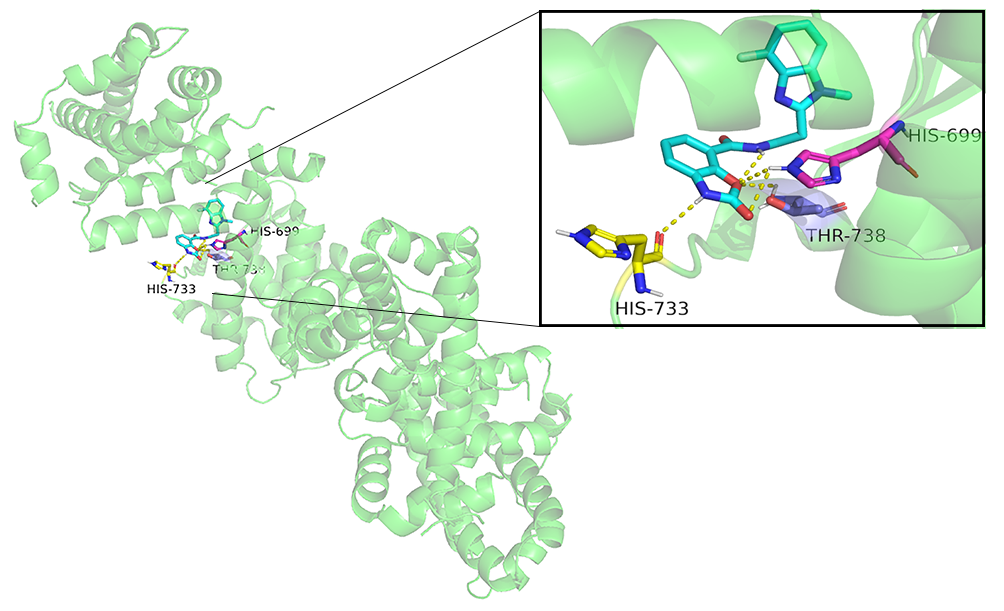

Supplement: Supplementary file 1 [file DataSheet1.ZIP › Figure 5/figure 5(G1).tif]

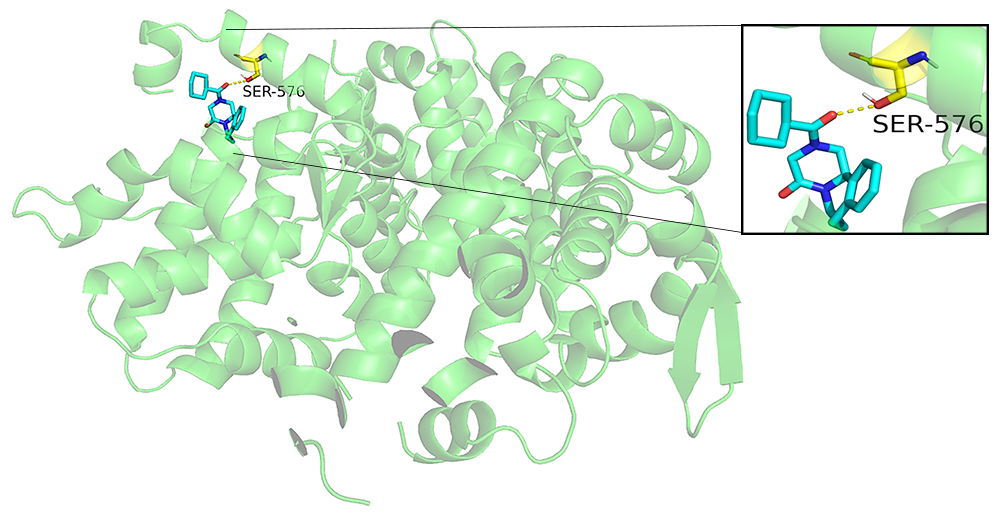

Supplement: Supplementary file 1 [file DataSheet1.ZIP › Figure 5/figure 5(G2).tif]

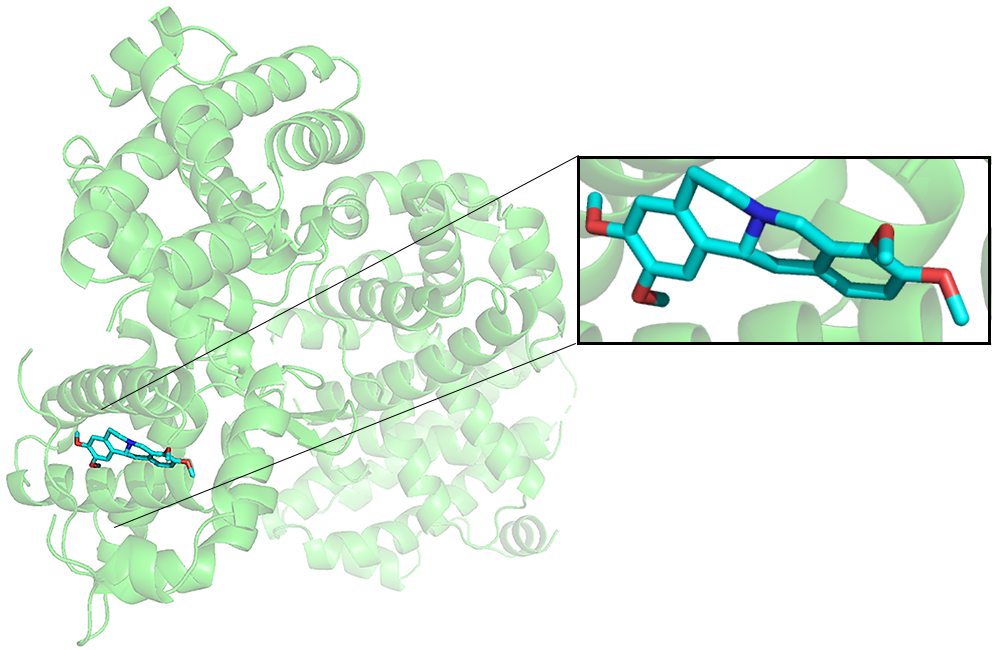

Supplement: Supplementary file 1 [file DataSheet1.ZIP › Figure 5/figure 5(G3).tif]

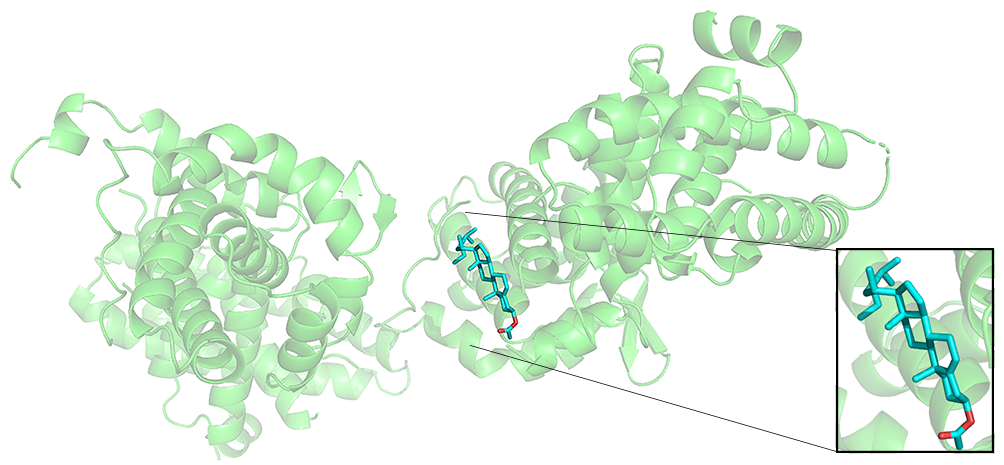

Supplement: Supplementary file 1 [file DataSheet1.ZIP › Figure 5/figure 5(G4).tif]

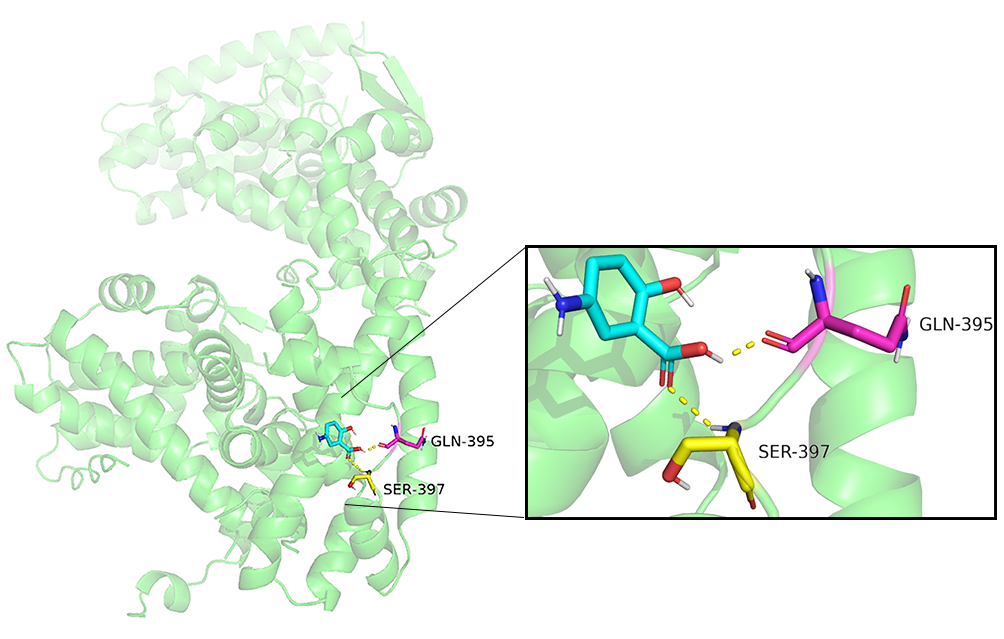

Supplement: Supplementary file 1 [file DataSheet1.ZIP › Figure 5/figure 5(G5).tif]

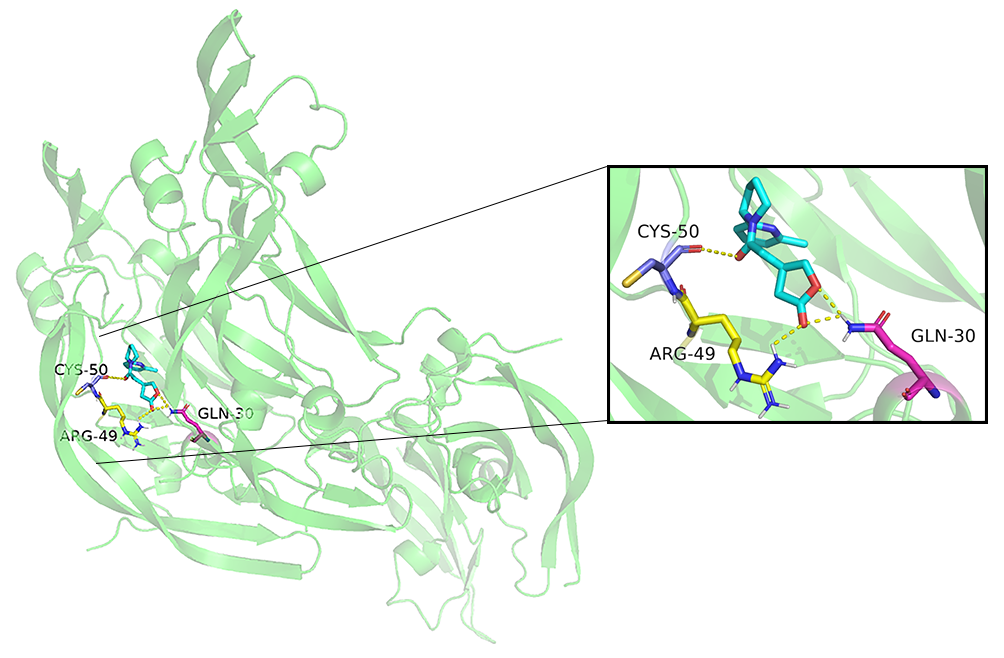

Supplement: Supplementary file 1 [file DataSheet1.ZIP › Figure 5/figure 5(H1).tif]

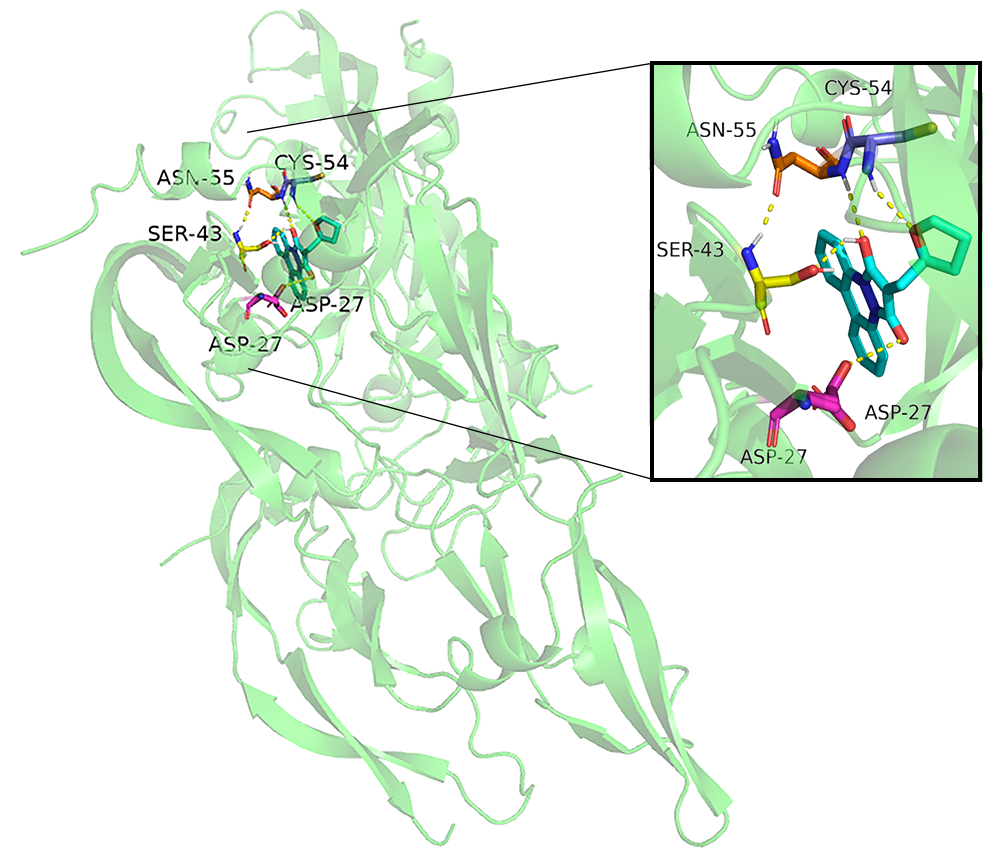

Supplement: Supplementary file 1 [file DataSheet1.ZIP › Figure 5/figure 5(H2).tif]

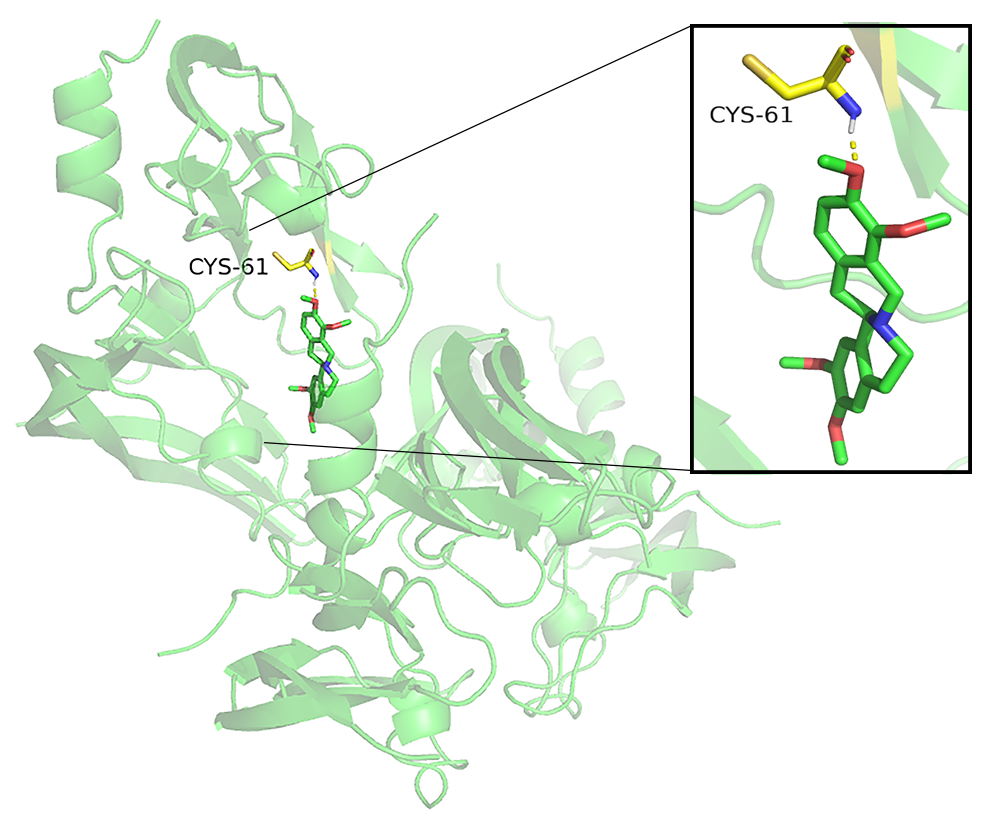

Supplement: Supplementary file 1 [file DataSheet1.ZIP › Figure 5/figure 5(H3).tif]

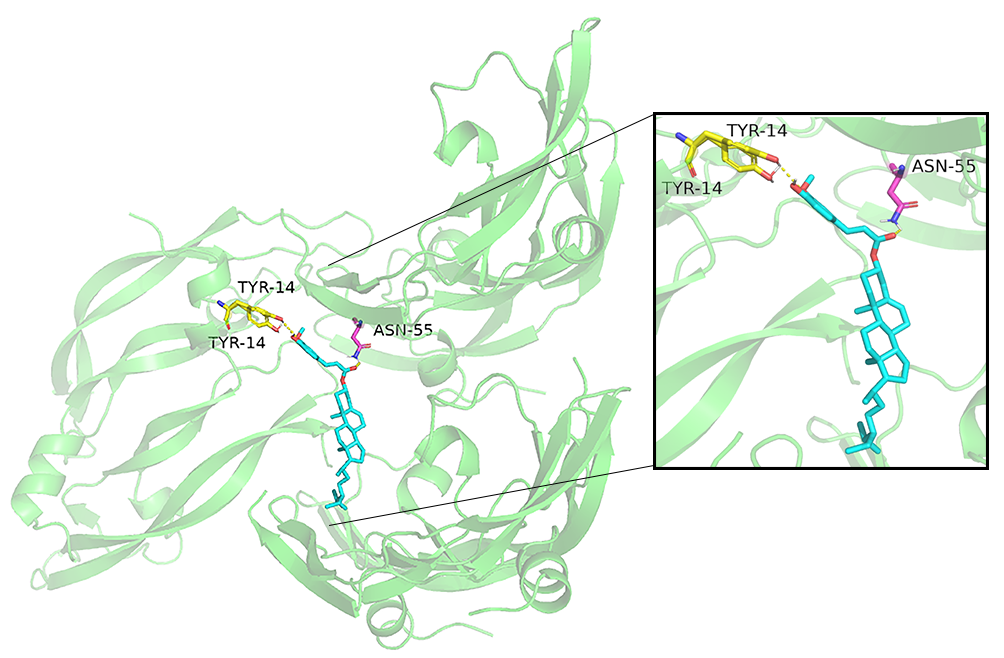

Supplement: Supplementary file 1 [file DataSheet1.ZIP › Figure 5/figure 5(H4).tif]

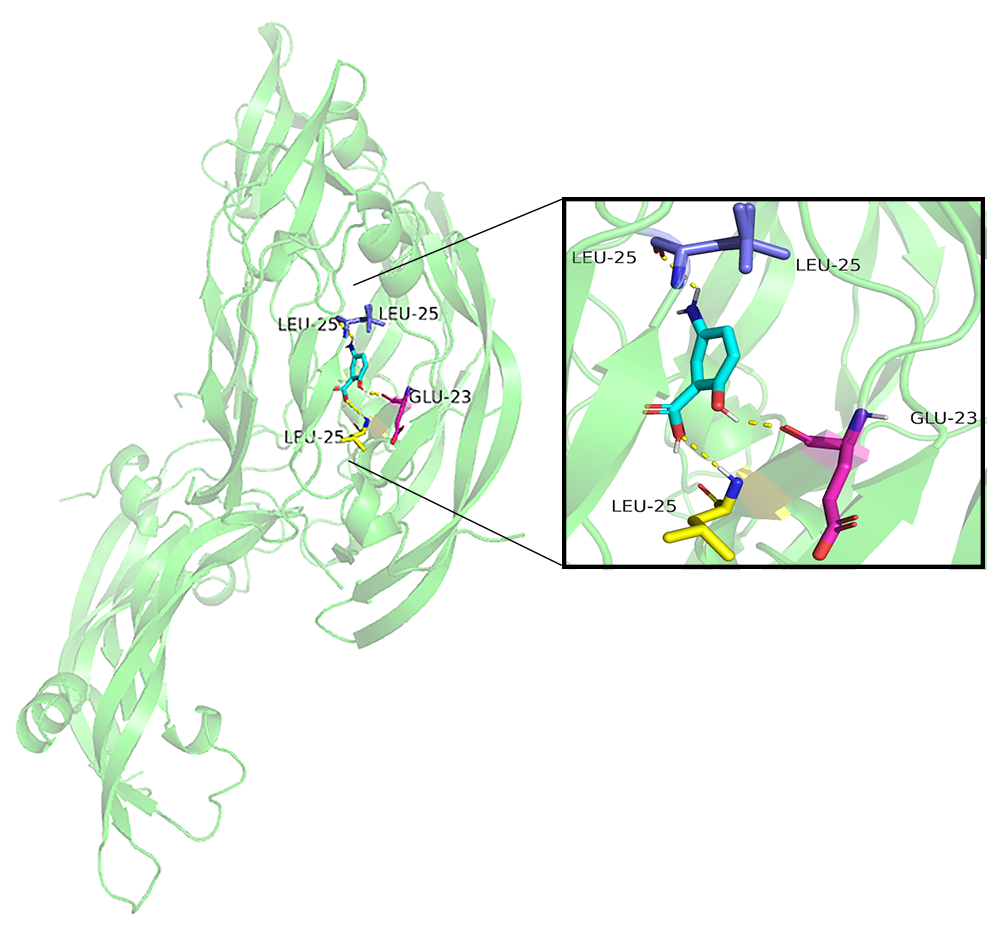

Supplement: Supplementary file 1 [file DataSheet1.ZIP › Figure 5/figure 5(H5).tif]
