# Supplementary material for: Network Pharmacology and Molecular Docking Analysis on Molecular Mechanism of Qingzi Zhitong Decoction in the Treatment of Ulcerative Colitis
Source: Front Pharmacol. 2022 Feb 8;13:727608. doi: 10.3389/fphar.2022.727608 (PMC8883437; doi:10.3389/fphar.2022.727608)
Supplement: Supplementary file 2 [file DataSheet2.docx]

The Affinity of ligands from quercetin mol2 file docking with Akt1

| name | Affinity (kcal/mol) | name | Affinity (kcal/mol) | name | Affinity (kcal/mol) |
| --- | --- | --- | --- | --- | --- |
| ZINC000443798537 | -10.3 | ZINC000651129595 | -9 | ZINC000885990822 | -8.2 |
| ZINC001087997780 | -10 | ZINC000984826022 | -9 | ZINC000984826049 | -8.2 |
| ZINC000191968750 | -9.6 | ZINC000443798551 | -8.9 | ZINC001345354685 | -8.2 |
| ZINC000522492590 | -9.6 | ZINC000443798552 | -8.9 | ZINC000166938211 | -8 |
| ZINC001087997783 | -9.6 | ZINC000443798553 | -8.9 | ZINC000885990838 | -8 |
| ZINC000443798558 | -9.5 | ZINC000984826020 | -8.9 | ZINC000885990839 | -8 |
| ZINC000443798559 | -9.5 | ZINC000191968713 | -8.8 | ZINC000885990840 | -8 |
| ZINC000443798526 | -9.3 | ZINC000984826047 | -8.8 | ZINC000984826035 | -7.9 |
| ZINC000984826057 | -9.3 | ZINC000443798538 | -8.7 | ZINC000984826064 | -7.9 |
| ZINC001087997779 | -9.3 | ZINC001345354687 | -8.7 | ZINC000191968726 | -7.4 |
| ZINC000443798550 | -9.2 | ZINC001345354686 | -8.6 | ZINC000631315932 | -7.4 |
| ZINC000984826053 | -9.2 | ZINC000191968731 | -8.5 | ZINC000885990819 | -6.2 |
| ZINC001087997781 | -9.2 | ZINC000651129593 | -8.4 | ZINC000885990820 | -6 |
| ZINC001087997782 | -9.2 | ZINC000651129594 | -8.3 | ZINC000696770855 | -5.4 |
| ZINC000191968718 | -9 | ZINC000885990821 | -8.3 |  |  |
| ZINC000443798556 | -9 | ZINC000885990823 | -8.3 |  |  |

The Affinity of ligands from quercetin mol2 file docking with CXCL8

| name | Affinity (kcal/mol) | name | Affinity (kcal/mol) | name | Affinity (kcal/mol) |
| --- | --- | --- | --- | --- | --- |
| ZINC000984826057 | -7.1 | ZINC000191968713 | -6.2 | ZINC000885990823 | -5.7 |
| ZINC000191968718 | -7 | ZINC000984826020 | -6.2 | ZINC000885990838 | -5.7 |
| ZINC000443798559 | -7 | ZINC000443798538 | -6.1 | ZINC000984826035 | -5.6 |
| ZINC000443798526 | -6.9 | ZINC000443798550 | -6 | ZINC000984826064 | -5.6 |
| ZINC000443798537 | -6.8 | ZINC000443798552 | -6 | ZINC001087997779 | -5.6 |
| ZINC000984826053 | -6.8 | ZINC000651129594 | -6 | ZINC001345354687 | -5.6 |
| ZINC000443798558 | -6.7 | ZINC000984826047 | -6 | ZINC000443798553 | -5.5 |
| ZINC000522492590 | -6.7 | ZINC000984826049 | -6 | ZINC000651129593 | -5.5 |
| ZINC000984826022 | -6.7 | ZINC001087997782 | -6 | ZINC000885990821 | -5.4 |
| ZINC001087997780 | -6.7 | ZINC001087997783 | -6 | ZINC001345354686 | -5.4 |
| ZINC000166938211 | -6.4 | ZINC000885990839 | -5.9 | ZINC000631315932 | -5 |
| ZINC000191968731 | -6.3 | ZINC000191968726 | -5.8 | ZINC000885990820 | -4.8 |
| ZINC000191968750 | -6.3 | ZINC000443798556 | -5.8 | ZINC000885990819 | -4.6 |
| ZINC000443798551 | -6.3 | ZINC000885990840 | -5.8 | ZINC000696770855 | -4.5 |
| ZINC000651129595 | -6.3 | ZINC001345354685 | -5.8 |  |  |
| ZINC001087997781 | -6.3 | ZINC000885990822 | -5.7 |  |  |

The Affinity of ligands from quercetin mol2 file docking with IL-6

| name | Affinity (kcal/mol) | name | Affinity (kcal/mol) | name | Affinity (kcal/mol) |
| --- | --- | --- | --- | --- | --- |
| ZINC000191968750 | -7.3 | ZINC000651129593 | -6.4 | ZINC000885990822 | -5.8 |
| ZINC000522492590 | -7.2 | ZINC000984826020 | -6.4 | ZINC001345354685 | -5.8 |
| ZINC000443798537 | -7.1 | ZINC000984826022 | -6.4 | ZINC000984826049 | -5.7 |
| ZINC001087997783 | -7 | ZINC000984826047 | -6.4 | ZINC000885990838 | -5.6 |
| ZINC000166938211 | -6.9 | ZINC001087997782 | -6.4 | ZINC000885990839 | -5.6 |
| ZINC000191968713 | -6.9 | ZINC001087997781 | -6.3 | ZINC000885990840 | -5.5 |
| ZINC000443798559 | -6.9 | ZINC000191968731 | -6.2 | ZINC000984826035 | -5.5 |
| ZINC000443798558 | -6.7 | ZINC000443798550 | -6.2 | ZINC000885990821 | -5.4 |
| ZINC000984826053 | -6.7 | ZINC000443798552 | -6.2 | ZINC000984826064 | -5.4 |
| ZINC001087997780 | -6.7 | ZINC001087997779 | -6.2 | ZINC001345354687 | -5.3 |
| ZINC000443798526 | -6.5 | ZINC001345354686 | -6.2 | ZINC000631315932 | -5 |
| ZINC000443798538 | -6.5 | ZINC000191968718 | -6 | ZINC000885990820 | -4.8 |
| ZINC000443798551 | -6.5 | ZINC000191968726 | -6 | ZINC000885990819 | -4.4 |
| ZINC000443798553 | -6.5 | ZINC000651129595 | -6 | ZINC000696770855 | -4.3 |
| ZINC000443798556 | -6.5 | ZINC000885990823 | -6 |  |  |
| ZINC000984826057 | -6.5 | ZINC000651129594 | -5.8 |  |  |

The Affinity of ligands from quercetin mol2 file docking with JUN

| name | Affinity (kcal/mol) | name | Affinity (kcal/mol) | name | Affinity (kcal/mol) |
| --- | --- | --- | --- | --- | --- |
| ZINC001087997780 | -7.1 | ZINC001087997782 | -6.1 | ZINC000885990822 | -5.5 |
| ZINC000191968750 | -6.6 | ZINC000191968713 | -6 | ZINC000984826064 | -5.5 |
| ZINC000522492590 | -6.4 | ZINC000166938211 | -5.9 | ZINC000885990821 | -5.4 |
| ZINC001087997783 | -6.4 | ZINC000443798551 | -5.9 | ZINC000885990839 | -5.4 |
| ZINC000191968718 | -6.3 | ZINC000984826057 | -5.9 | ZINC000885990840 | -5.4 |
| ZINC000443798537 | -6.3 | ZINC000443798552 | -5.8 | ZINC000984826035 | -5.4 |
| ZINC000443798553 | -6.3 | ZINC000443798556 | -5.8 | ZINC001345354686 | -5.4 |
| ZINC000443798559 | -6.3 | ZINC000984826020 | -5.8 | ZINC001345354687 | -5.4 |
| ZINC000984826053 | -6.3 | ZINC001345354685 | -5.8 | ZINC000191968726 | -5.2 |
| ZINC000443798550 | -6.2 | ZINC000443798538 | -5.7 | ZINC000984826047 | -5.2 |
| ZINC001087997779 | -6.2 | ZINC000443798558 | -5.7 | ZINC000631315932 | -4.9 |
| ZINC000191968731 | -6.1 | ZINC000651129594 | -5.7 | ZINC000885990819 | -4.4 |
| ZINC000443798526 | -6.1 | ZINC000885990823 | -5.7 | ZINC000885990820 | -4.4 |
| ZINC000651129593 | -6.1 | ZINC000885990838 | -5.6 | ZINC000696770855 | -4.2 |
| ZINC000651129595 | -6.1 | ZINC000984826022 | -5.6 |  |  |
| ZINC001087997781 | -6.1 | ZINC000984826049 | -5.6 |  |  |

The Affinity of ligands from quercetin mol2 file docking with MAPK1

| name | Affinity (kcal/mol) | name | Affinity (kcal/mol) | name | Affinity (kcal/mol) |
| --- | --- | --- | --- | --- | --- |
| ZINC000443798537 | -9.8 | ZINC001087997781 | -7.9 | ZINC000443798538 | -7.1 |
| ZINC000651129595 | -9.6 | ZINC000443798551 | -7.8 | ZINC001345354685 | -7.1 |
| ZINC000443798550 | -8.6 | ZINC000443798552 | -7.8 | ZINC000885990840 | -7 |
| ZINC000984826057 | -8.6 | ZINC000651129593 | -7.8 | ZINC001345354686 | -6.9 |
| ZINC001087997780 | -8.4 | ZINC000885990823 | -7.8 | ZINC000984826049 | -6.8 |
| ZINC000191968718 | -8.3 | ZINC000984826020 | -7.8 | ZINC000885990838 | -6.7 |
| ZINC000443798558 | -8.2 | ZINC000984826022 | -7.8 | ZINC000984826035 | -6.7 |
| ZINC000443798559 | -8.2 | ZINC000522492590 | -7.7 | ZINC000651129594 | -6.6 |
| ZINC000191968731 | -8.1 | ZINC000984826053 | -7.7 | ZINC001345354687 | -6.6 |
| ZINC001087997779 | -8.1 | ZINC001087997782 | -7.7 | ZINC000885990839 | -6.3 |
| ZINC001087997783 | -8.1 | ZINC000443798526 | -7.6 | ZINC000984826064 | -6.3 |
| ZINC000191968713 | -8 | ZINC000443798556 | -7.6 | ZINC000885990819 | -6.2 |
| ZINC000885990821 | -8 | ZINC000443798553 | -7.5 | ZINC000885990820 | -6 |
| ZINC000166938211 | -7.9 | ZINC000984826047 | -7.4 | ZINC000696770855 | -5.8 |
| ZINC000191968750 | -7.9 | ZINC000191968726 | -7.2 |  |  |
| ZINC000885990822 | -7.9 | ZINC000631315932 | -7.2 |  |  |

The Affinity of ligands from quercetin mol2 file docking with MAPK14

| name | Affinity (kcal/mol) | name | Affinity (kcal/mol) | name | Affinity (kcal/mol) |
| --- | --- | --- | --- | --- | --- |
| ZINC000443798537 | -8.8 | ZINC000984826057 | -7.5 | ZINC000885990822 | -6.9 |
| ZINC001087997780 | -8.8 | ZINC000651129593 | -7.4 | ZINC000984826047 | -6.9 |
| ZINC000522492590 | -8.2 | ZINC000651129594 | -7.4 | ZINC000885990840 | -6.8 |
| ZINC001087997782 | -8.2 | ZINC000885990821 | -7.4 | ZINC001345354686 | -6.7 |
| ZINC000443798551 | -8.1 | ZINC000885990823 | -7.4 | ZINC000191968726 | -6.6 |
| ZINC001087997781 | -8 | ZINC000191968731 | -7.3 | ZINC000984826049 | -6.6 |
| ZINC001087997783 | -7.9 | ZINC000443798526 | -7.3 | ZINC000984826064 | -6.6 |
| ZINC000166938211 | -7.8 | ZINC000443798553 | -7.3 | ZINC000631315932 | -6.4 |
| ZINC000443798558 | -7.8 | ZINC000443798559 | -7.3 | ZINC000984826035 | -6.4 |
| ZINC000191968718 | -7.7 | ZINC000984826053 | -7.3 | ZINC000885990838 | -6.3 |
| ZINC000443798552 | -7.7 | ZINC000984826020 | -7.2 | ZINC000885990839 | -6.2 |
| ZINC000191968750 | -7.6 | ZINC000984826022 | -7.2 | ZINC000885990820 | -5.7 |
| ZINC000443798550 | -7.6 | ZINC001345354685 | -7.2 | ZINC000885990819 | -5.2 |
| ZINC000651129595 | -7.6 | ZINC000443798538 | -7.1 | ZINC000696770855 | -5.1 |
| ZINC001087997779 | -7.6 | ZINC000443798556 | -7 |  |  |
| ZINC000191968713 | -7.5 | ZINC001345354687 | -7 |  |  |

The Affinity of ligands from quercetin mol2 file docking with RB1

| name | Affinity (kcal/mol) | name | Affinity (kcal/mol) | name | Affinity (kcal/mol) |
| --- | --- | --- | --- | --- | --- |
| ZINC000443798537 | -9 | ZINC000443798538 | -7.4 | ZINC001345354685 | -6.8 |
| ZINC000191968750 | -8.3 | ZINC000443798550 | -7.4 | ZINC000885990823 | -6.6 |
| ZINC000443798553 | -8 | ZINC000443798551 | -7.4 | ZINC000984826035 | -6.5 |
| ZINC000443798556 | -8 | ZINC000984826022 | -7.4 | ZINC000885990840 | -6.4 |
| ZINC001087997780 | -8 | ZINC001087997783 | -7.4 | ZINC001345354687 | -6.4 |
| ZINC000443798526 | -7.9 | ZINC000191968718 | -7.3 | ZINC000885990839 | -6.3 |
| ZINC000443798558 | -7.8 | ZINC000443798552 | -7.3 | ZINC000984826049 | -6.3 |
| ZINC000191968713 | -7.7 | ZINC000166938211 | -7.2 | ZINC000984826064 | -6.3 |
| ZINC000522492590 | -7.6 | ZINC000651129595 | -7.2 | ZINC001345354686 | -6.3 |
| ZINC000984826020 | -7.6 | ZINC000651129593 | -7.1 | ZINC000631315932 | -6.1 |
| ZINC000984826057 | -7.6 | ZINC000191968731 | -7 | ZINC000885990838 | -6.1 |
| ZINC000984826047 | -7.5 | ZINC000885990821 | -7 | ZINC000885990820 | -5.8 |
| ZINC000984826053 | -7.5 | ZINC000443798559 | -6.9 | ZINC000885990819 | -5.7 |
| ZINC001087997781 | -7.5 | ZINC000651129594 | -6.9 | ZINC000696770855 | -5.3 |
| ZINC001087997782 | -7.5 | ZINC000885990822 | -6.9 |  |  |
| ZINC000191968726 | -7.4 | ZINC001087997779 | -6.9 |  |  |

The Affinity of ligands from quercetin mol2 file docking with RELA

| name | Affinity (kcal/mol) | name | Affinity (kcal/mol) | name | Affinity (kcal/mol) |
| --- | --- | --- | --- | --- | --- |
| ZINC000443798537 | -4.7 | ZINC000443798551 | -3.8 | ZINC000443798556 | -3.4 |
| ZINC000191968718 | -4.6 | ZINC000443798552 | -3.8 | ZINC000651129594 | -3.4 |
| ZINC000443798559 | -4.3 | ZINC000984826020 | -3.8 | ZINC000885990821 | -3.4 |
| ZINC000984826022 | -4.2 | ZINC001345354686 | -3.8 | ZINC000885990838 | -3.4 |
| ZINC000443798558 | -4.1 | ZINC001345354687 | -3.8 | ZINC000885990839 | -3.4 |
| ZINC000651129593 | -4.1 | ZINC000885990823 | -3.7 | ZINC000443798538 | -3.3 |
| ZINC000651129595 | -4.1 | ZINC000984826064 | -3.7 | ZINC000443798553 | -3.3 |
| ZINC001087997782 | -4.1 | ZINC000166938211 | -3.6 | ZINC000885990840 | -3.3 |
| ZINC000191968750 | -4 | ZINC000984826049 | -3.6 | ZINC000984826047 | -3.1 |
| ZINC000522492590 | -4 | ZINC001087997783 | -3.6 | ZINC001345354685 | -3 |
| ZINC000984826053 | -4 | ZINC000191968726 | -3.5 | ZINC000885990819 | -2.9 |
| ZINC000984826057 | -4 | ZINC000631315932 | -3.5 | ZINC000191968713 | -2.8 |
| ZINC001087997780 | -4 | ZINC000885990822 | -3.5 | ZINC000885990820 | -2.5 |
| ZINC001087997781 | -4 | ZINC000984826035 | -3.5 | ZINC000696770855 | -2.4 |
| ZINC000443798550 | -3.9 | ZINC001087997779 | -3.5 |  |  |
| ZINC000443798526 | -3.8 | ZINC000191968731 | -3.4 |  |  |

The Affinity of ligands from quercetin mol2 file docking with VEGFA

| name | Affinity (kcal/mol) | name | Affinity (kcal/mol) | name | Affinity (kcal/mol) |
| --- | --- | --- | --- | --- | --- |
| ZINC000166938211 | -7.5 | ZINC000522492590 | -8.4 | ZINC000984826035 | -6.5 |
| ZINC000191968713 | -7.6 | ZINC000631315932 | -6.5 | ZINC000984826047 | -7.1 |
| ZINC000191968718 | -8.1 | ZINC000651129593 | -7.5 | ZINC000984826049 | -6.9 |
| ZINC000191968726 | -6.6 | ZINC000651129594 | -6.5 | ZINC000984826053 | -7.5 |
| ZINC000191968731 | -7.1 | ZINC000651129595 | -7.7 | ZINC000984826057 | -7.5 |
| ZINC000191968750 | -7.7 | ZINC000696770855 | -5.3 | ZINC000984826064 | -6.5 |
| ZINC000443798526 | -7.7 | ZINC000885990819 | -5.5 | ZINC001087997779 | -7.3 |
| ZINC000443798537 | -8.5 | ZINC000885990820 | -5.5 | ZINC001087997780 | -8.7 |
| ZINC000443798538 | -6.8 | ZINC000885990821 | -6.6 | ZINC001087997781 | -7.6 |
| ZINC000443798550 | -7.4 | ZINC000885990822 | -6.5 | ZINC001087997782 | -7.5 |
| ZINC000443798551 | -7.3 | ZINC000885990823 | -7.5 | ZINC001087997783 | -8.0 |
| ZINC000443798552 | -7.1 | ZINC000885990838 | -6.4 | ZINC001345354685 | -7.0 |
| ZINC000443798553 | -7.7 | ZINC000885990839 | -6.5 | ZINC001345354686 | -6.9 |
| ZINC000443798556 | -7.8 | ZINC000885990840 | -6.8 | ZINC001345354687 | -7.0 |
| ZINC000443798558 | -8.1 | ZINC000984826020 | -7.2 |  |  |
| ZINC000443798559 | -8.0 | ZINC000984826022 | -7.3 |  |  |

The Affinity of ligands from luteolin mol2 file docking with Akt1

| name | Affinity (kcal/mol) | name | Affinity (kcal/mol) | name | Affinity (kcal/mol) | name | Affinity (kcal/mol) | name | Affinity (kcal/mol) | name | Affinity (kcal/mol) |
| --- | --- | --- | --- | --- | --- | --- | --- | --- | --- | --- | --- |
| ZINC000000000152 | -11.3 | ZINC000000000100 | -8.8 | ZINC000000000370 | -8.2 | ZINC000000000122 | -7.6 | ZINC000000000271 | -7 | ZINC000000000491 | -6.3 |
| ZINC000000000504 | -11.1 | ZINC000000000277 | -8.8 | ZINC000000000383 | -8.2 | ZINC000000000123 | -7.6 | ZINC000000000388 | -7 | ZINC000000000559 | -6.3 |
| ZINC000000000554 | -10.6 | ZINC000000000397 | -8.8 | ZINC000000000463 | -8.2 | ZINC000000000148 | -7.6 | ZINC000000000394 | -7 | ZINC000000000603 | -6.3 |
| ZINC000000000509 | -10.5 | ZINC000000000406 | -8.8 | ZINC000000000484 | -8.2 | ZINC000000000190 | -7.6 | ZINC000000000503 | -7 | ZINC000000000226 | -6.2 |
| ZINC000000000572 | -10.4 | ZINC000000000433 | -8.8 | ZINC000000000594 | -8.2 | ZINC000000000352 | -7.6 | ZINC000000000591 | -7 | ZINC000000000241 | -6.2 |
| ZINC000000000590 | -10.4 | ZINC000000000544 | -8.8 | ZINC000000000076 | -8.1 | ZINC000000000365 | -7.6 | ZINC000000000044 | -6.9 | ZINC000000000376 | -6.2 |
| ZINC000000000347 | -10.3 | ZINC000000000561 | -8.8 | ZINC000000000127 | -8.1 | ZINC000000000438 | -7.6 | ZINC000000000061 | -6.9 | ZINC000000000469 | -6.2 |
| ZINC000000000580 | -10.3 | ZINC000000000360 | -8.7 | ZINC000000000137 | -8.1 | ZINC000000000455 | -7.6 | ZINC000000000093 | -6.9 | ZINC000000000034 | -6.1 |
| ZINC000000000052 | -10.2 | ZINC000000000416 | -8.7 | ZINC000000000245 | -8.1 | ZINC000000000565 | -7.6 | ZINC000000000390 | -6.9 | ZINC000000000072 | -6.1 |
| ZINC000000000526 | -10.2 | ZINC000000000589 | -8.7 | ZINC000000000270 | -8.1 | ZINC000000000021 | -7.5 | ZINC000000000392 | -6.9 | ZINC000000000261 | -6.1 |
| ZINC000000000574 | -10.2 | ZINC000000000024 | -8.6 | ZINC000000000285 | -8.1 | ZINC000000000124 | -7.5 | ZINC000000000483 | -6.9 | ZINC000000000338 | -6.1 |
| ZINC000000000080 | -10 | ZINC000000000088 | -8.6 | ZINC000000000349 | -8.1 | ZINC000000000150 | -7.5 | ZINC000000000608 | -6.9 | ZINC000000000036 | -6 |
| ZINC000000000144 | -9.9 | ZINC000000000145 | -8.6 | ZINC000000000374 | -8.1 | ZINC000000000167 | -7.5 | ZINC000000000609 | -6.9 | ZINC000000000098 | -6 |
| ZINC000000000223 | -9.9 | ZINC000000000199 | -8.6 | ZINC000000000381 | -8.1 | ZINC000000000174 | -7.5 | ZINC000000000028 | -6.8 | ZINC000000000231 | -5.9 |
| ZINC000000000259 | -9.9 | ZINC000000000346 | -8.6 | ZINC000000000387 | -8.1 | ZINC000000000187 | -7.5 | ZINC000000000158 | -6.8 | ZINC000000000373 | -5.9 |
| ZINC000000000163 | -9.8 | ZINC000000000431 | -8.6 | ZINC000000000456 | -8.1 | ZINC000000000274 | -7.5 | ZINC000000000184 | -6.8 | ZINC000000000490 | -5.9 |
| ZINC000000000240 | -9.8 | ZINC000000000440 | -8.6 | ZINC000000000471 | -8.1 | ZINC000000000299 | -7.5 | ZINC000000000193 | -6.8 | ZINC000000000602 | -5.9 |
| ZINC000000000050 | -9.7 | ZINC000000000446 | -8.6 | ZINC000000000562 | -8.1 | ZINC000000000350 | -7.5 | ZINC000000000334 | -6.8 | ZINC000000000018 | -5.8 |
| ZINC000000000315 | -9.7 | ZINC000000000478 | -8.6 | ZINC000000000605 | -8.1 | ZINC000000000389 | -7.5 | ZINC000000000436 | -6.8 | ZINC000000000194 | -5.8 |
| ZINC000000000340 | -9.7 | ZINC000000000566 | -8.6 | ZINC000000000607 | -8.1 | ZINC000000000393 | -7.5 | ZINC000000000450 | -6.8 | ZINC000000000356 | -5.7 |
| ZINC000000000391 | -9.6 | ZINC000000000581 | -8.6 | ZINC000000000161 | -8 | ZINC000000000437 | -7.5 | ZINC000000000452 | -6.8 | ZINC000000000558 | -5.7 |
| ZINC000000000154 | -9.5 | ZINC000000000030 | -8.5 | ZINC000000000200 | -8 | ZINC000000000444 | -7.5 | ZINC000000000130 | -6.7 | ZINC000000000045 | -5.6 |
| ZINC000000000449 | -9.5 | ZINC000000000039 | -8.5 | ZINC000000000246 | -8 | ZINC000000000542 | -7.5 | ZINC000000000166 | -6.7 | ZINC000000000510 | -5.5 |
| ZINC000000000583 | -9.5 | ZINC000000000056 | -8.5 | ZINC000000000287 | -8 | ZINC000000000547 | -7.5 | ZINC000000000232 | -6.7 | ZINC000000000567 | -5.5 |
| ZINC000000000149 | -9.4 | ZINC000000000192 | -8.5 | ZINC000000000367 | -8 | ZINC000000000586 | -7.5 | ZINC000000000265 | -6.7 | ZINC000000000560 | -5.3 |
| ZINC000000000179 | -9.4 | ZINC000000000214 | -8.5 | ZINC000000000404 | -8 | ZINC000000000017 | -7.4 | ZINC000000000290 | -6.7 | ZINC000000000571 | -5.3 |
| ZINC000000000181 | -9.4 | ZINC000000000243 | -8.5 | ZINC000000000500 | -8 | ZINC000000000035 | -7.4 | ZINC000000000451 | -6.7 | ZINC000000000378 | -5 |
| ZINC000000000251 | -9.4 | ZINC000000000250 | -8.5 | ZINC000000000527 | -8 | ZINC000000000051 | -7.4 | ZINC000000000458 | -6.7 | ZINC000000000345 | -4.9 |
| ZINC000000000142 | -9.3 | ZINC000000000289 | -8.5 | ZINC000000000604 | -8 | ZINC000000000140 | -7.4 | ZINC000000000465 | -6.7 | ZINC000000000267 | -4.8 |
| ZINC000000000217 | -9.3 | ZINC000000000297 | -8.5 | ZINC000000000010 | -7.9 | ZINC000000000276 | -7.4 | ZINC000000000495 | -6.7 | ZINC000000000411 | -4.8 |
| ZINC000000000410 | -9.3 | ZINC000000000316 | -8.5 | ZINC000000000031 | -7.9 | ZINC000000000288 | -7.4 | ZINC000000000552 | -6.7 | ZINC000000000015 | -4.7 |
| ZINC000000000424 | -9.3 | ZINC000000000318 | -8.5 | ZINC000000000054 | -7.9 | ZINC000000000294 | -7.4 | ZINC000000000048 | -6.6 | ZINC000000000593 | -4.7 |
| ZINC000000000474 | -9.3 | ZINC000000000447 | -8.5 | ZINC000000000066 | -7.9 | ZINC000000000296 | -7.4 | ZINC000000000099 | -6.6 | ZINC000000000083 | -4.5 |
| ZINC000000000012 | -9.2 | ZINC000000000529 | -8.5 | ZINC000000000078 | -7.9 | ZINC000000000357 | -7.4 | ZINC000000000215 | -6.6 | ZINC000000000573 | -4.2 |
| ZINC000000000169 | -9.2 | ZINC000000000555 | -8.5 | ZINC000000000298 | -7.9 | ZINC000000000371 | -7.4 | ZINC000000000311 | -6.6 | ZINC000000000457 | -3.7 |
| ZINC000000000364 | -9.2 | ZINC000000000587 | -8.5 | ZINC000000000385 | -7.9 | ZINC000000000384 | -7.4 | ZINC000000000351 | -6.6 |  |  |
| ZINC000000000480 | -9.2 | ZINC000000000085 | -8.4 | ZINC000000000403 | -7.9 | ZINC000000000413 | -7.4 | ZINC000000000417 | -6.6 |  |  |
| ZINC000000000485 | -9.2 | ZINC000000000095 | -8.4 | ZINC000000000407 | -7.9 | ZINC000000000426 | -7.4 | ZINC000000000425 | -6.6 |  |  |
| ZINC000000000596 | -9.2 | ZINC000000000096 | -8.4 | ZINC000000000453 | -7.9 | ZINC000000000546 | -7.4 | ZINC000000000464 | -6.6 |  |  |
| ZINC000000000077 | -9.1 | ZINC000000000189 | -8.4 | ZINC000000000479 | -7.9 | ZINC000000000551 | -7.4 | ZINC000000000540 | -6.6 |  |  |
| ZINC000000000282 | -9.1 | ZINC000000000283 | -8.4 | ZINC000000000499 | -7.9 | ZINC000000000557 | -7.4 | ZINC000000000135 | -6.5 |  |  |
| ZINC000000000293 | -9.1 | ZINC000000000284 | -8.4 | ZINC000000000534 | -7.9 | ZINC000000000023 | -7.3 | ZINC000000000136 | -6.5 |  |  |
| ZINC000000000372 | -9.1 | ZINC000000000348 | -8.4 | ZINC000000000011 | -7.8 | ZINC000000000196 | -7.3 | ZINC000000000221 | -6.5 |  |  |
| ZINC000000000582 | -9.1 | ZINC000000000435 | -8.4 | ZINC000000000063 | -7.8 | ZINC000000000401 | -7.3 | ZINC000000000441 | -6.5 |  |  |
| ZINC000000000585 | -9.1 | ZINC000000000473 | -8.4 | ZINC000000000075 | -7.8 | ZINC000000000430 | -7.3 | ZINC000000000507 | -6.5 |  |  |
| ZINC000000000070 | -9 | ZINC000000000488 | -8.4 | ZINC000000000128 | -7.8 | ZINC000000000164 | -7.2 | ZINC000000000513 | -6.5 |  |  |
| ZINC000000000071 | -9 | ZINC000000000523 | -8.4 | ZINC000000000133 | -7.8 | ZINC000000000216 | -7.2 | ZINC000000000539 | -6.5 |  |  |
| ZINC000000000198 | -9 | ZINC000000000595 | -8.4 | ZINC000000000186 | -7.8 | ZINC000000000257 | -7.2 | ZINC000000000209 | -6.4 |  |  |
| ZINC000000000255 | -9 | ZINC000000000153 | -8.3 | ZINC000000000234 | -7.8 | ZINC000000000272 | -7.2 | ZINC000000000266 | -6.4 |  |  |
| ZINC000000000305 | -9 | ZINC000000000171 | -8.3 | ZINC000000000235 | -7.8 | ZINC000000000472 | -7.2 | ZINC000000000273 | -6.4 |  |  |
| ZINC000000000323 | -9 | ZINC000000000176 | -8.3 | ZINC000000000268 | -7.8 | ZINC000000000541 | -7.2 | ZINC000000000353 | -6.4 |  |  |
| ZINC000000000363 | -9 | ZINC000000000368 | -8.3 | ZINC000000000300 | -7.8 | ZINC000000000550 | -7.2 | ZINC000000000486 | -6.4 |  |  |
| ZINC000000000380 | -9 | ZINC000000000428 | -8.3 | ZINC000000000517 | -7.8 | ZINC000000000007 | -7.1 | ZINC000000000556 | -6.4 |  |  |
| ZINC000000000508 | -9 | ZINC000000000432 | -8.3 | ZINC000000000521 | -7.8 | ZINC000000000125 | -7.1 | ZINC000000000053 | -6.3 |  |  |
| ZINC000000000172 | -8.9 | ZINC000000000434 | -8.3 | ZINC000000000242 | -7.7 | ZINC000000000131 | -7.1 | ZINC000000000092 | -6.3 |  |  |
| ZINC000000000188 | -8.9 | ZINC000000000538 | -8.3 | ZINC000000000412 | -7.7 | ZINC000000000254 | -7.1 | ZINC000000000178 | -6.3 |  |  |
| ZINC000000000361 | -8.9 | ZINC000000000043 | -8.2 | ZINC000000000460 | -7.7 | ZINC000000000375 | -7.1 | ZINC000000000325 | -6.3 |  |  |
| ZINC000000000505 | -8.9 | ZINC000000000132 | -8.2 | ZINC000000000494 | -7.7 | ZINC000000000506 | -7.1 | ZINC000000000327 | -6.3 |  |  |
| ZINC000000000575 | -8.9 | ZINC000000000253 | -8.2 | ZINC000000000570 | -7.7 | ZINC000000000022 | -7 | ZINC000000000467 | -6.3 |  |  |
| ZINC000000000073 | -8.8 | ZINC000000000359 | -8.2 | ZINC000000000599 | -7.7 | ZINC000000000038 | -7 | ZINC000000000487 | -6.3 |  |  |

The Affinity of ligands from luteolin mol2 file docking with CXCL8

| name | Affinity (kcal/mol) | name | Affinity (kcal/mol) | name | Affinity (kcal/mol) | name | Affinity (kcal/mol) | name | Affinity (kcal/mol) | name | Affinity (kcal/mol) |
| --- | --- | --- | --- | --- | --- | --- | --- | --- | --- | --- | --- |
| ZINC000000000163 | -7.6 | ZINC000000000347 | -6.5 | ZINC000000000172 | -6 | ZINC000000000452 | -5.7 | ZINC000000000604 | -5.4 | ZINC000000000451 | -4.9 |
| ZINC000000000504 | -7.6 | ZINC000000000349 | -6.5 | ZINC000000000188 | -6 | ZINC000000000523 | -5.7 | ZINC000000000023 | -5.3 | ZINC000000000513 | -4.9 |
| ZINC000000000144 | -7.4 | ZINC000000000446 | -6.5 | ZINC000000000192 | -6 | ZINC000000000552 | -5.7 | ZINC000000000028 | -5.3 | ZINC000000000559 | -4.9 |
| ZINC000000000572 | -7.4 | ZINC000000000505 | -6.5 | ZINC000000000214 | -6 | ZINC000000000562 | -5.7 | ZINC000000000038 | -5.3 | ZINC000000000034 | -4.8 |
| ZINC000000000574 | -7.4 | ZINC000000000526 | -6.5 | ZINC000000000240 | -6 | ZINC000000000603 | -5.7 | ZINC000000000100 | -5.3 | ZINC000000000131 | -4.8 |
| ZINC000000000509 | -7.3 | ZINC000000000575 | -6.5 | ZINC000000000274 | -6 | ZINC000000000608 | -5.7 | ZINC000000000164 | -5.3 | ZINC000000000174 | -4.8 |
| ZINC000000000152 | -7.2 | ZINC000000000043 | -6.4 | ZINC000000000350 | -6 | ZINC000000000021 | -5.6 | ZINC000000000186 | -5.3 | ZINC000000000194 | -4.8 |
| ZINC000000000254 | -7.2 | ZINC000000000052 | -6.4 | ZINC000000000372 | -6 | ZINC000000000031 | -5.6 | ZINC000000000209 | -5.3 | ZINC000000000235 | -4.8 |
| ZINC000000000340 | -7.2 | ZINC000000000176 | -6.4 | ZINC000000000384 | -6 | ZINC000000000075 | -5.6 | ZINC000000000232 | -5.3 | ZINC000000000273 | -4.8 |
| ZINC000000000410 | -7.2 | ZINC000000000217 | -6.4 | ZINC000000000416 | -6 | ZINC000000000124 | -5.6 | ZINC000000000234 | -5.3 | ZINC000000000231 | -4.7 |
| ZINC000000000123 | -7.1 | ZINC000000000283 | -6.4 | ZINC000000000455 | -6 | ZINC000000000148 | -5.6 | ZINC000000000276 | -5.3 | ZINC000000000373 | -4.7 |
| ZINC000000000145 | -7.1 | ZINC000000000352 | -6.4 | ZINC000000000456 | -6 | ZINC000000000153 | -5.6 | ZINC000000000311 | -5.3 | ZINC000000000425 | -4.7 |
| ZINC000000000181 | -7.1 | ZINC000000000363 | -6.4 | ZINC000000000500 | -6 | ZINC000000000216 | -5.6 | ZINC000000000371 | -5.3 | ZINC000000000599 | -4.7 |
| ZINC000000000440 | -7.1 | ZINC000000000393 | -6.4 | ZINC000000000561 | -6 | ZINC000000000242 | -5.6 | ZINC000000000388 | -5.3 | ZINC000000000241 | -4.6 |
| ZINC000000000583 | -7.1 | ZINC000000000595 | -6.4 | ZINC000000000582 | -6 | ZINC000000000245 | -5.6 | ZINC000000000401 | -5.3 | ZINC000000000529 | -4.6 |
| ZINC000000000142 | -7 | ZINC000000000078 | -6.3 | ZINC000000000044 | -5.9 | ZINC000000000255 | -5.6 | ZINC000000000417 | -5.3 | ZINC000000000018 | -4.5 |
| ZINC000000000433 | -7 | ZINC000000000243 | -6.3 | ZINC000000000137 | -5.9 | ZINC000000000298 | -5.6 | ZINC000000000434 | -5.3 | ZINC000000000098 | -4.5 |
| ZINC000000000154 | -6.9 | ZINC000000000293 | -6.3 | ZINC000000000140 | -5.9 | ZINC000000000385 | -5.6 | ZINC000000000436 | -5.3 | ZINC000000000136 | -4.5 |
| ZINC000000000251 | -6.9 | ZINC000000000370 | -6.3 | ZINC000000000189 | -5.9 | ZINC000000000437 | -5.6 | ZINC000000000465 | -5.3 | ZINC000000000376 | -4.5 |
| ZINC000000000253 | -6.9 | ZINC000000000432 | -6.3 | ZINC000000000246 | -5.9 | ZINC000000000441 | -5.6 | ZINC000000000467 | -5.3 | ZINC000000000558 | -4.5 |
| ZINC000000000289 | -6.9 | ZINC000000000435 | -6.3 | ZINC000000000287 | -5.9 | ZINC000000000464 | -5.6 | ZINC000000000539 | -5.3 | ZINC000000000135 | -4.4 |
| ZINC000000000315 | -6.9 | ZINC000000000447 | -6.3 | ZINC000000000368 | -5.9 | ZINC000000000488 | -5.6 | ZINC000000000609 | -5.3 | ZINC000000000571 | -4.4 |
| ZINC000000000554 | -6.9 | ZINC000000000463 | -6.3 | ZINC000000000387 | -5.9 | ZINC000000000503 | -5.6 | ZINC000000000007 | -5.2 | ZINC000000000045 | -4.3 |
| ZINC000000000024 | -6.8 | ZINC000000000544 | -6.3 | ZINC000000000471 | -5.9 | ZINC000000000527 | -5.6 | ZINC000000000036 | -5.2 | ZINC000000000345 | -4.3 |
| ZINC000000000030 | -6.8 | ZINC000000000546 | -6.3 | ZINC000000000485 | -5.9 | ZINC000000000547 | -5.6 | ZINC000000000158 | -5.2 | ZINC000000000378 | -4.3 |
| ZINC000000000071 | -6.8 | ZINC000000000010 | -6.2 | ZINC000000000506 | -5.9 | ZINC000000000556 | -5.6 | ZINC000000000178 | -5.2 | ZINC000000000593 | -4.3 |
| ZINC000000000080 | -6.8 | ZINC000000000050 | -6.2 | ZINC000000000587 | -5.9 | ZINC000000000570 | -5.6 | ZINC000000000226 | -5.2 | ZINC000000000267 | -4.2 |
| ZINC000000000199 | -6.8 | ZINC000000000076 | -6.2 | ZINC000000000035 | -5.8 | ZINC000000000591 | -5.6 | ZINC000000000383 | -5.2 | ZINC000000000560 | -4.2 |
| ZINC000000000259 | -6.8 | ZINC000000000200 | -6.2 | ZINC000000000051 | -5.8 | ZINC000000000022 | -5.5 | ZINC000000000390 | -5.2 | ZINC000000000567 | -4.1 |
| ZINC000000000305 | -6.8 | ZINC000000000277 | -6.2 | ZINC000000000095 | -5.8 | ZINC000000000048 | -5.5 | ZINC000000000430 | -5.2 | ZINC000000000510 | -4 |
| ZINC000000000449 | -6.8 | ZINC000000000316 | -6.2 | ZINC000000000122 | -5.8 | ZINC000000000093 | -5.5 | ZINC000000000486 | -5.2 | ZINC000000000015 | -3.8 |
| ZINC000000000474 | -6.8 | ZINC000000000318 | -6.2 | ZINC000000000125 | -5.8 | ZINC000000000099 | -5.5 | ZINC000000000053 | -5.1 | ZINC000000000411 | -3.7 |
| ZINC000000000580 | -6.8 | ZINC000000000348 | -6.2 | ZINC000000000128 | -5.8 | ZINC000000000127 | -5.5 | ZINC000000000061 | -5.1 | ZINC000000000083 | -3.3 |
| ZINC000000000585 | -6.8 | ZINC000000000359 | -6.2 | ZINC000000000187 | -5.8 | ZINC000000000130 | -5.5 | ZINC000000000261 | -5.1 | ZINC000000000457 | -3.2 |
| ZINC000000000605 | -6.8 | ZINC000000000428 | -6.2 | ZINC000000000196 | -5.8 | ZINC000000000133 | -5.5 | ZINC000000000288 | -5.1 | ZINC000000000573 | -3.2 |
| ZINC000000000077 | -6.7 | ZINC000000000480 | -6.2 | ZINC000000000271 | -5.8 | ZINC000000000150 | -5.5 | ZINC000000000325 | -5.1 |  |  |
| ZINC000000000132 | -6.7 | ZINC000000000538 | -6.2 | ZINC000000000282 | -5.8 | ZINC000000000184 | -5.5 | ZINC000000000327 | -5.1 |  |  |
| ZINC000000000149 | -6.7 | ZINC000000000565 | -6.2 | ZINC000000000296 | -5.8 | ZINC000000000424 | -5.5 | ZINC000000000338 | -5.1 |  |  |
| ZINC000000000169 | -6.7 | ZINC000000000589 | -6.2 | ZINC000000000334 | -5.8 | ZINC000000000473 | -5.5 | ZINC000000000353 | -5.1 |  |  |
| ZINC000000000364 | -6.7 | ZINC000000000594 | -6.2 | ZINC000000000360 | -5.8 | ZINC000000000484 | -5.5 | ZINC000000000356 | -5.1 |  |  |
| ZINC000000000403 | -6.7 | ZINC000000000012 | -6.1 | ZINC000000000375 | -5.8 | ZINC000000000517 | -5.5 | ZINC000000000392 | -5.1 |  |  |
| ZINC000000000460 | -6.7 | ZINC000000000054 | -6.1 | ZINC000000000380 | -5.8 | ZINC000000000534 | -5.5 | ZINC000000000413 | -5.1 |  |  |
| ZINC000000000478 | -6.7 | ZINC000000000066 | -6.1 | ZINC000000000389 | -5.8 | ZINC000000000542 | -5.5 | ZINC000000000450 | -5.1 |  |  |
| ZINC000000000479 | -6.7 | ZINC000000000073 | -6.1 | ZINC000000000404 | -5.8 | ZINC000000000550 | -5.5 | ZINC000000000469 | -5.1 |  |  |
| ZINC000000000566 | -6.7 | ZINC000000000250 | -6.1 | ZINC000000000407 | -5.8 | ZINC000000000551 | -5.5 | ZINC000000000472 | -5.1 |  |  |
| ZINC000000000590 | -6.7 | ZINC000000000285 | -6.1 | ZINC000000000438 | -5.8 | ZINC000000000586 | -5.5 | ZINC000000000483 | -5.1 |  |  |
| ZINC000000000161 | -6.6 | ZINC000000000294 | -6.1 | ZINC000000000521 | -5.8 | ZINC000000000011 | -5.4 | ZINC000000000490 | -5.1 |  |  |
| ZINC000000000179 | -6.6 | ZINC000000000297 | -6.1 | ZINC000000000555 | -5.8 | ZINC000000000017 | -5.4 | ZINC000000000491 | -5.1 |  |  |
| ZINC000000000223 | -6.6 | ZINC000000000299 | -6.1 | ZINC000000000557 | -5.8 | ZINC000000000039 | -5.4 | ZINC000000000495 | -5.1 |  |  |
| ZINC000000000284 | -6.6 | ZINC000000000365 | -6.1 | ZINC000000000581 | -5.8 | ZINC000000000063 | -5.4 | ZINC000000000507 | -5.1 |  |  |
| ZINC000000000323 | -6.6 | ZINC000000000381 | -6.1 | ZINC000000000056 | -5.7 | ZINC000000000072 | -5.4 | ZINC000000000092 | -5 |  |  |
| ZINC000000000346 | -6.6 | ZINC000000000406 | -6.1 | ZINC000000000171 | -5.7 | ZINC000000000166 | -5.4 | ZINC000000000167 | -5 |  |  |
| ZINC000000000361 | -6.6 | ZINC000000000444 | -6.1 | ZINC000000000190 | -5.7 | ZINC000000000257 | -5.4 | ZINC000000000290 | -5 |  |  |
| ZINC000000000391 | -6.6 | ZINC000000000499 | -6.1 | ZINC000000000215 | -5.7 | ZINC000000000272 | -5.4 | ZINC000000000394 | -5 |  |  |
| ZINC000000000431 | -6.6 | ZINC000000000541 | -6.1 | ZINC000000000268 | -5.7 | ZINC000000000397 | -5.4 | ZINC000000000458 | -5 |  |  |
| ZINC000000000453 | -6.6 | ZINC000000000596 | -6.1 | ZINC000000000270 | -5.7 | ZINC000000000412 | -5.4 | ZINC000000000602 | -5 |  |  |
| ZINC000000000508 | -6.6 | ZINC000000000607 | -6.1 | ZINC000000000300 | -5.7 | ZINC000000000426 | -5.4 | ZINC000000000193 | -4.9 |  |  |
| ZINC000000000070 | -6.5 | ZINC000000000085 | -6 | ZINC000000000357 | -5.7 | ZINC000000000487 | -5.4 | ZINC000000000221 | -4.9 |  |  |
| ZINC000000000198 | -6.5 | ZINC000000000088 | -6 | ZINC000000000367 | -5.7 | ZINC000000000494 | -5.4 | ZINC000000000266 | -4.9 |  |  |
| ZINC000000000265 | -6.5 | ZINC000000000096 | -6 | ZINC000000000374 | -5.7 | ZINC000000000540 | -5.4 | ZINC000000000351 | -4.9 |  |  |

The Affinity of ligands from luteolin mol2 file docking with IL-6

| name | Affinity (kcal/mol) | name | Affinity (kcal/mol) | name | Affinity (kcal/mol) | name | Affinity (kcal/mol) | name | Affinity (kcal/mol) | name | Affinity (kcal/mol) |
| --- | --- | --- | --- | --- | --- | --- | --- | --- | --- | --- | --- |
| ZINC000000000142 | -7.7 | ZINC000000000596 | -6.3 | ZINC000000000323 | -5.9 | ZINC000000000546 | -5.6 | ZINC000000000035 | -5.2 | ZINC000000000351 | -4.9 |
| ZINC000000000253 | -7.3 | ZINC000000000056 | -6.2 | ZINC000000000334 | -5.9 | ZINC000000000550 | -5.6 | ZINC000000000072 | -5.2 | ZINC000000000413 | -4.9 |
| ZINC000000000574 | -7.2 | ZINC000000000066 | -6.2 | ZINC000000000350 | -5.9 | ZINC000000000551 | -5.6 | ZINC000000000088 | -5.2 | ZINC000000000450 | -4.9 |
| ZINC000000000080 | -7.1 | ZINC000000000073 | -6.2 | ZINC000000000357 | -5.9 | ZINC000000000562 | -5.6 | ZINC000000000174 | -5.2 | ZINC000000000539 | -4.9 |
| ZINC000000000152 | -7.1 | ZINC000000000100 | -6.2 | ZINC000000000360 | -5.9 | ZINC000000000566 | -5.6 | ZINC000000000273 | -5.2 | ZINC000000000135 | -4.8 |
| ZINC000000000554 | -7.1 | ZINC000000000176 | -6.2 | ZINC000000000394 | -5.9 | ZINC000000000604 | -5.6 | ZINC000000000298 | -5.2 | ZINC000000000221 | -4.8 |
| ZINC000000000572 | -7 | ZINC000000000179 | -6.2 | ZINC000000000397 | -5.9 | ZINC000000000031 | -5.5 | ZINC000000000311 | -5.2 | ZINC000000000458 | -4.8 |
| ZINC000000000071 | -6.9 | ZINC000000000187 | -6.2 | ZINC000000000430 | -5.9 | ZINC000000000122 | -5.5 | ZINC000000000327 | -5.2 | ZINC000000000490 | -4.8 |
| ZINC000000000259 | -6.9 | ZINC000000000189 | -6.2 | ZINC000000000478 | -5.9 | ZINC000000000125 | -5.5 | ZINC000000000365 | -5.2 | ZINC000000000036 | -4.7 |
| ZINC000000000391 | -6.9 | ZINC000000000198 | -6.2 | ZINC000000000483 | -5.9 | ZINC000000000153 | -5.5 | ZINC000000000376 | -5.2 | ZINC000000000158 | -4.7 |
| ZINC000000000580 | -6.9 | ZINC000000000240 | -6.2 | ZINC000000000589 | -5.9 | ZINC000000000184 | -5.5 | ZINC000000000389 | -5.2 | ZINC000000000338 | -4.7 |
| ZINC000000000024 | -6.8 | ZINC000000000270 | -6.2 | ZINC000000000594 | -5.9 | ZINC000000000242 | -5.5 | ZINC000000000425 | -5.2 | ZINC000000000353 | -4.7 |
| ZINC000000000123 | -6.8 | ZINC000000000315 | -6.2 | ZINC000000000010 | -5.8 | ZINC000000000257 | -5.5 | ZINC000000000469 | -5.2 | ZINC000000000602 | -4.7 |
| ZINC000000000410 | -6.8 | ZINC000000000316 | -6.2 | ZINC000000000011 | -5.8 | ZINC000000000268 | -5.5 | ZINC000000000494 | -5.2 | ZINC000000000178 | -4.6 |
| ZINC000000000431 | -6.8 | ZINC000000000318 | -6.2 | ZINC000000000030 | -5.8 | ZINC000000000271 | -5.5 | ZINC000000000495 | -5.2 | ZINC000000000325 | -4.6 |
| ZINC000000000447 | -6.8 | ZINC000000000403 | -6.2 | ZINC000000000128 | -5.8 | ZINC000000000299 | -5.5 | ZINC000000000503 | -5.2 | ZINC000000000373 | -4.6 |
| ZINC000000000480 | -6.8 | ZINC000000000404 | -6.2 | ZINC000000000164 | -5.8 | ZINC000000000390 | -5.5 | ZINC000000000540 | -5.2 | ZINC000000000487 | -4.6 |
| ZINC000000000509 | -6.8 | ZINC000000000488 | -6.2 | ZINC000000000192 | -5.8 | ZINC000000000393 | -5.5 | ZINC000000000609 | -5.2 | ZINC000000000558 | -4.6 |
| ZINC000000000526 | -6.8 | ZINC000000000534 | -6.2 | ZINC000000000196 | -5.8 | ZINC000000000484 | -5.5 | ZINC000000000018 | -5.1 | ZINC000000000092 | -4.5 |
| ZINC000000000575 | -6.8 | ZINC000000000557 | -6.2 | ZINC000000000223 | -5.8 | ZINC000000000608 | -5.5 | ZINC000000000048 | -5.1 | ZINC000000000356 | -4.5 |
| ZINC000000000583 | -6.8 | ZINC000000000043 | -6.1 | ZINC000000000245 | -5.8 | ZINC000000000017 | -5.4 | ZINC000000000130 | -5.1 | ZINC000000000510 | -4.5 |
| ZINC000000000070 | -6.7 | ZINC000000000167 | -6.1 | ZINC000000000250 | -5.8 | ZINC000000000054 | -5.4 | ZINC000000000136 | -5.1 | ZINC000000000513 | -4.5 |
| ZINC000000000368 | -6.7 | ZINC000000000199 | -6.1 | ZINC000000000288 | -5.8 | ZINC000000000063 | -5.4 | ZINC000000000148 | -5.1 | ZINC000000000567 | -4.5 |
| ZINC000000000370 | -6.7 | ZINC000000000217 | -6.1 | ZINC000000000289 | -5.8 | ZINC000000000096 | -5.4 | ZINC000000000215 | -5.1 | ZINC000000000560 | -4.4 |
| ZINC000000000432 | -6.7 | ZINC000000000283 | -6.1 | ZINC000000000300 | -5.8 | ZINC000000000098 | -5.4 | ZINC000000000241 | -5.1 | ZINC000000000571 | -4.4 |
| ZINC000000000485 | -6.7 | ZINC000000000297 | -6.1 | ZINC000000000406 | -5.8 | ZINC000000000133 | -5.4 | ZINC000000000246 | -5.1 | ZINC000000000411 | -4.3 |
| ZINC000000000504 | -6.7 | ZINC000000000374 | -6.1 | ZINC000000000407 | -5.8 | ZINC000000000137 | -5.4 | ZINC000000000276 | -5.1 | ZINC000000000045 | -4.2 |
| ZINC000000000052 | -6.6 | ZINC000000000416 | -6.1 | ZINC000000000446 | -5.8 | ZINC000000000150 | -5.4 | ZINC000000000426 | -5.1 | ZINC000000000267 | -4.1 |
| ZINC000000000077 | -6.6 | ZINC000000000500 | -6.1 | ZINC000000000456 | -5.8 | ZINC000000000186 | -5.4 | ZINC000000000464 | -5.1 | ZINC000000000345 | -4.1 |
| ZINC000000000149 | -6.6 | ZINC000000000581 | -6.1 | ZINC000000000471 | -5.8 | ZINC000000000190 | -5.4 | ZINC000000000486 | -5.1 | ZINC000000000378 | -4 |
| ZINC000000000169 | -6.6 | ZINC000000000582 | -6.1 | ZINC000000000523 | -5.8 | ZINC000000000296 | -5.4 | ZINC000000000552 | -5.1 | ZINC000000000593 | -4 |
| ZINC000000000181 | -6.6 | ZINC000000000605 | -6.1 | ZINC000000000547 | -5.8 | ZINC000000000380 | -5.4 | ZINC000000000556 | -5.1 | ZINC000000000015 | -3.9 |
| ZINC000000000214 | -6.6 | ZINC000000000012 | -6 | ZINC000000000044 | -5.7 | ZINC000000000381 | -5.4 | ZINC000000000038 | -5 | ZINC000000000083 | -3.7 |
| ZINC000000000277 | -6.5 | ZINC000000000039 | -6 | ZINC000000000085 | -5.7 | ZINC000000000383 | -5.4 | ZINC000000000099 | -5 | ZINC000000000573 | -3.6 |
| ZINC000000000340 | -6.5 | ZINC000000000050 | -6 | ZINC000000000127 | -5.7 | ZINC000000000401 | -5.4 | ZINC000000000131 | -5 | ZINC000000000457 | -3.1 |
| ZINC000000000367 | -6.5 | ZINC000000000051 | -6 | ZINC000000000132 | -5.7 | ZINC000000000434 | -5.4 | ZINC000000000194 | -5 |  |  |
| ZINC000000000075 | -6.4 | ZINC000000000163 | -6 | ZINC000000000161 | -5.7 | ZINC000000000444 | -5.4 | ZINC000000000209 | -5 |  |  |
| ZINC000000000144 | -6.4 | ZINC000000000254 | -6 | ZINC000000000234 | -5.7 | ZINC000000000453 | -5.4 | ZINC000000000226 | -5 |  |  |
| ZINC000000000251 | -6.4 | ZINC000000000255 | -6 | ZINC000000000371 | -5.7 | ZINC000000000465 | -5.4 | ZINC000000000231 | -5 |  |  |
| ZINC000000000305 | -6.4 | ZINC000000000274 | -6 | ZINC000000000385 | -5.7 | ZINC000000000472 | -5.4 | ZINC000000000232 | -5 |  |  |
| ZINC000000000346 | -6.4 | ZINC000000000293 | -6 | ZINC000000000455 | -5.7 | ZINC000000000479 | -5.4 | ZINC000000000261 | -5 |  |  |
| ZINC000000000347 | -6.4 | ZINC000000000349 | -6 | ZINC000000000463 | -5.7 | ZINC000000000529 | -5.4 | ZINC000000000272 | -5 |  |  |
| ZINC000000000449 | -6.4 | ZINC000000000352 | -6 | ZINC000000000521 | -5.7 | ZINC000000000542 | -5.4 | ZINC000000000290 | -5 |  |  |
| ZINC000000000474 | -6.4 | ZINC000000000361 | -6 | ZINC000000000541 | -5.7 | ZINC000000000565 | -5.4 | ZINC000000000392 | -5 |  |  |
| ZINC000000000590 | -6.4 | ZINC000000000363 | -6 | ZINC000000000570 | -5.7 | ZINC000000000591 | -5.4 | ZINC000000000412 | -5 |  |  |
| ZINC000000000172 | -6.3 | ZINC000000000460 | -6 | ZINC000000000586 | -5.7 | ZINC000000000022 | -5.3 | ZINC000000000417 | -5 |  |  |
| ZINC000000000200 | -6.3 | ZINC000000000473 | -6 | ZINC000000000587 | -5.7 | ZINC000000000093 | -5.3 | ZINC000000000436 | -5 |  |  |
| ZINC000000000265 | -6.3 | ZINC000000000499 | -6 | ZINC000000000607 | -5.7 | ZINC000000000193 | -5.3 | ZINC000000000437 | -5 |  |  |
| ZINC000000000282 | -6.3 | ZINC000000000505 | -6 | ZINC000000000021 | -5.6 | ZINC000000000235 | -5.3 | ZINC000000000441 | -5 |  |  |
| ZINC000000000284 | -6.3 | ZINC000000000555 | -6 | ZINC000000000095 | -5.6 | ZINC000000000266 | -5.3 | ZINC000000000452 | -5 |  |  |
| ZINC000000000348 | -6.3 | ZINC000000000561 | -6 | ZINC000000000140 | -5.6 | ZINC000000000384 | -5.3 | ZINC000000000467 | -5 |  |  |
| ZINC000000000359 | -6.3 | ZINC000000000595 | -6 | ZINC000000000285 | -5.6 | ZINC000000000387 | -5.3 | ZINC000000000491 | -5 |  |  |
| ZINC000000000364 | -6.3 | ZINC000000000076 | -5.9 | ZINC000000000287 | -5.6 | ZINC000000000388 | -5.3 | ZINC000000000507 | -5 |  |  |
| ZINC000000000372 | -6.3 | ZINC000000000124 | -5.9 | ZINC000000000375 | -5.6 | ZINC000000000451 | -5.3 | ZINC000000000599 | -5 |  |  |
| ZINC000000000433 | -6.3 | ZINC000000000145 | -5.9 | ZINC000000000424 | -5.6 | ZINC000000000506 | -5.3 | ZINC000000000603 | -5 |  |  |
| ZINC000000000440 | -6.3 | ZINC000000000154 | -5.9 | ZINC000000000428 | -5.6 | ZINC000000000559 | -5.3 | ZINC000000000053 | -4.9 |  |  |
| ZINC000000000508 | -6.3 | ZINC000000000171 | -5.9 | ZINC000000000435 | -5.6 | ZINC000000000007 | -5.2 | ZINC000000000061 | -4.9 |  |  |
| ZINC000000000517 | -6.3 | ZINC000000000188 | -5.9 | ZINC000000000438 | -5.6 | ZINC000000000023 | -5.2 | ZINC000000000078 | -4.9 |  |  |
| ZINC000000000544 | -6.3 | ZINC000000000243 | -5.9 | ZINC000000000527 | -5.6 | ZINC000000000028 | -5.2 | ZINC000000000166 | -4.9 |  |  |
| ZINC000000000585 | -6.3 | ZINC000000000294 | -5.9 | ZINC000000000538 | -5.6 | ZINC000000000034 | -5.2 | ZINC000000000216 | -4.9 |  |  |

The Affinity of ligands from luteolin mol2 file docking with JUN

| name | Affinity (kcal/mol) | name | Affinity (kcal/mol) | name | Affinity (kcal/mol) | name | Affinity (kcal/mol) | name | Affinity (kcal/mol) | name | Affinity (kcal/mol) |
| --- | --- | --- | --- | --- | --- | --- | --- | --- | --- | --- | --- |
| ZINC000000000152 | -6.9 | ZINC000000000428 | -5.8 | ZINC000000000189 | -5.4 | ZINC000000000555 | -5.2 | ZINC000000000412 | -4.8 | ZINC000000000552 | -4.4 |
| ZINC000000000080 | -6.8 | ZINC000000000485 | -5.8 | ZINC000000000192 | -5.4 | ZINC000000000562 | -5.2 | ZINC000000000417 | -4.8 | ZINC000000000034 | -4.3 |
| ZINC000000000253 | -6.8 | ZINC000000000544 | -5.8 | ZINC000000000199 | -5.4 | ZINC000000000604 | -5.2 | ZINC000000000426 | -4.8 | ZINC000000000092 | -4.3 |
| ZINC000000000526 | -6.7 | ZINC000000000050 | -5.7 | ZINC000000000200 | -5.4 | ZINC000000000010 | -5.1 | ZINC000000000472 | -4.8 | ZINC000000000194 | -4.3 |
| ZINC000000000574 | -6.7 | ZINC000000000282 | -5.7 | ZINC000000000285 | -5.4 | ZINC000000000021 | -5.1 | ZINC000000000023 | -4.7 | ZINC000000000231 | -4.3 |
| ZINC000000000504 | -6.6 | ZINC000000000283 | -5.7 | ZINC000000000359 | -5.4 | ZINC000000000056 | -5.1 | ZINC000000000028 | -4.7 | ZINC000000000325 | -4.3 |
| ZINC000000000554 | -6.6 | ZINC000000000293 | -5.7 | ZINC000000000360 | -5.4 | ZINC000000000095 | -5.1 | ZINC000000000072 | -4.7 | ZINC000000000351 | -4.3 |
| ZINC000000000347 | -6.5 | ZINC000000000323 | -5.7 | ZINC000000000368 | -5.4 | ZINC000000000137 | -5.1 | ZINC000000000093 | -4.7 | ZINC000000000529 | -4.3 |
| ZINC000000000432 | -6.4 | ZINC000000000346 | -5.7 | ZINC000000000383 | -5.4 | ZINC000000000166 | -5.1 | ZINC000000000125 | -4.7 | ZINC000000000018 | -4.2 |
| ZINC000000000572 | -6.4 | ZINC000000000361 | -5.7 | ZINC000000000389 | -5.4 | ZINC000000000187 | -5.1 | ZINC000000000136 | -4.7 | ZINC000000000036 | -4.2 |
| ZINC000000000315 | -6.3 | ZINC000000000363 | -5.7 | ZINC000000000397 | -5.4 | ZINC000000000365 | -5.1 | ZINC000000000174 | -4.7 | ZINC000000000053 | -4.2 |
| ZINC000000000431 | -6.3 | ZINC000000000435 | -5.7 | ZINC000000000434 | -5.4 | ZINC000000000452 | -5.1 | ZINC000000000193 | -4.7 | ZINC000000000098 | -4.2 |
| ZINC000000000070 | -6.2 | ZINC000000000440 | -5.7 | ZINC000000000460 | -5.4 | ZINC000000000453 | -5.1 | ZINC000000000215 | -4.7 | ZINC000000000338 | -4.2 |
| ZINC000000000144 | -6.2 | ZINC000000000447 | -5.7 | ZINC000000000471 | -5.4 | ZINC000000000517 | -5.1 | ZINC000000000216 | -4.7 | ZINC000000000353 | -4.2 |
| ZINC000000000172 | -6.2 | ZINC000000000508 | -5.7 | ZINC000000000523 | -5.4 | ZINC000000000521 | -5.1 | ZINC000000000271 | -4.7 | ZINC000000000373 | -4.2 |
| ZINC000000000181 | -6.2 | ZINC000000000589 | -5.7 | ZINC000000000570 | -5.4 | ZINC000000000586 | -5.1 | ZINC000000000311 | -4.7 | ZINC000000000458 | -4.2 |
| ZINC000000000223 | -6.2 | ZINC000000000605 | -5.7 | ZINC000000000581 | -5.4 | ZINC000000000599 | -5.1 | ZINC000000000392 | -4.7 | ZINC000000000487 | -4.2 |
| ZINC000000000259 | -6.2 | ZINC000000000039 | -5.6 | ZINC000000000582 | -5.4 | ZINC000000000011 | -5 | ZINC000000000425 | -4.7 | ZINC000000000491 | -4.2 |
| ZINC000000000340 | -6.2 | ZINC000000000100 | -5.6 | ZINC000000000594 | -5.4 | ZINC000000000044 | -5 | ZINC000000000441 | -4.7 | ZINC000000000558 | -4.2 |
| ZINC000000000449 | -6.2 | ZINC000000000127 | -5.6 | ZINC000000000607 | -5.4 | ZINC000000000054 | -5 | ZINC000000000450 | -4.7 | ZINC000000000490 | -4.1 |
| ZINC000000000509 | -6.2 | ZINC000000000167 | -5.6 | ZINC000000000051 | -5.3 | ZINC000000000164 | -5 | ZINC000000000451 | -4.7 | ZINC000000000560 | -4 |
| ZINC000000000527 | -6.2 | ZINC000000000179 | -5.6 | ZINC000000000132 | -5.3 | ZINC000000000235 | -5 | ZINC000000000550 | -4.7 | ZINC000000000567 | -4 |
| ZINC000000000590 | -6.2 | ZINC000000000214 | -5.6 | ZINC000000000133 | -5.3 | ZINC000000000257 | -5 | ZINC000000000556 | -4.7 | ZINC000000000571 | -4 |
| ZINC000000000052 | -6.1 | ZINC000000000300 | -5.6 | ZINC000000000243 | -5.3 | ZINC000000000299 | -5 | ZINC000000000603 | -4.7 | ZINC000000000045 | -3.9 |
| ZINC000000000217 | -6.1 | ZINC000000000316 | -5.6 | ZINC000000000245 | -5.3 | ZINC000000000357 | -5 | ZINC000000000038 | -4.6 | ZINC000000000356 | -3.9 |
| ZINC000000000410 | -6.1 | ZINC000000000318 | -5.6 | ZINC000000000254 | -5.3 | ZINC000000000371 | -5 | ZINC000000000099 | -4.6 | ZINC000000000510 | -3.8 |
| ZINC000000000583 | -6.1 | ZINC000000000348 | -5.6 | ZINC000000000255 | -5.3 | ZINC000000000384 | -5 | ZINC000000000130 | -4.6 | ZINC000000000593 | -3.7 |
| ZINC000000000043 | -6 | ZINC000000000364 | -5.6 | ZINC000000000284 | -5.3 | ZINC000000000401 | -5 | ZINC000000000221 | -4.6 | ZINC000000000015 | -3.6 |
| ZINC000000000149 | -6 | ZINC000000000370 | -5.6 | ZINC000000000296 | -5.3 | ZINC000000000416 | -5 | ZINC000000000266 | -4.6 | ZINC000000000411 | -3.6 |
| ZINC000000000154 | -6 | ZINC000000000381 | -5.6 | ZINC000000000334 | -5.3 | ZINC000000000430 | -5 | ZINC000000000290 | -4.6 | ZINC000000000267 | -3.5 |
| ZINC000000000374 | -6 | ZINC000000000446 | -5.6 | ZINC000000000349 | -5.3 | ZINC000000000444 | -5 | ZINC000000000375 | -4.6 | ZINC000000000083 | -3.4 |
| ZINC000000000474 | -6 | ZINC000000000480 | -5.6 | ZINC000000000350 | -5.3 | ZINC000000000546 | -5 | ZINC000000000388 | -4.6 | ZINC000000000345 | -3.4 |
| ZINC000000000561 | -6 | ZINC000000000566 | -5.6 | ZINC000000000424 | -5.3 | ZINC000000000565 | -5 | ZINC000000000437 | -4.6 | ZINC000000000378 | -3.4 |
| ZINC000000000580 | -6 | ZINC000000000063 | -5.5 | ZINC000000000438 | -5.3 | ZINC000000000007 | -4.9 | ZINC000000000486 | -4.6 | ZINC000000000573 | -3 |
| ZINC000000000024 | -5.9 | ZINC000000000096 | -5.5 | ZINC000000000473 | -5.3 | ZINC000000000017 | -4.9 | ZINC000000000506 | -4.6 | ZINC000000000457 | -2.8 |
| ZINC000000000123 | -5.9 | ZINC000000000145 | -5.5 | ZINC000000000479 | -5.3 | ZINC000000000035 | -4.9 | ZINC000000000513 | -4.6 |  |  |
| ZINC000000000142 | -5.9 | ZINC000000000153 | -5.5 | ZINC000000000484 | -5.3 | ZINC000000000048 | -4.9 | ZINC000000000591 | -4.6 |  |  |
| ZINC000000000163 | -5.9 | ZINC000000000171 | -5.5 | ZINC000000000500 | -5.3 | ZINC000000000122 | -4.9 | ZINC000000000609 | -4.6 |  |  |
| ZINC000000000240 | -5.9 | ZINC000000000250 | -5.5 | ZINC000000000547 | -5.3 | ZINC000000000148 | -4.9 | ZINC000000000078 | -4.5 |  |  |
| ZINC000000000251 | -5.9 | ZINC000000000270 | -5.5 | ZINC000000000557 | -5.3 | ZINC000000000186 | -4.9 | ZINC000000000135 | -4.5 |  |  |
| ZINC000000000277 | -5.9 | ZINC000000000274 | -5.5 | ZINC000000000587 | -5.3 | ZINC000000000190 | -4.9 | ZINC000000000178 | -4.5 |  |  |
| ZINC000000000403 | -5.9 | ZINC000000000352 | -5.5 | ZINC000000000595 | -5.3 | ZINC000000000196 | -4.9 | ZINC000000000184 | -4.5 |  |  |
| ZINC000000000406 | -5.9 | ZINC000000000372 | -5.5 | ZINC000000000076 | -5.2 | ZINC000000000234 | -4.9 | ZINC000000000226 | -4.5 |  |  |
| ZINC000000000433 | -5.9 | ZINC000000000380 | -5.5 | ZINC000000000124 | -5.2 | ZINC000000000242 | -4.9 | ZINC000000000272 | -4.5 |  |  |
| ZINC000000000505 | -5.9 | ZINC000000000456 | -5.5 | ZINC000000000150 | -5.2 | ZINC000000000298 | -4.9 | ZINC000000000273 | -4.5 |  |  |
| ZINC000000000575 | -5.9 | ZINC000000000463 | -5.5 | ZINC000000000246 | -5.2 | ZINC000000000387 | -4.9 | ZINC000000000327 | -4.5 |  |  |
| ZINC000000000585 | -5.9 | ZINC000000000478 | -5.5 | ZINC000000000268 | -5.2 | ZINC000000000413 | -4.9 | ZINC000000000407 | -4.5 |  |  |
| ZINC000000000596 | -5.9 | ZINC000000000499 | -5.5 | ZINC000000000276 | -5.2 | ZINC000000000540 | -4.9 | ZINC000000000436 | -4.5 |  |  |
| ZINC000000000012 | -5.8 | ZINC000000000534 | -5.5 | ZINC000000000287 | -5.2 | ZINC000000000551 | -4.9 | ZINC000000000464 | -4.5 |  |  |
| ZINC000000000071 | -5.8 | ZINC000000000538 | -5.5 | ZINC000000000289 | -5.2 | ZINC000000000608 | -4.9 | ZINC000000000465 | -4.5 |  |  |
| ZINC000000000073 | -5.8 | ZINC000000000541 | -5.5 | ZINC000000000294 | -5.2 | ZINC000000000061 | -4.8 | ZINC000000000469 | -4.5 |  |  |
| ZINC000000000077 | -5.8 | ZINC000000000022 | -5.4 | ZINC000000000367 | -5.2 | ZINC000000000131 | -4.8 | ZINC000000000507 | -4.5 |  |  |
| ZINC000000000169 | -5.8 | ZINC000000000030 | -5.4 | ZINC000000000393 | -5.2 | ZINC000000000140 | -4.8 | ZINC000000000539 | -4.5 |  |  |
| ZINC000000000176 | -5.8 | ZINC000000000031 | -5.4 | ZINC000000000404 | -5.2 | ZINC000000000158 | -4.8 | ZINC000000000559 | -4.5 |  |  |
| ZINC000000000188 | -5.8 | ZINC000000000066 | -5.4 | ZINC000000000455 | -5.2 | ZINC000000000232 | -4.8 | ZINC000000000602 | -4.5 |  |  |
| ZINC000000000198 | -5.8 | ZINC000000000075 | -5.4 | ZINC000000000483 | -5.2 | ZINC000000000288 | -4.8 | ZINC000000000209 | -4.4 |  |  |
| ZINC000000000265 | -5.8 | ZINC000000000085 | -5.4 | ZINC000000000488 | -5.2 | ZINC000000000376 | -4.8 | ZINC000000000241 | -4.4 |  |  |
| ZINC000000000297 | -5.8 | ZINC000000000088 | -5.4 | ZINC000000000494 | -5.2 | ZINC000000000385 | -4.8 | ZINC000000000261 | -4.4 |  |  |
| ZINC000000000305 | -5.8 | ZINC000000000128 | -5.4 | ZINC000000000503 | -5.2 | ZINC000000000390 | -4.8 | ZINC000000000467 | -4.4 |  |  |
| ZINC000000000391 | -5.8 | ZINC000000000161 | -5.4 | ZINC000000000542 | -5.2 | ZINC000000000394 | -4.8 | ZINC000000000495 | -4.4 |  |  |

The Affinity of ligands from luteolin mol2 file docking with MAPK1

| name | Affinity (kcal/mol) | name | Affinity (kcal/mol) | name | Affinity (kcal/mol) | name | Affinity (kcal/mol) | name | Affinity (kcal/mol) | name | Affinity (kcal/mol) |
| --- | --- | --- | --- | --- | --- | --- | --- | --- | --- | --- | --- |
| ZINC000000000340 | -9.4 | ZINC000000000463 | -7.9 | ZINC000000000562 | -7.5 | ZINC000000000158 | -7 | ZINC000000000581 | -6.6 | ZINC000000000356 | -5.9 |
| ZINC000000000504 | -9.1 | ZINC000000000499 | -7.9 | ZINC000000000565 | -7.5 | ZINC000000000164 | -7 | ZINC000000000034 | -6.5 | ZINC000000000483 | -5.9 |
| ZINC000000000449 | -9 | ZINC000000000589 | -7.9 | ZINC000000000607 | -7.5 | ZINC000000000184 | -7 | ZINC000000000048 | -6.5 | ZINC000000000490 | -5.9 |
| ZINC000000000152 | -8.9 | ZINC000000000594 | -7.9 | ZINC000000000096 | -7.4 | ZINC000000000294 | -7 | ZINC000000000099 | -6.5 | ZINC000000000491 | -5.9 |
| ZINC000000000214 | -8.9 | ZINC000000000010 | -7.8 | ZINC000000000124 | -7.4 | ZINC000000000334 | -7 | ZINC000000000232 | -6.5 | ZINC000000000045 | -5.8 |
| ZINC000000000251 | -8.9 | ZINC000000000085 | -7.8 | ZINC000000000265 | -7.4 | ZINC000000000363 | -7 | ZINC000000000451 | -6.5 | ZINC000000000235 | -5.8 |
| ZINC000000000071 | -8.8 | ZINC000000000128 | -7.8 | ZINC000000000349 | -7.4 | ZINC000000000384 | -7 | ZINC000000000464 | -6.5 | ZINC000000000290 | -5.8 |
| ZINC000000000169 | -8.8 | ZINC000000000200 | -7.8 | ZINC000000000394 | -7.4 | ZINC000000000416 | -7 | ZINC000000000465 | -6.5 | ZINC000000000425 | -5.8 |
| ZINC000000000189 | -8.8 | ZINC000000000347 | -7.8 | ZINC000000000428 | -7.4 | ZINC000000000494 | -7 | ZINC000000000507 | -6.5 | ZINC000000000486 | -5.8 |
| ZINC000000000283 | -8.8 | ZINC000000000350 | -7.8 | ZINC000000000444 | -7.4 | ZINC000000000028 | -6.9 | ZINC000000000556 | -6.5 | ZINC000000000571 | -5.8 |
| ZINC000000000154 | -8.7 | ZINC000000000359 | -7.8 | ZINC000000000526 | -7.4 | ZINC000000000054 | -6.9 | ZINC000000000030 | -6.4 | ZINC000000000021 | -5.7 |
| ZINC000000000346 | -8.7 | ZINC000000000380 | -7.8 | ZINC000000000529 | -7.4 | ZINC000000000077 | -6.9 | ZINC000000000031 | -6.4 | ZINC000000000072 | -5.7 |
| ZINC000000000050 | -8.6 | ZINC000000000397 | -7.8 | ZINC000000000538 | -7.4 | ZINC000000000153 | -6.9 | ZINC000000000161 | -6.4 | ZINC000000000325 | -5.7 |
| ZINC000000000144 | -8.6 | ZINC000000000433 | -7.8 | ZINC000000000546 | -7.4 | ZINC000000000167 | -6.9 | ZINC000000000209 | -6.4 | ZINC000000000437 | -5.7 |
| ZINC000000000544 | -8.6 | ZINC000000000453 | -7.8 | ZINC000000000572 | -7.4 | ZINC000000000179 | -6.9 | ZINC000000000261 | -6.4 | ZINC000000000450 | -5.7 |
| ZINC000000000199 | -8.5 | ZINC000000000505 | -7.8 | ZINC000000000587 | -7.4 | ZINC000000000188 | -6.9 | ZINC000000000273 | -6.4 | ZINC000000000487 | -5.7 |
| ZINC000000000480 | -8.5 | ZINC000000000508 | -7.8 | ZINC000000000012 | -7.3 | ZINC000000000288 | -6.9 | ZINC000000000296 | -6.4 | ZINC000000000135 | -5.6 |
| ZINC000000000527 | -8.5 | ZINC000000000566 | -7.8 | ZINC000000000017 | -7.3 | ZINC000000000297 | -6.9 | ZINC000000000327 | -6.4 | ZINC000000000373 | -5.6 |
| ZINC000000000585 | -8.5 | ZINC000000000583 | -7.8 | ZINC000000000070 | -7.3 | ZINC000000000455 | -6.9 | ZINC000000000434 | -6.4 | ZINC000000000506 | -5.6 |
| ZINC000000000127 | -8.4 | ZINC000000000051 | -7.7 | ZINC000000000088 | -7.3 | ZINC000000000473 | -6.9 | ZINC000000000599 | -6.4 | ZINC000000000267 | -5.5 |
| ZINC000000000370 | -8.4 | ZINC000000000078 | -7.7 | ZINC000000000163 | -7.3 | ZINC000000000484 | -6.9 | ZINC000000000603 | -6.4 | ZINC000000000272 | -5.5 |
| ZINC000000000474 | -8.4 | ZINC000000000352 | -7.7 | ZINC000000000174 | -7.3 | ZINC000000000503 | -6.9 | ZINC000000000061 | -6.3 | ZINC000000000558 | -5.5 |
| ZINC000000000073 | -8.3 | ZINC000000000365 | -7.7 | ZINC000000000190 | -7.3 | ZINC000000000582 | -6.9 | ZINC000000000093 | -6.3 | ZINC000000000216 | -5.4 |
| ZINC000000000243 | -8.3 | ZINC000000000367 | -7.7 | ZINC000000000223 | -7.3 | ZINC000000000608 | -6.9 | ZINC000000000125 | -6.3 | ZINC000000000567 | -5.4 |
| ZINC000000000406 | -8.3 | ZINC000000000374 | -7.7 | ZINC000000000259 | -7.3 | ZINC000000000166 | -6.8 | ZINC000000000130 | -6.3 | ZINC000000000560 | -5.2 |
| ZINC000000000440 | -8.3 | ZINC000000000383 | -7.7 | ZINC000000000282 | -7.3 | ZINC000000000234 | -6.8 | ZINC000000000215 | -6.3 | ZINC000000000510 | -5.1 |
| ZINC000000000554 | -8.3 | ZINC000000000385 | -7.7 | ZINC000000000287 | -7.3 | ZINC000000000254 | -6.8 | ZINC000000000266 | -6.3 | ZINC000000000018 | -5 |
| ZINC000000000035 | -8.2 | ZINC000000000438 | -7.7 | ZINC000000000407 | -7.3 | ZINC000000000257 | -6.8 | ZINC000000000268 | -6.3 | ZINC000000000593 | -5 |
| ZINC000000000063 | -8.2 | ZINC000000000485 | -7.7 | ZINC000000000550 | -7.3 | ZINC000000000276 | -6.8 | ZINC000000000426 | -6.3 | ZINC000000000015 | -4.8 |
| ZINC000000000100 | -8.2 | ZINC000000000557 | -7.7 | ZINC000000000590 | -7.3 | ZINC000000000371 | -6.8 | ZINC000000000452 | -6.3 | ZINC000000000345 | -4.8 |
| ZINC000000000176 | -8.2 | ZINC000000000570 | -7.7 | ZINC000000000022 | -7.2 | ZINC000000000403 | -6.8 | ZINC000000000479 | -6.3 | ZINC000000000411 | -4.7 |
| ZINC000000000323 | -8.2 | ZINC000000000604 | -7.7 | ZINC000000000066 | -7.2 | ZINC000000000447 | -6.8 | ZINC000000000523 | -6.3 | ZINC000000000378 | -4.5 |
| ZINC000000000424 | -8.2 | ZINC000000000142 | -7.6 | ZINC000000000075 | -7.2 | ZINC000000000469 | -6.8 | ZINC000000000539 | -6.3 | ZINC000000000083 | -4.3 |
| ZINC000000000043 | -8.1 | ZINC000000000187 | -7.6 | ZINC000000000123 | -7.2 | ZINC000000000540 | -6.8 | ZINC000000000591 | -6.3 | ZINC000000000573 | -4.1 |
| ZINC000000000198 | -8.1 | ZINC000000000246 | -7.6 | ZINC000000000192 | -7.2 | ZINC000000000542 | -6.8 | ZINC000000000132 | -6.2 | ZINC000000000457 | -3.9 |
| ZINC000000000245 | -8.1 | ZINC000000000285 | -7.6 | ZINC000000000196 | -7.2 | ZINC000000000547 | -6.8 | ZINC000000000137 | -6.2 |  |  |
| ZINC000000000250 | -8.1 | ZINC000000000318 | -7.6 | ZINC000000000240 | -7.2 | ZINC000000000007 | -6.7 | ZINC000000000193 | -6.2 |  |  |
| ZINC000000000284 | -8.1 | ZINC000000000372 | -7.6 | ZINC000000000242 | -7.2 | ZINC000000000023 | -6.7 | ZINC000000000194 | -6.2 |  |  |
| ZINC000000000293 | -8.1 | ZINC000000000389 | -7.6 | ZINC000000000271 | -7.2 | ZINC000000000140 | -6.7 | ZINC000000000241 | -6.2 |  |  |
| ZINC000000000456 | -8.1 | ZINC000000000412 | -7.6 | ZINC000000000274 | -7.2 | ZINC000000000145 | -6.7 | ZINC000000000376 | -6.2 |  |  |
| ZINC000000000561 | -8.1 | ZINC000000000500 | -7.6 | ZINC000000000277 | -7.2 | ZINC000000000388 | -6.7 | ZINC000000000417 | -6.2 |  |  |
| ZINC000000000574 | -8.1 | ZINC000000000052 | -7.5 | ZINC000000000357 | -7.2 | ZINC000000000401 | -6.7 | ZINC000000000436 | -6.2 |  |  |
| ZINC000000000580 | -8.1 | ZINC000000000076 | -7.5 | ZINC000000000360 | -7.2 | ZINC000000000404 | -6.7 | ZINC000000000467 | -6.2 |  |  |
| ZINC000000000039 | -8 | ZINC000000000080 | -7.5 | ZINC000000000410 | -7.2 | ZINC000000000441 | -6.7 | ZINC000000000044 | -6.1 |  |  |
| ZINC000000000171 | -8 | ZINC000000000095 | -7.5 | ZINC000000000431 | -7.2 | ZINC000000000495 | -6.7 | ZINC000000000053 | -6.1 |  |  |
| ZINC000000000172 | -8 | ZINC000000000122 | -7.5 | ZINC000000000471 | -7.2 | ZINC000000000513 | -6.7 | ZINC000000000056 | -6.1 |  |  |
| ZINC000000000217 | -8 | ZINC000000000148 | -7.5 | ZINC000000000541 | -7.2 | ZINC000000000552 | -6.7 | ZINC000000000136 | -6.1 |  |  |
| ZINC000000000348 | -8 | ZINC000000000150 | -7.5 | ZINC000000000024 | -7.1 | ZINC000000000595 | -6.7 | ZINC000000000221 | -6.1 |  |  |
| ZINC000000000391 | -8 | ZINC000000000181 | -7.5 | ZINC000000000186 | -7.1 | ZINC000000000011 | -6.6 | ZINC000000000226 | -6.1 |  |  |
| ZINC000000000488 | -8 | ZINC000000000299 | -7.5 | ZINC000000000298 | -7.1 | ZINC000000000098 | -6.6 | ZINC000000000231 | -6.1 |  |  |
| ZINC000000000509 | -8 | ZINC000000000315 | -7.5 | ZINC000000000300 | -7.1 | ZINC000000000178 | -6.6 | ZINC000000000353 | -6.1 |  |  |
| ZINC000000000605 | -8 | ZINC000000000381 | -7.5 | ZINC000000000387 | -7.1 | ZINC000000000270 | -6.6 | ZINC000000000559 | -6.1 |  |  |
| ZINC000000000149 | -7.9 | ZINC000000000390 | -7.5 | ZINC000000000393 | -7.1 | ZINC000000000289 | -6.6 | ZINC000000000609 | -6.1 |  |  |
| ZINC000000000253 | -7.9 | ZINC000000000413 | -7.5 | ZINC000000000432 | -7.1 | ZINC000000000311 | -6.6 | ZINC000000000092 | -6 |  |  |
| ZINC000000000255 | -7.9 | ZINC000000000430 | -7.5 | ZINC000000000521 | -7.1 | ZINC000000000368 | -6.6 | ZINC000000000133 | -6 |  |  |
| ZINC000000000305 | -7.9 | ZINC000000000446 | -7.5 | ZINC000000000551 | -7.1 | ZINC000000000375 | -6.6 | ZINC000000000602 | -6 |  |  |
| ZINC000000000316 | -7.9 | ZINC000000000460 | -7.5 | ZINC000000000575 | -7.1 | ZINC000000000392 | -6.6 | ZINC000000000036 | -5.9 |  |  |
| ZINC000000000361 | -7.9 | ZINC000000000517 | -7.5 | ZINC000000000586 | -7.1 | ZINC000000000458 | -6.6 | ZINC000000000038 | -5.9 |  |  |
| ZINC000000000364 | -7.9 | ZINC000000000534 | -7.5 | ZINC000000000596 | -7.1 | ZINC000000000472 | -6.6 | ZINC000000000338 | -5.9 |  |  |
| ZINC000000000435 | -7.9 | ZINC000000000555 | -7.5 | ZINC000000000131 | -7 | ZINC000000000478 | -6.6 | ZINC000000000351 | -5.9 |  |  |

The Affinity of ligands from luteolin mol2 file docking with MAPK14

| name | Affinity (kcal/mol) | name | Affinity (kcal/mol) | name | Affinity (kcal/mol) | name | Affinity (kcal/mol) | name | Affinity (kcal/mol) | name | Affinity (kcal/mol) |
| --- | --- | --- | --- | --- | --- | --- | --- | --- | --- | --- | --- |
| ZINC000000000149 | -8.9 | ZINC000000000585 | -7.4 | ZINC000000000605 | -6.9 | ZINC000000000494 | -6.5 | ZINC000000000425 | -6.1 | ZINC000000000353 | -5.6 |
| ZINC000000000253 | -8.7 | ZINC000000000077 | -7.3 | ZINC000000000051 | -6.8 | ZINC000000000557 | -6.5 | ZINC000000000503 | -6.1 | ZINC000000000376 | -5.6 |
| ZINC000000000152 | -8.5 | ZINC000000000088 | -7.3 | ZINC000000000056 | -6.8 | ZINC000000000586 | -6.5 | ZINC000000000550 | -6.1 | ZINC000000000452 | -5.6 |
| ZINC000000000259 | -8.5 | ZINC000000000128 | -7.3 | ZINC000000000066 | -6.8 | ZINC000000000604 | -6.5 | ZINC000000000608 | -6.1 | ZINC000000000241 | -5.5 |
| ZINC000000000526 | -8.3 | ZINC000000000287 | -7.3 | ZINC000000000133 | -6.8 | ZINC000000000607 | -6.5 | ZINC000000000174 | -6 | ZINC000000000458 | -5.5 |
| ZINC000000000554 | -8.3 | ZINC000000000300 | -7.3 | ZINC000000000188 | -6.8 | ZINC000000000030 | -6.4 | ZINC000000000178 | -6 | ZINC000000000467 | -5.5 |
| ZINC000000000574 | -8.3 | ZINC000000000305 | -7.3 | ZINC000000000299 | -6.8 | ZINC000000000063 | -6.4 | ZINC000000000266 | -6 | ZINC000000000487 | -5.5 |
| ZINC000000000080 | -8.2 | ZINC000000000463 | -7.3 | ZINC000000000346 | -6.8 | ZINC000000000075 | -6.4 | ZINC000000000388 | -6 | ZINC000000000558 | -5.5 |
| ZINC000000000176 | -8.2 | ZINC000000000485 | -7.3 | ZINC000000000367 | -6.8 | ZINC000000000095 | -6.4 | ZINC000000000539 | -6 | ZINC000000000567 | -5.5 |
| ZINC000000000590 | -8.2 | ZINC000000000595 | -7.3 | ZINC000000000389 | -6.8 | ZINC000000000096 | -6.4 | ZINC000000000540 | -6 | ZINC000000000194 | -5.4 |
| ZINC000000000070 | -8 | ZINC000000000012 | -7.2 | ZINC000000000434 | -6.8 | ZINC000000000100 | -6.4 | ZINC000000000552 | -6 | ZINC000000000231 | -5.4 |
| ZINC000000000154 | -8 | ZINC000000000214 | -7.2 | ZINC000000000446 | -6.8 | ZINC000000000124 | -6.4 | ZINC000000000556 | -6 | ZINC000000000261 | -5.4 |
| ZINC000000000163 | -8 | ZINC000000000250 | -7.2 | ZINC000000000479 | -6.8 | ZINC000000000164 | -6.4 | ZINC000000000599 | -6 | ZINC000000000338 | -5.4 |
| ZINC000000000347 | -8 | ZINC000000000348 | -7.2 | ZINC000000000587 | -6.8 | ZINC000000000235 | -6.4 | ZINC000000000603 | -6 | ZINC000000000356 | -5.4 |
| ZINC000000000433 | -8 | ZINC000000000374 | -7.2 | ZINC000000000017 | -6.7 | ZINC000000000274 | -6.4 | ZINC000000000021 | -5.9 | ZINC000000000490 | -5.4 |
| ZINC000000000071 | -7.9 | ZINC000000000447 | -7.2 | ZINC000000000148 | -6.7 | ZINC000000000296 | -6.4 | ZINC000000000038 | -5.9 | ZINC000000000018 | -5.3 |
| ZINC000000000123 | -7.9 | ZINC000000000473 | -7.2 | ZINC000000000200 | -6.7 | ZINC000000000357 | -6.4 | ZINC000000000039 | -5.9 | ZINC000000000267 | -5.3 |
| ZINC000000000142 | -7.9 | ZINC000000000521 | -7.2 | ZINC000000000265 | -6.7 | ZINC000000000444 | -6.4 | ZINC000000000078 | -5.9 | ZINC000000000373 | -5.3 |
| ZINC000000000144 | -7.8 | ZINC000000000523 | -7.2 | ZINC000000000288 | -6.7 | ZINC000000000483 | -6.4 | ZINC000000000098 | -5.9 | ZINC000000000450 | -5.3 |
| ZINC000000000171 | -7.8 | ZINC000000000527 | -7.2 | ZINC000000000349 | -6.7 | ZINC000000000488 | -6.4 | ZINC000000000131 | -5.9 | ZINC000000000491 | -5.3 |
| ZINC000000000391 | -7.8 | ZINC000000000581 | -7.2 | ZINC000000000365 | -6.7 | ZINC000000000529 | -6.4 | ZINC000000000413 | -5.9 | ZINC000000000571 | -5.3 |
| ZINC000000000432 | -7.8 | ZINC000000000031 | -7.1 | ZINC000000000406 | -6.7 | ZINC000000000594 | -6.4 | ZINC000000000417 | -5.9 | ZINC000000000092 | -5.2 |
| ZINC000000000449 | -7.8 | ZINC000000000050 | -7.1 | ZINC000000000416 | -6.7 | ZINC000000000093 | -6.3 | ZINC000000000426 | -5.9 | ZINC000000000351 | -5.2 |
| ZINC000000000474 | -7.8 | ZINC000000000073 | -7.1 | ZINC000000000472 | -6.7 | ZINC000000000150 | -6.3 | ZINC000000000451 | -5.9 | ZINC000000000158 | -5.1 |
| ZINC000000000508 | -7.8 | ZINC000000000127 | -7.1 | ZINC000000000534 | -6.7 | ZINC000000000184 | -6.3 | ZINC000000000464 | -5.9 | ZINC000000000560 | -5.1 |
| ZINC000000000572 | -7.8 | ZINC000000000153 | -7.1 | ZINC000000000547 | -6.7 | ZINC000000000186 | -6.3 | ZINC000000000486 | -5.9 | ZINC000000000045 | -4.9 |
| ZINC000000000217 | -7.7 | ZINC000000000179 | -7.1 | ZINC000000000565 | -6.7 | ZINC000000000234 | -6.3 | ZINC000000000495 | -5.9 | ZINC000000000593 | -4.9 |
| ZINC000000000297 | -7.7 | ZINC000000000245 | -7.1 | ZINC000000000566 | -6.7 | ZINC000000000294 | -6.3 | ZINC000000000513 | -5.9 | ZINC000000000411 | -4.8 |
| ZINC000000000504 | -7.7 | ZINC000000000254 | -7.1 | ZINC000000000570 | -6.7 | ZINC000000000371 | -6.3 | ZINC000000000591 | -5.9 | ZINC000000000510 | -4.8 |
| ZINC000000000509 | -7.7 | ZINC000000000277 | -7.1 | ZINC000000000582 | -6.7 | ZINC000000000380 | -6.3 | ZINC000000000609 | -5.9 | ZINC000000000378 | -4.5 |
| ZINC000000000589 | -7.7 | ZINC000000000364 | -7.1 | ZINC000000000028 | -6.6 | ZINC000000000383 | -6.3 | ZINC000000000022 | -5.8 | ZINC000000000083 | -4.3 |
| ZINC000000000043 | -7.6 | ZINC000000000372 | -7.1 | ZINC000000000085 | -6.6 | ZINC000000000441 | -6.3 | ZINC000000000048 | -5.8 | ZINC000000000345 | -4.3 |
| ZINC000000000172 | -7.6 | ZINC000000000403 | -7.1 | ZINC000000000282 | -6.6 | ZINC000000000460 | -6.3 | ZINC000000000053 | -5.8 | ZINC000000000015 | -4.1 |
| ZINC000000000181 | -7.6 | ZINC000000000435 | -7.1 | ZINC000000000285 | -6.6 | ZINC000000000541 | -6.3 | ZINC000000000072 | -5.8 | ZINC000000000573 | -3.8 |
| ZINC000000000240 | -7.6 | ZINC000000000455 | -7.1 | ZINC000000000289 | -6.6 | ZINC000000000542 | -6.3 | ZINC000000000136 | -5.8 | ZINC000000000457 | -3.7 |
| ZINC000000000410 | -7.6 | ZINC000000000478 | -7.1 | ZINC000000000350 | -6.6 | ZINC000000000546 | -6.3 | ZINC000000000209 | -5.8 |  |  |
| ZINC000000000431 | -7.6 | ZINC000000000499 | -7.1 | ZINC000000000352 | -6.6 | ZINC000000000551 | -6.3 | ZINC000000000215 | -5.8 |  |  |
| ZINC000000000583 | -7.6 | ZINC000000000555 | -7.1 | ZINC000000000359 | -6.6 | ZINC000000000602 | -6.3 | ZINC000000000221 | -5.8 |  |  |
| ZINC000000000596 | -7.6 | ZINC000000000561 | -7.1 | ZINC000000000368 | -6.6 | ZINC000000000010 | -6.2 | ZINC000000000232 | -5.8 |  |  |
| ZINC000000000024 | -7.5 | ZINC000000000169 | -7 | ZINC000000000404 | -6.6 | ZINC000000000044 | -6.2 | ZINC000000000242 | -5.8 |  |  |
| ZINC000000000052 | -7.5 | ZINC000000000187 | -7 | ZINC000000000428 | -6.6 | ZINC000000000061 | -6.2 | ZINC000000000273 | -5.8 |  |  |
| ZINC000000000189 | -7.5 | ZINC000000000198 | -7 | ZINC000000000438 | -6.6 | ZINC000000000099 | -6.2 | ZINC000000000311 | -5.8 |  |  |
| ZINC000000000293 | -7.5 | ZINC000000000251 | -7 | ZINC000000000453 | -6.6 | ZINC000000000125 | -6.2 | ZINC000000000401 | -5.8 |  |  |
| ZINC000000000323 | -7.5 | ZINC000000000316 | -7 | ZINC000000000011 | -6.5 | ZINC000000000166 | -6.2 | ZINC000000000034 | -5.7 |  |  |
| ZINC000000000538 | -7.5 | ZINC000000000363 | -7 | ZINC000000000035 | -6.5 | ZINC000000000196 | -6.2 | ZINC000000000036 | -5.7 |  |  |
| ZINC000000000575 | -7.5 | ZINC000000000397 | -7 | ZINC000000000054 | -6.5 | ZINC000000000257 | -6.2 | ZINC000000000130 | -5.7 |  |  |
| ZINC000000000580 | -7.5 | ZINC000000000456 | -7 | ZINC000000000132 | -6.5 | ZINC000000000298 | -6.2 | ZINC000000000140 | -5.7 |  |  |
| ZINC000000000167 | -7.4 | ZINC000000000505 | -7 | ZINC000000000137 | -6.5 | ZINC000000000334 | -6.2 | ZINC000000000216 | -5.7 |  |  |
| ZINC000000000192 | -7.4 | ZINC000000000076 | -6.9 | ZINC000000000243 | -6.5 | ZINC000000000384 | -6.2 | ZINC000000000327 | -5.7 |  |  |
| ZINC000000000223 | -7.4 | ZINC000000000145 | -6.9 | ZINC000000000246 | -6.5 | ZINC000000000392 | -6.2 | ZINC000000000436 | -5.7 |  |  |
| ZINC000000000283 | -7.4 | ZINC000000000161 | -6.9 | ZINC000000000255 | -6.5 | ZINC000000000407 | -6.2 | ZINC000000000437 | -5.7 |  |  |
| ZINC000000000315 | -7.4 | ZINC000000000199 | -6.9 | ZINC000000000270 | -6.5 | ZINC000000000465 | -6.2 | ZINC000000000469 | -5.7 |  |  |
| ZINC000000000340 | -7.4 | ZINC000000000226 | -6.9 | ZINC000000000276 | -6.5 | ZINC000000000007 | -6.1 | ZINC000000000506 | -5.7 |  |  |
| ZINC000000000361 | -7.4 | ZINC000000000268 | -6.9 | ZINC000000000284 | -6.5 | ZINC000000000122 | -6.1 | ZINC000000000507 | -5.7 |  |  |
| ZINC000000000370 | -7.4 | ZINC000000000318 | -6.9 | ZINC000000000387 | -6.5 | ZINC000000000190 | -6.1 | ZINC000000000559 | -5.7 |  |  |
| ZINC000000000440 | -7.4 | ZINC000000000360 | -6.9 | ZINC000000000393 | -6.5 | ZINC000000000193 | -6.1 | ZINC000000000023 | -5.6 |  |  |
| ZINC000000000480 | -7.4 | ZINC000000000381 | -6.9 | ZINC000000000424 | -6.5 | ZINC000000000271 | -6.1 | ZINC000000000135 | -5.6 |  |  |
| ZINC000000000500 | -7.4 | ZINC000000000385 | -6.9 | ZINC000000000430 | -6.5 | ZINC000000000375 | -6.1 | ZINC000000000272 | -5.6 |  |  |
| ZINC000000000517 | -7.4 | ZINC000000000390 | -6.9 | ZINC000000000471 | -6.5 | ZINC000000000394 | -6.1 | ZINC000000000290 | -5.6 |  |  |
| ZINC000000000544 | -7.4 | ZINC000000000562 | -6.9 | ZINC000000000484 | -6.5 | ZINC000000000412 | -6.1 | ZINC000000000325 | -5.6 |  |  |

The Affinity of ligands from luteolin mol2 file docking with RB1

| name | Affinity (kcal/mol) | name | Affinity (kcal/mol) | name | Affinity (kcal/mol) | name | Affinity (kcal/mol) | name | Affinity (kcal/mol) | name | Affinity (kcal/mol) |
| --- | --- | --- | --- | --- | --- | --- | --- | --- | --- | --- | --- |
| ZINC000000000253 | -8.4 | ZINC000000000039 | -7 | ZINC000000000359 | -6.7 | ZINC000000000017 | -6.3 | ZINC000000000539 | -6 | ZINC000000000513 | -5.5 |
| ZINC000000000574 | -8.3 | ZINC000000000043 | -7 | ZINC000000000364 | -6.7 | ZINC000000000085 | -6.3 | ZINC000000000044 | -5.9 | ZINC000000000567 | -5.5 |
| ZINC000000000080 | -8.2 | ZINC000000000050 | -7 | ZINC000000000370 | -6.7 | ZINC000000000137 | -6.3 | ZINC000000000072 | -5.9 | ZINC000000000599 | -5.5 |
| ZINC000000000259 | -8.2 | ZINC000000000124 | -7 | ZINC000000000380 | -6.7 | ZINC000000000294 | -6.3 | ZINC000000000164 | -5.9 | ZINC000000000034 | -5.4 |
| ZINC000000000554 | -8.2 | ZINC000000000176 | -7 | ZINC000000000434 | -6.7 | ZINC000000000349 | -6.3 | ZINC000000000184 | -5.9 | ZINC000000000036 | -5.4 |
| ZINC000000000123 | -8.1 | ZINC000000000188 | -7 | ZINC000000000478 | -6.7 | ZINC000000000350 | -6.3 | ZINC000000000242 | -5.9 | ZINC000000000178 | -5.4 |
| ZINC000000000509 | -8 | ZINC000000000277 | -7 | ZINC000000000523 | -6.7 | ZINC000000000375 | -6.3 | ZINC000000000288 | -5.9 | ZINC000000000216 | -5.4 |
| ZINC000000000052 | -7.9 | ZINC000000000346 | -7 | ZINC000000000538 | -6.7 | ZINC000000000385 | -6.3 | ZINC000000000290 | -5.9 | ZINC000000000351 | -5.4 |
| ZINC000000000152 | -7.9 | ZINC000000000397 | -7 | ZINC000000000555 | -6.7 | ZINC000000000389 | -6.3 | ZINC000000000388 | -5.9 | ZINC000000000491 | -5.4 |
| ZINC000000000526 | -7.9 | ZINC000000000410 | -7 | ZINC000000000594 | -6.7 | ZINC000000000430 | -6.3 | ZINC000000000464 | -5.9 | ZINC000000000018 | -5.3 |
| ZINC000000000580 | -7.9 | ZINC000000000432 | -7 | ZINC000000000150 | -6.6 | ZINC000000000452 | -6.3 | ZINC000000000506 | -5.9 | ZINC000000000158 | -5.3 |
| ZINC000000000149 | -7.8 | ZINC000000000433 | -7 | ZINC000000000243 | -6.6 | ZINC000000000479 | -6.3 | ZINC000000000529 | -5.9 | ZINC000000000215 | -5.3 |
| ZINC000000000163 | -7.8 | ZINC000000000527 | -7 | ZINC000000000274 | -6.6 | ZINC000000000540 | -6.3 | ZINC000000000551 | -5.9 | ZINC000000000272 | -5.3 |
| ZINC000000000347 | -7.8 | ZINC000000000582 | -7 | ZINC000000000285 | -6.6 | ZINC000000000570 | -6.3 | ZINC000000000552 | -5.9 | ZINC000000000338 | -5.3 |
| ZINC000000000431 | -7.8 | ZINC000000000054 | -6.9 | ZINC000000000297 | -6.6 | ZINC000000000022 | -6.2 | ZINC000000000591 | -5.9 | ZINC000000000437 | -5.3 |
| ZINC000000000504 | -7.8 | ZINC000000000066 | -6.9 | ZINC000000000300 | -6.6 | ZINC000000000031 | -6.2 | ZINC000000000604 | -5.9 | ZINC000000000490 | -5.3 |
| ZINC000000000508 | -7.8 | ZINC000000000088 | -6.9 | ZINC000000000352 | -6.6 | ZINC000000000096 | -6.2 | ZINC000000000608 | -5.9 | ZINC000000000510 | -5.3 |
| ZINC000000000583 | -7.8 | ZINC000000000128 | -6.9 | ZINC000000000360 | -6.6 | ZINC000000000148 | -6.2 | ZINC000000000078 | -5.8 | ZINC000000000098 | -5.2 |
| ZINC000000000070 | -7.7 | ZINC000000000131 | -6.9 | ZINC000000000367 | -6.6 | ZINC000000000174 | -6.2 | ZINC000000000093 | -5.8 | ZINC000000000373 | -5.2 |
| ZINC000000000544 | -7.7 | ZINC000000000154 | -6.9 | ZINC000000000383 | -6.6 | ZINC000000000187 | -6.2 | ZINC000000000122 | -5.8 | ZINC000000000556 | -5.2 |
| ZINC000000000572 | -7.7 | ZINC000000000198 | -6.9 | ZINC000000000390 | -6.6 | ZINC000000000234 | -6.2 | ZINC000000000125 | -5.8 | ZINC000000000558 | -5.2 |
| ZINC000000000024 | -7.6 | ZINC000000000200 | -6.9 | ZINC000000000394 | -6.6 | ZINC000000000235 | -6.2 | ZINC000000000130 | -5.8 | ZINC000000000356 | -5.1 |
| ZINC000000000440 | -7.6 | ZINC000000000250 | -6.9 | ZINC000000000488 | -6.6 | ZINC000000000245 | -6.2 | ZINC000000000140 | -5.8 | ZINC000000000092 | -5 |
| ZINC000000000575 | -7.6 | ZINC000000000270 | -6.9 | ZINC000000000494 | -6.6 | ZINC000000000246 | -6.2 | ZINC000000000241 | -5.8 | ZINC000000000045 | -4.9 |
| ZINC000000000144 | -7.5 | ZINC000000000283 | -6.9 | ZINC000000000500 | -6.6 | ZINC000000000393 | -6.2 | ZINC000000000325 | -5.8 | ZINC000000000560 | -4.9 |
| ZINC000000000217 | -7.5 | ZINC000000000289 | -6.9 | ZINC000000000542 | -6.6 | ZINC000000000404 | -6.2 | ZINC000000000327 | -5.8 | ZINC000000000083 | -4.7 |
| ZINC000000000255 | -7.5 | ZINC000000000316 | -6.9 | ZINC000000000586 | -6.6 | ZINC000000000425 | -6.2 | ZINC000000000417 | -5.8 | ZINC000000000411 | -4.7 |
| ZINC000000000323 | -7.5 | ZINC000000000363 | -6.9 | ZINC000000000605 | -6.6 | ZINC000000000444 | -6.2 | ZINC000000000441 | -5.8 | ZINC000000000378 | -4.6 |
| ZINC000000000361 | -7.5 | ZINC000000000365 | -6.9 | ZINC000000000028 | -6.5 | ZINC000000000456 | -6.2 | ZINC000000000603 | -5.8 | ZINC000000000571 | -4.6 |
| ZINC000000000449 | -7.5 | ZINC000000000372 | -6.9 | ZINC000000000063 | -6.5 | ZINC000000000503 | -6.2 | ZINC000000000053 | -5.7 | ZINC000000000267 | -4.5 |
| ZINC000000000474 | -7.5 | ZINC000000000381 | -6.9 | ZINC000000000127 | -6.5 | ZINC000000000550 | -6.2 | ZINC000000000135 | -5.7 | ZINC000000000593 | -4.5 |
| ZINC000000000485 | -7.5 | ZINC000000000447 | -6.9 | ZINC000000000153 | -6.5 | ZINC000000000559 | -6.2 | ZINC000000000193 | -5.7 | ZINC000000000015 | -4.4 |
| ZINC000000000590 | -7.5 | ZINC000000000453 | -6.9 | ZINC000000000161 | -6.5 | ZINC000000000609 | -6.2 | ZINC000000000209 | -5.7 | ZINC000000000345 | -4.2 |
| ZINC000000000142 | -7.4 | ZINC000000000480 | -6.9 | ZINC000000000186 | -6.5 | ZINC000000000023 | -6.1 | ZINC000000000232 | -5.7 | ZINC000000000573 | -3.9 |
| ZINC000000000265 | -7.4 | ZINC000000000499 | -6.9 | ZINC000000000192 | -6.5 | ZINC000000000095 | -6.1 | ZINC000000000276 | -5.7 | ZINC000000000457 | -3.7 |
| ZINC000000000305 | -7.4 | ZINC000000000517 | -6.9 | ZINC000000000368 | -6.5 | ZINC000000000133 | -6.1 | ZINC000000000353 | -5.7 |  |  |
| ZINC000000000391 | -7.4 | ZINC000000000581 | -6.9 | ZINC000000000371 | -6.5 | ZINC000000000190 | -6.1 | ZINC000000000436 | -5.7 |  |  |
| ZINC000000000561 | -7.4 | ZINC000000000585 | -6.9 | ZINC000000000387 | -6.5 | ZINC000000000196 | -6.1 | ZINC000000000450 | -5.7 |  |  |
| ZINC000000000012 | -7.3 | ZINC000000000587 | -6.9 | ZINC000000000403 | -6.5 | ZINC000000000271 | -6.1 | ZINC000000000458 | -5.7 |  |  |
| ZINC000000000169 | -7.3 | ZINC000000000595 | -6.9 | ZINC000000000416 | -6.5 | ZINC000000000298 | -6.1 | ZINC000000000467 | -5.7 |  |  |
| ZINC000000000181 | -7.3 | ZINC000000000010 | -6.8 | ZINC000000000446 | -6.5 | ZINC000000000348 | -6.1 | ZINC000000000602 | -5.7 |  |  |
| ZINC000000000340 | -7.3 | ZINC000000000077 | -6.8 | ZINC000000000463 | -6.5 | ZINC000000000357 | -6.1 | ZINC000000000035 | -5.6 |  |  |
| ZINC000000000374 | -7.3 | ZINC000000000132 | -6.8 | ZINC000000000546 | -6.5 | ZINC000000000392 | -6.1 | ZINC000000000048 | -5.6 |  |  |
| ZINC000000000534 | -7.3 | ZINC000000000172 | -6.8 | ZINC000000000557 | -6.5 | ZINC000000000412 | -6.1 | ZINC000000000099 | -5.6 |  |  |
| ZINC000000000566 | -7.3 | ZINC000000000179 | -6.8 | ZINC000000000051 | -6.4 | ZINC000000000424 | -6.1 | ZINC000000000166 | -5.6 |  |  |
| ZINC000000000071 | -7.2 | ZINC000000000214 | -6.8 | ZINC000000000075 | -6.4 | ZINC000000000451 | -6.1 | ZINC000000000194 | -5.6 |  |  |
| ZINC000000000100 | -7.2 | ZINC000000000284 | -6.8 | ZINC000000000076 | -6.4 | ZINC000000000460 | -6.1 | ZINC000000000226 | -5.6 |  |  |
| ZINC000000000189 | -7.2 | ZINC000000000287 | -6.8 | ZINC000000000199 | -6.4 | ZINC000000000465 | -6.1 | ZINC000000000273 | -5.6 |  |  |
| ZINC000000000251 | -7.2 | ZINC000000000406 | -6.8 | ZINC000000000257 | -6.4 | ZINC000000000507 | -6.1 | ZINC000000000376 | -5.6 |  |  |
| ZINC000000000293 | -7.2 | ZINC000000000455 | -6.8 | ZINC000000000268 | -6.4 | ZINC000000000562 | -6.1 | ZINC000000000469 | -5.6 |  |  |
| ZINC000000000473 | -7.2 | ZINC000000000505 | -6.8 | ZINC000000000282 | -6.4 | ZINC000000000565 | -6.1 | ZINC000000000472 | -5.6 |  |  |
| ZINC000000000589 | -7.2 | ZINC000000000541 | -6.8 | ZINC000000000296 | -6.4 | ZINC000000000607 | -6.1 | ZINC000000000486 | -5.6 |  |  |
| ZINC000000000240 | -7.1 | ZINC000000000056 | -6.7 | ZINC000000000428 | -6.4 | ZINC000000000021 | -6 | ZINC000000000495 | -5.6 |  |  |
| ZINC000000000254 | -7.1 | ZINC000000000073 | -6.7 | ZINC000000000438 | -6.4 | ZINC000000000061 | -6 | ZINC000000000038 | -5.5 |  |  |
| ZINC000000000315 | -7.1 | ZINC000000000145 | -6.7 | ZINC000000000471 | -6.4 | ZINC000000000136 | -6 | ZINC000000000221 | -5.5 |  |  |
| ZINC000000000318 | -7.1 | ZINC000000000167 | -6.7 | ZINC000000000484 | -6.4 | ZINC000000000384 | -6 | ZINC000000000231 | -5.5 |  |  |
| ZINC000000000407 | -7.1 | ZINC000000000171 | -6.7 | ZINC000000000521 | -6.4 | ZINC000000000401 | -6 | ZINC000000000261 | -5.5 |  |  |
| ZINC000000000435 | -7.1 | ZINC000000000223 | -6.7 | ZINC000000000547 | -6.4 | ZINC000000000413 | -6 | ZINC000000000266 | -5.5 |  |  |
| ZINC000000000596 | -7.1 | ZINC000000000299 | -6.7 | ZINC000000000007 | -6.3 | ZINC000000000426 | -6 | ZINC000000000311 | -5.5 |  |  |
| ZINC000000000030 | -7 | ZINC000000000334 | -6.7 | ZINC000000000011 | -6.3 | ZINC000000000483 | -6 | ZINC000000000487 | -5.5 |  |  |

The Affinity of ligands from luteolin mol2 file docking with RELA

| name | Affinity (kcal/mol) | name | Affinity (kcal/mol) | name | Affinity (kcal/mol) | name | Affinity (kcal/mol) | name | Affinity (kcal/mol) | name | Affinity (kcal/mol) |
| --- | --- | --- | --- | --- | --- | --- | --- | --- | --- | --- | --- |
| ZINC000000000142 | -4.3 | ZINC000000000572 | -3.7 | ZINC000000000299 | -3.4 | ZINC000000000072 | -3.2 | ZINC000000000401 | -3.1 | ZINC000000000441 | -2.8 |
| ZINC000000000123 | -4.2 | ZINC000000000580 | -3.7 | ZINC000000000300 | -3.4 | ZINC000000000075 | -3.2 | ZINC000000000444 | -3.1 | ZINC000000000458 | -2.8 |
| ZINC000000000152 | -4.2 | ZINC000000000030 | -3.6 | ZINC000000000316 | -3.4 | ZINC000000000100 | -3.2 | ZINC000000000452 | -3.1 | ZINC000000000467 | -2.8 |
| ZINC000000000554 | -4.2 | ZINC000000000050 | -3.6 | ZINC000000000323 | -3.4 | ZINC000000000127 | -3.2 | ZINC000000000484 | -3.1 | ZINC000000000469 | -2.8 |
| ZINC000000000575 | -4.2 | ZINC000000000071 | -3.6 | ZINC000000000357 | -3.4 | ZINC000000000135 | -3.2 | ZINC000000000494 | -3.1 | ZINC000000000491 | -2.8 |
| ZINC000000000251 | -4.1 | ZINC000000000163 | -3.6 | ZINC000000000361 | -3.4 | ZINC000000000140 | -3.2 | ZINC000000000539 | -3.1 | ZINC000000000506 | -2.8 |
| ZINC000000000347 | -4.1 | ZINC000000000172 | -3.6 | ZINC000000000371 | -3.4 | ZINC000000000186 | -3.2 | ZINC000000000551 | -3.1 | ZINC000000000571 | -2.8 |
| ZINC000000000440 | -4.1 | ZINC000000000188 | -3.6 | ZINC000000000375 | -3.4 | ZINC000000000196 | -3.2 | ZINC000000000582 | -3.1 | ZINC000000000609 | -2.8 |
| ZINC000000000449 | -4.1 | ZINC000000000217 | -3.6 | ZINC000000000406 | -3.4 | ZINC000000000209 | -3.2 | ZINC000000000599 | -3.1 | ZINC000000000018 | -2.7 |
| ZINC000000000574 | -4.1 | ZINC000000000265 | -3.6 | ZINC000000000410 | -3.4 | ZINC000000000214 | -3.2 | ZINC000000000031 | -3 | ZINC000000000034 | -2.7 |
| ZINC000000000080 | -4 | ZINC000000000270 | -3.6 | ZINC000000000446 | -3.4 | ZINC000000000245 | -3.2 | ZINC000000000035 | -3 | ZINC000000000038 | -2.7 |
| ZINC000000000253 | -4 | ZINC000000000318 | -3.6 | ZINC000000000451 | -3.4 | ZINC000000000255 | -3.2 | ZINC000000000150 | -3 | ZINC000000000158 | -2.7 |
| ZINC000000000305 | -4 | ZINC000000000363 | -3.6 | ZINC000000000453 | -3.4 | ZINC000000000257 | -3.2 | ZINC000000000166 | -3 | ZINC000000000215 | -2.7 |
| ZINC000000000364 | -4 | ZINC000000000384 | -3.6 | ZINC000000000471 | -3.4 | ZINC000000000268 | -3.2 | ZINC000000000193 | -3 | ZINC000000000267 | -2.7 |
| ZINC000000000024 | -3.9 | ZINC000000000433 | -3.6 | ZINC000000000521 | -3.4 | ZINC000000000287 | -3.2 | ZINC000000000235 | -3 | ZINC000000000272 | -2.7 |
| ZINC000000000154 | -3.9 | ZINC000000000447 | -3.6 | ZINC000000000534 | -3.4 | ZINC000000000290 | -3.2 | ZINC000000000242 | -3 | ZINC000000000351 | -2.7 |
| ZINC000000000259 | -3.9 | ZINC000000000464 | -3.6 | ZINC000000000555 | -3.4 | ZINC000000000352 | -3.2 | ZINC000000000273 | -3 | ZINC000000000356 | -2.7 |
| ZINC000000000288 | -3.9 | ZINC000000000478 | -3.6 | ZINC000000000562 | -3.4 | ZINC000000000368 | -3.2 | ZINC000000000285 | -3 | ZINC000000000378 | -2.7 |
| ZINC000000000504 | -3.9 | ZINC000000000587 | -3.6 | ZINC000000000570 | -3.4 | ZINC000000000380 | -3.2 | ZINC000000000311 | -3 | ZINC000000000407 | -2.7 |
| ZINC000000000544 | -3.9 | ZINC000000000596 | -3.6 | ZINC000000000581 | -3.4 | ZINC000000000388 | -3.2 | ZINC000000000325 | -3 | ZINC000000000411 | -2.7 |
| ZINC000000000585 | -3.9 | ZINC000000000605 | -3.6 | ZINC000000000594 | -3.4 | ZINC000000000389 | -3.2 | ZINC000000000387 | -3 | ZINC000000000486 | -2.7 |
| ZINC000000000052 | -3.8 | ZINC000000000010 | -3.5 | ZINC000000000607 | -3.4 | ZINC000000000394 | -3.2 | ZINC000000000392 | -3 | ZINC000000000487 | -2.7 |
| ZINC000000000073 | -3.8 | ZINC000000000011 | -3.5 | ZINC000000000051 | -3.3 | ZINC000000000416 | -3.2 | ZINC000000000404 | -3 | ZINC000000000513 | -2.7 |
| ZINC000000000077 | -3.8 | ZINC000000000012 | -3.5 | ZINC000000000076 | -3.3 | ZINC000000000425 | -3.2 | ZINC000000000436 | -3 | ZINC000000000602 | -2.7 |
| ZINC000000000144 | -3.8 | ZINC000000000145 | -3.5 | ZINC000000000124 | -3.3 | ZINC000000000438 | -3.2 | ZINC000000000472 | -3 | ZINC000000000353 | -2.6 |
| ZINC000000000171 | -3.8 | ZINC000000000148 | -3.5 | ZINC000000000128 | -3.3 | ZINC000000000450 | -3.2 | ZINC000000000490 | -3 | ZINC000000000567 | -2.6 |
| ZINC000000000176 | -3.8 | ZINC000000000169 | -3.5 | ZINC000000000133 | -3.3 | ZINC000000000455 | -3.2 | ZINC000000000552 | -3 | ZINC000000000083 | -2.5 |
| ZINC000000000181 | -3.8 | ZINC000000000187 | -3.5 | ZINC000000000136 | -3.3 | ZINC000000000456 | -3.2 | ZINC000000000558 | -3 | ZINC000000000412 | -2.5 |
| ZINC000000000277 | -3.8 | ZINC000000000192 | -3.5 | ZINC000000000137 | -3.3 | ZINC000000000463 | -3.2 | ZINC000000000591 | -3 | ZINC000000000560 | -2.5 |
| ZINC000000000340 | -3.8 | ZINC000000000240 | -3.5 | ZINC000000000174 | -3.3 | ZINC000000000488 | -3.2 | ZINC000000000007 | -2.9 | ZINC000000000593 | -2.4 |
| ZINC000000000374 | -3.8 | ZINC000000000243 | -3.5 | ZINC000000000179 | -3.3 | ZINC000000000495 | -3.2 | ZINC000000000021 | -2.9 | ZINC000000000045 | -2.3 |
| ZINC000000000397 | -3.8 | ZINC000000000254 | -3.5 | ZINC000000000184 | -3.3 | ZINC000000000510 | -3.2 | ZINC000000000023 | -2.9 | ZINC000000000345 | -2.3 |
| ZINC000000000431 | -3.8 | ZINC000000000283 | -3.5 | ZINC000000000189 | -3.3 | ZINC000000000542 | -3.2 | ZINC000000000036 | -2.9 | ZINC000000000015 | -2.2 |
| ZINC000000000432 | -3.8 | ZINC000000000293 | -3.5 | ZINC000000000190 | -3.3 | ZINC000000000550 | -3.2 | ZINC000000000053 | -2.9 | ZINC000000000573 | -1.9 |
| ZINC000000000505 | -3.8 | ZINC000000000297 | -3.5 | ZINC000000000221 | -3.3 | ZINC000000000556 | -3.2 | ZINC000000000056 | -2.9 | ZINC000000000457 | -1.8 |
| ZINC000000000509 | -3.8 | ZINC000000000348 | -3.5 | ZINC000000000246 | -3.3 | ZINC000000000559 | -3.2 | ZINC000000000078 | -2.9 |  |  |
| ZINC000000000527 | -3.8 | ZINC000000000391 | -3.5 | ZINC000000000282 | -3.3 | ZINC000000000565 | -3.2 | ZINC000000000088 | -2.9 |  |  |
| ZINC000000000583 | -3.8 | ZINC000000000435 | -3.5 | ZINC000000000296 | -3.3 | ZINC000000000586 | -3.2 | ZINC000000000093 | -2.9 |  |  |
| ZINC000000000590 | -3.8 | ZINC000000000473 | -3.5 | ZINC000000000334 | -3.3 | ZINC000000000604 | -3.2 | ZINC000000000130 | -2.9 |  |  |
| ZINC000000000039 | -3.7 | ZINC000000000474 | -3.5 | ZINC000000000350 | -3.3 | ZINC000000000022 | -3.1 | ZINC000000000164 | -2.9 |  |  |
| ZINC000000000066 | -3.7 | ZINC000000000479 | -3.5 | ZINC000000000360 | -3.3 | ZINC000000000044 | -3.1 | ZINC000000000178 | -2.9 |  |  |
| ZINC000000000149 | -3.7 | ZINC000000000480 | -3.5 | ZINC000000000367 | -3.3 | ZINC000000000063 | -3.1 | ZINC000000000194 | -2.9 |  |  |
| ZINC000000000153 | -3.7 | ZINC000000000500 | -3.5 | ZINC000000000372 | -3.3 | ZINC000000000085 | -3.1 | ZINC000000000216 | -2.9 |  |  |
| ZINC000000000198 | -3.7 | ZINC000000000503 | -3.5 | ZINC000000000381 | -3.3 | ZINC000000000095 | -3.1 | ZINC000000000298 | -2.9 |  |  |
| ZINC000000000199 | -3.7 | ZINC000000000508 | -3.5 | ZINC000000000383 | -3.3 | ZINC000000000099 | -3.1 | ZINC000000000424 | -2.9 |  |  |
| ZINC000000000223 | -3.7 | ZINC000000000538 | -3.5 | ZINC000000000417 | -3.3 | ZINC000000000122 | -3.1 | ZINC000000000426 | -2.9 |  |  |
| ZINC000000000234 | -3.7 | ZINC000000000589 | -3.5 | ZINC000000000428 | -3.3 | ZINC000000000125 | -3.1 | ZINC000000000529 | -2.9 |  |  |
| ZINC000000000315 | -3.7 | ZINC000000000595 | -3.5 | ZINC000000000430 | -3.3 | ZINC000000000131 | -3.1 | ZINC000000000608 | -2.9 |  |  |
| ZINC000000000346 | -3.7 | ZINC000000000603 | -3.5 | ZINC000000000434 | -3.3 | ZINC000000000231 | -3.1 | ZINC000000000028 | -2.8 |  |  |
| ZINC000000000359 | -3.7 | ZINC000000000043 | -3.4 | ZINC000000000437 | -3.3 | ZINC000000000232 | -3.1 | ZINC000000000048 | -2.8 |  |  |
| ZINC000000000370 | -3.7 | ZINC000000000070 | -3.4 | ZINC000000000460 | -3.3 | ZINC000000000261 | -3.1 | ZINC000000000061 | -2.8 |  |  |
| ZINC000000000403 | -3.7 | ZINC000000000096 | -3.4 | ZINC000000000465 | -3.3 | ZINC000000000271 | -3.1 | ZINC000000000092 | -2.8 |  |  |
| ZINC000000000485 | -3.7 | ZINC000000000098 | -3.4 | ZINC000000000483 | -3.3 | ZINC000000000284 | -3.1 | ZINC000000000226 | -2.8 |  |  |
| ZINC000000000499 | -3.7 | ZINC000000000132 | -3.4 | ZINC000000000523 | -3.3 | ZINC000000000294 | -3.1 | ZINC000000000241 | -2.8 |  |  |
| ZINC000000000507 | -3.7 | ZINC000000000161 | -3.4 | ZINC000000000540 | -3.3 | ZINC000000000327 | -3.1 | ZINC000000000266 | -2.8 |  |  |
| ZINC000000000517 | -3.7 | ZINC000000000167 | -3.4 | ZINC000000000541 | -3.3 | ZINC000000000349 | -3.1 | ZINC000000000276 | -2.8 |  |  |
| ZINC000000000526 | -3.7 | ZINC000000000200 | -3.4 | ZINC000000000546 | -3.3 | ZINC000000000376 | -3.1 | ZINC000000000338 | -2.8 |  |  |
| ZINC000000000547 | -3.7 | ZINC000000000250 | -3.4 | ZINC000000000557 | -3.3 | ZINC000000000385 | -3.1 | ZINC000000000365 | -2.8 |  |  |
| ZINC000000000561 | -3.7 | ZINC000000000274 | -3.4 | ZINC000000000017 | -3.2 | ZINC000000000390 | -3.1 | ZINC000000000373 | -2.8 |  |  |
| ZINC000000000566 | -3.7 | ZINC000000000289 | -3.4 | ZINC000000000054 | -3.2 | ZINC000000000393 | -3.1 | ZINC000000000413 | -2.8 |  |  |

The Affinity of ligands from luteolin mol2 file docking with VEGFA

| name | Affinity (kcal/mol) | name | Affinity (kcal/mol) | name | Affinity (kcal/mol) | name | Affinity (kcal/mol) | name | Affinity (kcal/mol) | name | Affinity (kcal/mol) |
| --- | --- | --- | --- | --- | --- | --- | --- | --- | --- | --- | --- |
| ZINC000000000152 | -8.7 | ZINC000000000508 | -7.3 | ZINC000000000529 | -6.9 | ZINC000000000076 | -6.4 | ZINC000000000404 | -6.1 | ZINC000000000273 | -5.6 |
| ZINC000000000583 | -8.7 | ZINC000000000605 | -7.3 | ZINC000000000542 | -6.9 | ZINC000000000085 | -6.4 | ZINC000000000507 | -6.1 | ZINC000000000353 | -5.6 |
| ZINC000000000181 | -8.6 | ZINC000000000100 | -7.2 | ZINC000000000021 | -6.8 | ZINC000000000133 | -6.4 | ZINC000000000551 | -6.1 | ZINC000000000437 | -5.6 |
| ZINC000000000052 | -8.4 | ZINC000000000154 | -7.2 | ZINC000000000198 | -6.8 | ZINC000000000137 | -6.4 | ZINC000000000565 | -6.1 | ZINC000000000486 | -5.6 |
| ZINC000000000554 | -8.3 | ZINC000000000283 | -7.2 | ZINC000000000200 | -6.8 | ZINC000000000148 | -6.4 | ZINC000000000586 | -6.1 | ZINC000000000602 | -5.6 |
| ZINC000000000347 | -8.2 | ZINC000000000397 | -7.2 | ZINC000000000214 | -6.8 | ZINC000000000150 | -6.4 | ZINC000000000607 | -6.1 | ZINC000000000078 | -5.5 |
| ZINC000000000424 | -8.2 | ZINC000000000406 | -7.2 | ZINC000000000234 | -6.8 | ZINC000000000187 | -6.4 | ZINC000000000044 | -6 | ZINC000000000158 | -5.5 |
| ZINC000000000253 | -8.1 | ZINC000000000410 | -7.2 | ZINC000000000254 | -6.8 | ZINC000000000190 | -6.4 | ZINC000000000072 | -6 | ZINC000000000215 | -5.5 |
| ZINC000000000259 | -8.1 | ZINC000000000544 | -7.2 | ZINC000000000284 | -6.8 | ZINC000000000235 | -6.4 | ZINC000000000093 | -6 | ZINC000000000261 | -5.5 |
| ZINC000000000574 | -8.1 | ZINC000000000561 | -7.2 | ZINC000000000385 | -6.8 | ZINC000000000246 | -6.4 | ZINC000000000098 | -6 | ZINC000000000325 | -5.5 |
| ZINC000000000080 | -8 | ZINC000000000589 | -7.2 | ZINC000000000039 | -6.7 | ZINC000000000255 | -6.4 | ZINC000000000122 | -6 | ZINC000000000373 | -5.5 |
| ZINC000000000391 | -8 | ZINC000000000043 | -7.1 | ZINC000000000056 | -6.7 | ZINC000000000257 | -6.4 | ZINC000000000241 | -6 | ZINC000000000450 | -5.5 |
| ZINC000000000504 | -8 | ZINC000000000188 | -7.1 | ZINC000000000063 | -6.7 | ZINC000000000371 | -6.4 | ZINC000000000327 | -6 | ZINC000000000490 | -5.5 |
| ZINC000000000526 | -8 | ZINC000000000265 | -7.1 | ZINC000000000096 | -6.7 | ZINC000000000384 | -6.4 | ZINC000000000375 | -6 | ZINC000000000491 | -5.5 |
| ZINC000000000596 | -8 | ZINC000000000318 | -7.1 | ZINC000000000153 | -6.7 | ZINC000000000392 | -6.4 | ZINC000000000393 | -6 | ZINC000000000034 | -5.4 |
| ZINC000000000315 | -7.9 | ZINC000000000346 | -7.1 | ZINC000000000171 | -6.7 | ZINC000000000438 | -6.4 | ZINC000000000417 | -6 | ZINC000000000338 | -5.4 |
| ZINC000000000580 | -7.9 | ZINC000000000359 | -7.1 | ZINC000000000250 | -6.7 | ZINC000000000452 | -6.4 | ZINC000000000426 | -6 | ZINC000000000193 | -5.3 |
| ZINC000000000509 | -7.8 | ZINC000000000364 | -7.1 | ZINC000000000285 | -6.7 | ZINC000000000453 | -6.4 | ZINC000000000458 | -6 | ZINC000000000356 | -5.3 |
| ZINC000000000149 | -7.7 | ZINC000000000403 | -7.1 | ZINC000000000299 | -6.7 | ZINC000000000494 | -6.4 | ZINC000000000130 | -5.9 | ZINC000000000558 | -5.3 |
| ZINC000000000370 | -7.7 | ZINC000000000434 | -7.1 | ZINC000000000334 | -6.7 | ZINC000000000499 | -6.4 | ZINC000000000376 | -5.9 | ZINC000000000018 | -5.2 |
| ZINC000000000433 | -7.7 | ZINC000000000463 | -7.1 | ZINC000000000360 | -6.7 | ZINC000000000521 | -6.4 | ZINC000000000388 | -5.9 | ZINC000000000045 | -5.2 |
| ZINC000000000449 | -7.7 | ZINC000000000478 | -7.1 | ZINC000000000383 | -6.7 | ZINC000000000540 | -6.4 | ZINC000000000413 | -5.9 | ZINC000000000178 | -5.2 |
| ZINC000000000534 | -7.7 | ZINC000000000480 | -7.1 | ZINC000000000407 | -6.7 | ZINC000000000557 | -6.4 | ZINC000000000464 | -5.9 | ZINC000000000487 | -5.2 |
| ZINC000000000144 | -7.6 | ZINC000000000505 | -7.1 | ZINC000000000435 | -6.7 | ZINC000000000562 | -6.4 | ZINC000000000513 | -5.9 | ZINC000000000510 | -5.1 |
| ZINC000000000340 | -7.6 | ZINC000000000523 | -7.1 | ZINC000000000446 | -6.7 | ZINC000000000095 | -6.3 | ZINC000000000539 | -5.9 | ZINC000000000560 | -5 |
| ZINC000000000374 | -7.6 | ZINC000000000555 | -7.1 | ZINC000000000500 | -6.7 | ZINC000000000136 | -6.3 | ZINC000000000559 | -5.9 | ZINC000000000571 | -4.9 |
| ZINC000000000440 | -7.6 | ZINC000000000585 | -7.1 | ZINC000000000527 | -6.7 | ZINC000000000164 | -6.3 | ZINC000000000603 | -5.9 | ZINC000000000345 | -4.7 |
| ZINC000000000010 | -7.5 | ZINC000000000163 | -7 | ZINC000000000570 | -6.7 | ZINC000000000184 | -6.3 | ZINC000000000038 | -5.8 | ZINC000000000083 | -4.6 |
| ZINC000000000073 | -7.5 | ZINC000000000169 | -7 | ZINC000000000022 | -6.6 | ZINC000000000221 | -6.3 | ZINC000000000053 | -5.8 | ZINC000000000411 | -4.6 |
| ZINC000000000123 | -7.5 | ZINC000000000176 | -7 | ZINC000000000124 | -6.6 | ZINC000000000271 | -6.3 | ZINC000000000061 | -5.8 | ZINC000000000378 | -4.5 |
| ZINC000000000142 | -7.5 | ZINC000000000189 | -7 | ZINC000000000140 | -6.6 | ZINC000000000282 | -6.3 | ZINC000000000135 | -5.8 | ZINC000000000593 | -4.5 |
| ZINC000000000323 | -7.5 | ZINC000000000277 | -7 | ZINC000000000186 | -6.6 | ZINC000000000288 | -6.3 | ZINC000000000166 | -5.8 | ZINC000000000015 | -4.4 |
| ZINC000000000416 | -7.5 | ZINC000000000300 | -7 | ZINC000000000268 | -6.6 | ZINC000000000298 | -6.3 | ZINC000000000209 | -5.8 | ZINC000000000267 | -4.3 |
| ZINC000000000474 | -7.5 | ZINC000000000368 | -7 | ZINC000000000348 | -6.6 | ZINC000000000389 | -6.3 | ZINC000000000216 | -5.8 | ZINC000000000573 | -4 |
| ZINC000000000572 | -7.5 | ZINC000000000372 | -7 | ZINC000000000350 | -6.6 | ZINC000000000455 | -6.3 | ZINC000000000231 | -5.8 | ZINC000000000457 | -3.5 |
| ZINC000000000012 | -7.4 | ZINC000000000390 | -7 | ZINC000000000357 | -6.6 | ZINC000000000483 | -6.3 | ZINC000000000276 | -5.8 |  |  |
| ZINC000000000024 | -7.4 | ZINC000000000460 | -7 | ZINC000000000381 | -6.6 | ZINC000000000488 | -6.3 | ZINC000000000425 | -5.8 |  |  |
| ZINC000000000070 | -7.4 | ZINC000000000471 | -7 | ZINC000000000394 | -6.6 | ZINC000000000591 | -6.3 | ZINC000000000469 | -5.8 |  |  |
| ZINC000000000077 | -7.4 | ZINC000000000473 | -7 | ZINC000000000447 | -6.6 | ZINC000000000599 | -6.3 | ZINC000000000495 | -5.8 |  |  |
| ZINC000000000192 | -7.4 | ZINC000000000485 | -7 | ZINC000000000484 | -6.6 | ZINC000000000604 | -6.3 | ZINC000000000506 | -5.8 |  |  |
| ZINC000000000217 | -7.4 | ZINC000000000538 | -7 | ZINC000000000546 | -6.6 | ZINC000000000131 | -6.2 | ZINC000000000552 | -5.8 |  |  |
| ZINC000000000223 | -7.4 | ZINC000000000541 | -7 | ZINC000000000566 | -6.6 | ZINC000000000167 | -6.2 | ZINC000000000023 | -5.7 |  |  |
| ZINC000000000270 | -7.4 | ZINC000000000547 | -7 | ZINC000000000594 | -6.6 | ZINC000000000243 | -6.2 | ZINC000000000035 | -5.7 |  |  |
| ZINC000000000380 | -7.4 | ZINC000000000017 | -6.9 | ZINC000000000595 | -6.6 | ZINC000000000294 | -6.2 | ZINC000000000036 | -5.7 |  |  |
| ZINC000000000575 | -7.4 | ZINC000000000030 | -6.9 | ZINC000000000007 | -6.5 | ZINC000000000441 | -6.2 | ZINC000000000092 | -5.7 |  |  |
| ZINC000000000581 | -7.4 | ZINC000000000031 | -6.9 | ZINC000000000011 | -6.5 | ZINC000000000444 | -6.2 | ZINC000000000232 | -5.7 |  |  |
| ZINC000000000590 | -7.4 | ZINC000000000066 | -6.9 | ZINC000000000051 | -6.5 | ZINC000000000465 | -6.2 | ZINC000000000272 | -5.7 |  |  |
| ZINC000000000050 | -7.3 | ZINC000000000088 | -6.9 | ZINC000000000075 | -6.5 | ZINC000000000472 | -6.2 | ZINC000000000290 | -5.7 |  |  |
| ZINC000000000071 | -7.3 | ZINC000000000128 | -6.9 | ZINC000000000132 | -6.5 | ZINC000000000503 | -6.2 | ZINC000000000311 | -5.7 |  |  |
| ZINC000000000127 | -7.3 | ZINC000000000145 | -6.9 | ZINC000000000161 | -6.5 | ZINC000000000550 | -6.2 | ZINC000000000351 | -5.7 |  |  |
| ZINC000000000172 | -7.3 | ZINC000000000196 | -6.9 | ZINC000000000274 | -6.5 | ZINC000000000587 | -6.2 | ZINC000000000401 | -5.7 |  |  |
| ZINC000000000179 | -7.3 | ZINC000000000199 | -6.9 | ZINC000000000349 | -6.5 | ZINC000000000608 | -6.2 | ZINC000000000412 | -5.7 |  |  |
| ZINC000000000240 | -7.3 | ZINC000000000245 | -6.9 | ZINC000000000352 | -6.5 | ZINC000000000048 | -6.1 | ZINC000000000436 | -5.7 |  |  |
| ZINC000000000251 | -7.3 | ZINC000000000287 | -6.9 | ZINC000000000365 | -6.5 | ZINC000000000125 | -6.1 | ZINC000000000467 | -5.7 |  |  |
| ZINC000000000293 | -7.3 | ZINC000000000297 | -6.9 | ZINC000000000430 | -6.5 | ZINC000000000174 | -6.1 | ZINC000000000556 | -5.7 |  |  |
| ZINC000000000305 | -7.3 | ZINC000000000363 | -6.9 | ZINC000000000451 | -6.5 | ZINC000000000226 | -6.1 | ZINC000000000567 | -5.7 |  |  |
| ZINC000000000316 | -7.3 | ZINC000000000367 | -6.9 | ZINC000000000456 | -6.5 | ZINC000000000242 | -6.1 | ZINC000000000609 | -5.7 |  |  |
| ZINC000000000361 | -7.3 | ZINC000000000428 | -6.9 | ZINC000000000582 | -6.5 | ZINC000000000289 | -6.1 | ZINC000000000099 | -5.6 |  |  |
| ZINC000000000431 | -7.3 | ZINC000000000479 | -6.9 | ZINC000000000028 | -6.4 | ZINC000000000296 | -6.1 | ZINC000000000194 | -5.6 |  |  |
| ZINC000000000432 | -7.3 | ZINC000000000517 | -6.9 | ZINC000000000054 | -6.4 | ZINC000000000387 | -6.1 | ZINC000000000266 | -5.6 |  |  |

The Affinity of ligands from hyndarin mol2 file docking with Akt1

| name | Affinity (kcal/mol) |
| --- | --- |
| new_220268943 | -9.8 |
| new_220267944 | -9.4 |

The Affinity of ligands from hyndarin mol2 file docking with CXCL8

| name | Affinity (kcal/mol) |
| --- | --- |
| new_220267944 | -6.1 |
| new_220268943 | -5.8 |

The Affinity of ligands from hyndarin mol2 file docking with IL-6

| name | Affinity (kcal/mol) |
| --- | --- |
| new_220267944 | -6.3 |
| new_220268943 | -6.0 |

The Affinity of ligands from hyndarin mol2 file docking with JUN

| name | Affinity (kcal/mol) |
| --- | --- |
| new_220268943 | -6.1 |
| new_220267944 | -5.7 |

The Affinity of ligands from hyndarin mol2 file docking with MAPK1

| name | Affinity (kcal/mol) |
| --- | --- |
| new_220267944 | -8.0 |
| new_220268943 | -7.1 |

The Affinity of ligands from hyndarin mol2 file docking with MAPK14

| name | Affinity (kcal/mol) |
| --- | --- |
| new_220267944 | -7.6 |
| new_220268943 | -7.5 |

The Affinity of ligands from hyndarin mol2 file docking with RB1

| name | Affinity (kcal/mol) |
| --- | --- |
| new_220267944 | -7.7 |
| new_220268943 | -7.3 |

The Affinity of ligands from hyndarin mol2 file docking with RELA

| name | Affinity (kcal/mol) |
| --- | --- |
| new_220268943 | -3.6 |
| new_220267944 | -3.5 |

The Affinity of ligands from hyndarin mol2 file docking with VEGFA

| name | Affinity (kcal/mol) |
| --- | --- |
| new_220267944 | -7.6 |
| new_220268943 | -7.6 |

The Affinity of ligands from beta-sitosterol mol2 file docking with Akt1

| name | Affinity (kcal/mol) |
| --- | --- |
| 562788026 | -11.8 |
| 556396181 | -11 |
| new_194390080 | -11 |
| 568758129 | -10.9 |
| 569862804 | -10.8 |
| 569862851 | -10.8 |
| 571845045 | -10.7 |
| 571845096 | -10.7 |
| 571845116 | -10.6 |

The Affinity of ligands from beta-sitosterol mol2 file docking with CXCL8

| name | Affinity (kcal/mol) |
| --- | --- |
| 568758129 | -6.8 |
| 556396181 | -6.7 |
| 571845045 | -6.6 |
| 562788026 | -6.5 |
| 569862804 | -6.5 |
| 569862851 | -6.5 |
| new_194390080 | -6.2 |
| 571845096 | -6 |
| 571845116 | -6 |

The Affinity of ligands from beta-sitosterol mol2 file docking with IL-6

| name | Affinity (kcal/mol) |
| --- | --- |
| 562788026 | -7.5 |
| new_194390080 | -7.1 |
| 556396181 | -6.7 |
| 568758129 | -6.6 |
| 569862851 | -6.5 |
| 571845116 | -6.5 |
| 569862804 | -6.4 |
| 571845045 | -6.4 |
| 571845096 | -6.4 |

The Affinity of ligands from beta-sitosterol mol2 file docking with JUN

| name | Affinity (kcal/mol) |
| --- | --- |
| 562788026 | -7.1 |
| 569862804 | -6.9 |
| new_194390080 | -6.9 |
| 568758129 | -6.8 |
| 569862851 | -6.7 |
| 556396181 | -6.6 |
| 571845116 | -6.5 |
| 571845096 | -6.3 |
| 571845045 | -6.2 |

The Affinity of ligands from beta-sitosterol mol2 file docking with MAPK1

| name | Affinity (kcal/mol) |
| --- | --- |
| 562788026 | -8.2 |
| 568758129 | -8.2 |
| 569862804 | -8.2 |
| 571845045 | -8 |
| 571845116 | -8 |
| 571845096 | -7.8 |
| 556396181 | -7.7 |
| new_194390080 | -7.6 |
| 569862851 | -7.4 |

The Affinity of ligands from beta-sitosterol mol2 file docking with MAPK14

| name | Affinity (kcal/mol) |
| --- | --- |
| 562788026 | -9 |
| 571845096 | -8.4 |
| 556396181 | -8.3 |
| 571845045 | -8.3 |
| new_194390080 | -8.3 |
| 569862851 | -8.2 |
| 569862804 | -8.1 |
| 568758129 | -8 |
| 571845116 | -7.7 |

The Affinity of ligands from beta-sitosterol mol2 file docking with RB1

| name | Affinity (kcal/mol) |
| --- | --- |
| 569862851 | -9.1 |
| 571845045 | -8.8 |
| 568758129 | -8.7 |
| 571845096 | -8.4 |
| new_194390080 | -8.3 |
| 562788026 | -8.1 |
| 569862804 | -7.8 |
| 556396181 | -7.6 |
| 571845116 | -7.5 |

The Affinity of ligands from beta-sitosterol mol2 file docking with RELA

| name | Affinity (kcal/mol) |
| --- | --- |
| 562788026 | -4.6 |
| 571845116 | -3.7 |
| 569862851 | -3.6 |
| 568758129 | -3.5 |
| 571845045 | -3.5 |
| new_194390080 | -3.5 |
| 569862804 | -3.4 |
| 556396181 | -3.3 |
| 571845096 | -3.2 |

The Affinity of 5-aminosalicylic acid docking with the key target genes

| target | Affinity (kcal/mol) |
| --- | --- |
| Akt1 | -6.0 |
| CXCL8 | -4.5 |
| IL-6 | -4.9 |
| JUN | -4.4 |
| MAPK1 | -5.5 |
| MAPK14 | -5.7 |
| RB1 | -5.5 |
| RELA | -3.2 |
| VEGFA | -5.7 |
